# Open removal models with temporary emigration and population dynamics to inform invasive animal management

**DOI:** 10.1002/ece3.9173

**Published:** 2022-08-17

**Authors:** Bradley Udell, Julien Martin, Christina Romagosa, Hardin Waddle, Fred Johnson, Bryan Falk, Amy Yackel Adams, Sarah Funck, Jennifer Ketterlin, Eric Suarez, Frank Mazzotti

**Affiliations:** ^1^ Wildlife Ecology and Conservation University of Florida Gainesville Florida USA; ^2^ Wetland and Aquatic Research Center U.S. Geological Survey Gainesville Florida USA; ^3^ Eastern Ecological Science Center U.S. Geological Survey Laurel Maryland USA; ^4^ Department of Bioscience Aarhus University Rønde Denmark; ^5^ Fort Collins Science Center U.S. Geological Survey Fort Collins Colorado USA; ^6^ South Florida Natural Resources Center National Park Service Homestead Florida USA; ^7^ Florida Fish and Wildlife Conservation Commission West Palm Beach Florida USA; ^8^ Fort Lauderdale Research and Education Center University of Florida Davie Florida USA

**Keywords:** abundance, Argentine black and white tegu, availability, InfoPM, informed population model, invasive, population dynamics, removal, *Salvator merianae*

## Abstract

Removal sampling data are the primary source of monitoring information for many populations (e.g., invasive species, fisheries). Population dynamics, temporary emigration, and imperfect detection are common sources of variation in monitoring data and are key parameters for informing management. We developed two open robust‐design removal models for simultaneously modeling population dynamics, temporary emigration, and imperfect detection: a random walk linear trend model (estimable without ancillary information), and a 2‐age class informed population model (InfoPM, closely related to integrated population models) that incorporated prior information for age‐structured vital rates and relative juvenile availability. We applied both models to multiyear, removal trapping time‐series of a large invasive lizard (Argentine black and white tegu, *Salvator merianae*) in three management areas of South Florida to evaluate the effectiveness of management programs. Although estimates of the two models were similar, the InfoPMs generally returned more precise estimates, partitioned dynamics into births, deaths, net migration, and provided a decision support tool to predict population dynamics under different effort scenarios while accounting for uncertainty. Trends in tegu superpopulation abundance estimates were increasing in two management areas despite generally high removal rates. However, tegu abundance appeared to decline in the Core management area, where trapping density was the highest and immigration the lowest. Finally, comparing abundance predictions of no‐removal scenarios to those estimated in each management area suggested significant population reductions due to management. These results suggest that local tegu population control via systematic trapping may be feasible with high enough trap density and limited immigration; and highlights the value of these trapping programs. We provided the first estimates of tegu abundance, capture probabilities, and population dynamics, which is critical for effective management. Furthermore, our models are applicable to a wide range of monitoring programs (e.g., carcass recovery or removal point‐counts).

## Graphical abstract

This work extends removal abundance models to estimate animal abundance while accounting for imperfect detection, availability bias, and population dynamics (both within and between years). We develop two models, one that can be estimated without ancillary information, and a second (an informed population model, InfoPM) that also incorporates information on age‐structured vital rates. We apply these models to estimate the population dynamics (births, survival, migration) of an invasive lizard (Argentine black and white tegus) in three management areas in South Florida.

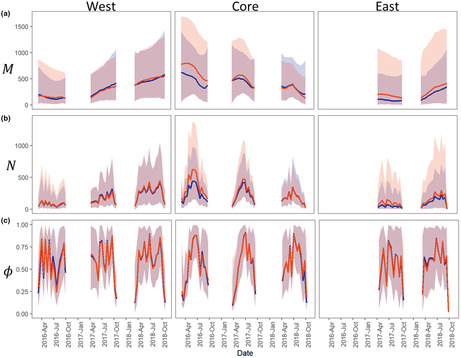

## 1 INTRODUCTION

Gaining a better understanding of wildlife population dynamics is critical for effective conservation management and ecological research. Monitoring programs (i.e., the repeated sampling of populations over time) provide the empirical data needed to estimate population abundance, trends, vital rates, and capture probabilities to evaluate the effectiveness of management programs and predict future population dynamics under different scenarios. Removal sampling, the repeated sampling of a population without replacement (Moran, 1951), is common as a monitoring technique (e.g., time‐removal counts, carcass recovery programs of marine mammals), and arises naturally from many conservation management programs such as invasive animal population control or harvest programs (i.e., hunting, fishing). In fact, in the case of invasive animal management, time series of removal data often represent the most relevant (or only) empirical monitoring data available to inform management efforts.

### 1.1 Challenges estimating abundance and population dynamics

Unfortunately, imperfect detection and other sources of observation error and process variation make analysis of raw removal data or other count indexes challenging (Anderson, 2001). Removal models (Moran, 1951; Zippen, 1956) are statistical abundance estimators that account for these biases when estimating abundance and removal probabilities from removal sampling data. They are structurally similar to other unmarked abundance estimators (e.g., N‐mixture models, Royle, 2004) and rely on the assumption of population closure (i.e., that a population remains constant between sampling events). Violations to population closure are common in natural systems and occur from demographic change (e.g., births and deaths, Dail & Madsen, 2011) and animal movement (e.g., permanent emigration/immigration, and temporary emigration [animals leaving the sample area temporarily], Chandler et al., 2011; Kendall et al., 1997), which result in biased estimators (Link, Converse, et al., 2018). Thus, it is important to develop models robust to both types of closure violations.

Temporary‐emigration/availability‐bias often arise in sampling designs from the partial overlap of the effective capture area of a sampling array with animal spatiotemporal use distributions (Chandler et al., 2011; Kendall et al., 1997). It is partially determined by seasonal and daily weather patterns and movement behavior and territoriality of the species being sampled. Availability bias can be heterogeneous within a removal time series due to changes in animal activity centers and movement behavior throughout the course of sampling, or from changes in spatial dispersion due to removing animals. Additionally, animal populations change throughout the course of a year due to population dynamics via births, deaths, and net migration. When both occur simultaneously in a removal time series, it can be difficult to determine if changes in removals over time are driven by imperfect detection (observation bias), temporary change (availability bias), or permanent change (demographics), and statistical models that seek to explicitly disentangle all these are often nonidentifiable (Zhou et al., 2019).

Originally formulated as mark‐recapture models with recapture probabilities of zero, recent removal models formulated as unmarked abundance estimators (as well as unmarked abundance estimators in general) have been improved to accommodate some of these issues (Rodriguez de Rivera & McCrea, 2021). This includes the use of: multinomial N‐mixture models as a general estimation procedure (Chandler et al., 2011; Dorazio et al., 2005; Kéry & Royle, 2015; Royle, 2004), open robust design to improve estimation when population closure is violated (Kéry et al., 2009; Link, Schofield, et al., 2018; Zhao & Royle, 2019; Zhou et al., 2019), models that consider the explicit dynamics from temporary emigration/availability bias (Chandler et al., 2011; Zhou et al., 2019), models with explicit population dynamics from demographic changes (Dail & Madsen, 2011; Matechou et al., 2016), and models with age‐structured population dynamics (Zipkin et al., 2014). However, even when using a robust design approach (which assumes multiple primary periods of closure, and within each there are temporally replicated sampling occasions to estimate the effective capture probability; Kendall et al., 1997), models that include both population dynamics and temporary emigration remain challenging and are often non‐identifiable (Zhou et al., 2019).

### 1.2 Two approaches to overcome estimation challenges

To overcome these issues, either (1) the population dynamics model must be simplified to a version that is estimable or (2) more realistic population dynamics models (with births and deaths) can be constructed with the aid of ancillary information. Here, we take both approaches and develop two models: the first a simplified, Bayesian random walk, linear‐trend time‐series model (Holmes et al., 2021) to approximate intra‐ and inter‐year population dynamics without requiring ancillary information, and the second an “Informed Population Model” (InfoPM) which is closely related to integrated population models (IPMs) (Schaub & Kéry, 2022), but uses informed priors rather than multiple data sets and likelihoods.

Bayesian random walk time series models, which can be formulated as state‐space models to account for observation error, provide a useful tool for time‐series estimation (Scheuerell et al., 2015). IPMs combine count data and matrix population models with ancillary data sets on vital rates (e.g., mark‐recapture data) into a single model by integrating multiple likelihoods (Schaub & Kery, 2021). Importantly, IPMs provide insights into the relative roles of births, deaths, and migration in age‐structured population dynamics and could be extended to account for age‐structured availability biases. In a sequential Bayesian paradigm, some or all of these ancillary data sets and likelihoods can be replaced by informed priors for the age‐structured vital rates while leading to comparable (and in some cases identical) inference (Schaub & Kery, 2021). In models where all additional sources of ancillary information are incorporated as informative priors (e.g., Millar & Meyer, 2000; Thomas et al., 2005) as opposed to additional data sets and likelihoods, they are more accurately described as informed population models (InfoPM) because they result in a single likelihood. When the best available prior information on vital rates is based on expert elicitation (e.g., Johnson et al., 2017), such models provide a formal mechanism for combined inference with the empirical removal data. For example, the use of all available information about processes of interest is commonplace in scientific learning, and Bayesian inference provides formal way to combine all available information in a rigorous way (Banner et al., 2020; Lemoine, 2019; Low Choy et al., 2009; Schaub & Kéry, 2022; Zipkin & Saunders, 2018).

Finally, even in models that account for temporary emigration, some portion of individuals will suffer mortality or permanently emigrate before ever becoming available, biasing removal probabilities high and abundance (and growth rates) estimates low (Kendall et al., 1997). This bias is particularly likely for populations with cryptic life stages, especially if such life stages also have low survival and comprise a significant portion of the populations (e.g., juvenile life stages of many species). Recent formulations of unmarked models for age‐structured data (e.g., Zipkin et al., 2014) could be extended to an InfoPM framework; however, if combined with ancillary information on age‐specific vital rates, the timing of the birth pulse, and relative juvenile availability, age structure can be modeled for two age classes even without age‐structured observations. In fact, the estimation of “hidden” or “extra” parameters for which there is no explicit data (e.g., age structure) is one of the main advantages of using an IPM (or InfoPM) approach (Schaub & Kéry, 2022). This approach is especially useful when age information is missing from removal programs, when, for example, animals are not recovered (e.g., Davis et al., 2016), age is difficult to estimate, acoustic surveys are used, or the captures of some age classes are too rare for estimation.

### 1.3 Motivating example and empirical application

We apply both models to the case study of Argentine black and white tegus (*Salvator merianae*), large invasive lizards established near the Everglades and other important ecological areas in Florida. Tegus were first introduced over 20 years ago (Meshaka et al., 2019), and the first breeding population in Miami‐Dade County was discovered in 2008 (Pernas et al., 2012). This initial population has since grown in abundance and spatial extent across a heterogeneous landscape to comprise a patchy population spanning now ~400 km^2^. As a nest predator with high fecundity, these animals have significant potential for ecological impacts in the Greater Everglades Ecosystem, including nest predation to threatened American crocodiles or nesting birds (Mazzotti et al., 2015). Because tegus hibernate in winter months (McEachern et al., 2015) and can take advantage of anthropogenic features and altered ecosystems, these lizards have the potential to invade much of the southern United States (Chiarello et al., 2010; Jarnevich et al., 2018; Klug et al., 2015). State and federal partners collectively work to control spatially patchy populations of breeding tegus in southern Florida across three management areas (to the west, central/south, and east of the initial point of invasion) through systematic removal trapping programs. As of December 2018, over 6000 individuals were removed from these management areas in total (Meshaka et al., 2019). These removal time series in each management area provide a source of empirical monitoring data to monitor tegu population trends, evaluate the effectiveness of management, and support future decision making.

However, analyzing tegu removal data sets is challenging due to issues of temporally heterogeneous availability bias and population dynamics. Tegus hibernate underground in burrows in the winter (McEachern et al., 2015), emerge in February and increase activity, breed from March–May, hatch from May–August, and decline in activity in the fall (Meshaka et al., 2019). In addition, tegu populations are demographically open throughout the year, with a birth pulse in the late spring/early summer (Meshaka et al., 2019), and mortality and net migration throughout the year. Tegu populations are age structured, with heterogeneities in vital rates and capture probabilities between life stages. Furthermore, estimating tegu age based on size is challenging (however see Meshaka et al., 2019) and the incidence of hatchling captures is generally low.

One recent population analysis based on expert elicitation (Johnson et al., 2017) suggested that the juvenile age class comprises a majority of the population at stable age distribution (68% of the population after the birth pulse). When combined with low survival rates and capture rates of juveniles (i.e., due to low juvenile availability), this presents complications for estimating temporary emigration (e.g., Kendall et al., 1997) because a large proportion of animals are juveniles, and of those many do not survive long enough (or permanently emigrate) before ever becoming available for capture. Consequently, we developed an age structured InfoPM based on unstructured removal data, prior information for age‐structured vital rates, and matrix algebra to scalarize the system. The InfoPM framework can also serve as a tool for conducting counterfactual analysis (e.g., Ferraro, 2009; Schaub & Kéry, 2022) by quantifying the impacts of management interventions, for example, by modeling no‐removal (nonintervention) scenarios and comparing predicted abundances to the estimated abundances over time. Importantly, this also accounts for future survival and reproductive processes when evaluating the efficacy of removal programs.

In summary, we develop novel models using an open‐robust design removal framework to estimate the superpopulation size (the total number of animals with home ranges overlapping an effective trapping region), population dynamics, availability bias, and capture probabilities of animals from removal trapping time series. We apply these models to the case study of tegu removal trapping in three management areas in the Greater Everglades Ecosystem in Florida using three years (2016–2018) of data collected from systematic trapping programs. We provide the first empirical population estimates and capture probabilities of tegus in each management area. By estimating trends and annual removal probabilities in addition to no‐removal scenarios, we also provide rigorous evaluation of the efficacy of removal programs.

## 2 METHODS

### 2.1 Data collection and study areas

**FIGURE 1:**
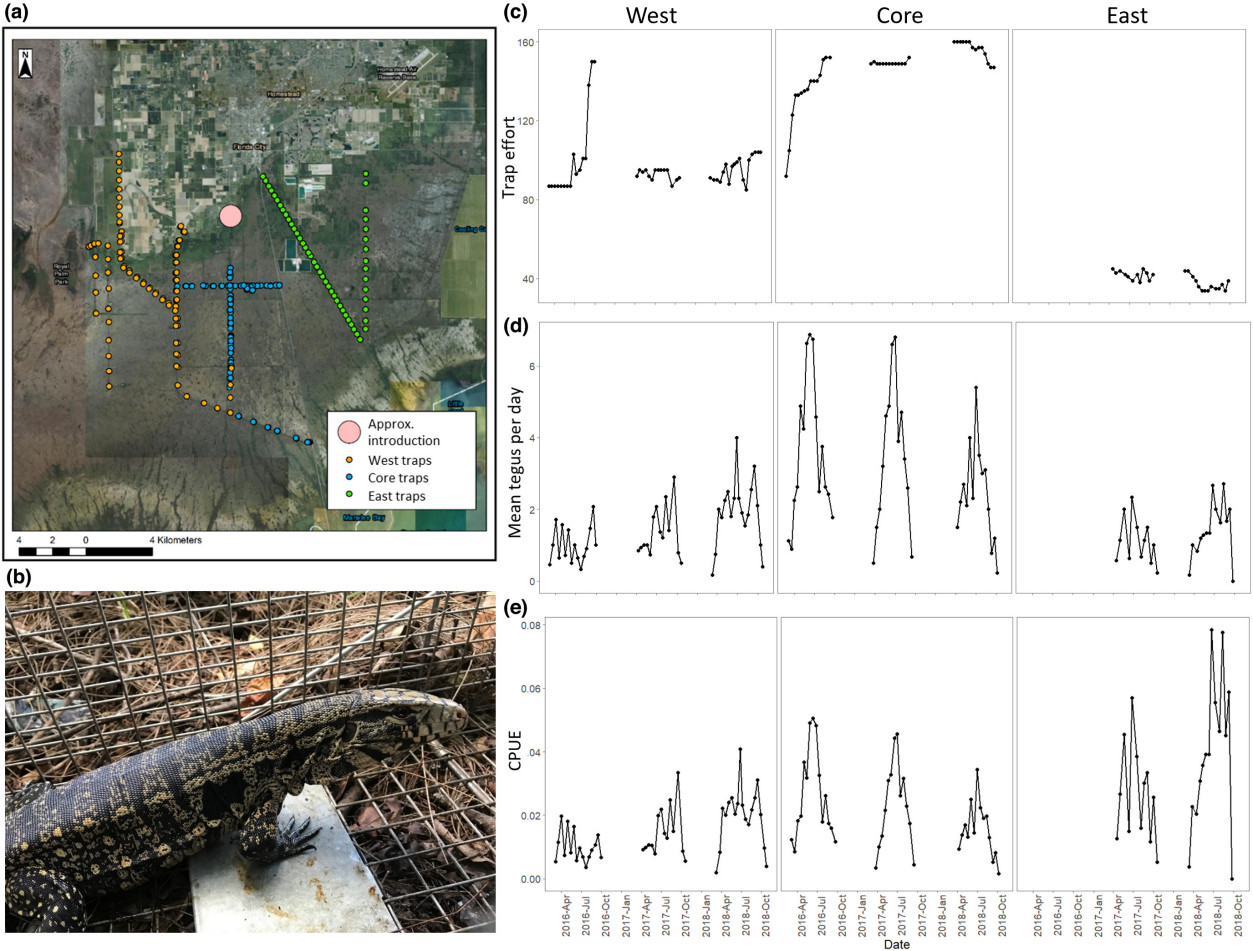
A map of tegu trap lines in 2017 (the first year of trapping in the east), a tegu in a trap, and the bi‐weekly trap effort and mean captures for each management area. (a) Trap locations and management areas representing the collective efforts of all partners in 2017 to study, contain, and control tegus. The pink circle represents the approximate location where the population was first recognized as established in 2008. The Core management area is largely located in the Southern Glades marshland south of Homestead, the West management area represents the boundary lands outside Everglades National Park, and the East management area consist of two major roads between the initial invasion site and Turkey Point Power Plant. In general, traps are deployed along levies, canals, and roads. Some of these sites are in raised habitat within a matrix of seasonally inundated wetlands and marshes, and other sites are within a matrix of natural and agricultural lands. (b) an adult tegu caught in a live trap, baited with a chicken egg (photo credit: Dan Quinn). (c) bi‐weekly trap effort (mean traps per day), (d) capture data (mean tegus per day), and (e) CPUE in each management area.

Tegu removal data were collected from systematic trapping programs in three areas (Core, East, West) where trapping is carried out by multiple agency partners (Appendix A and Figure 1). Tegu trap types, modifications, and number of traps deployed each week varied by location and year (see Appendix A for full description of trapping methods in each location). Trap lines in each location consisted of wire live traps (Havahart and Tomahawk) of various sizes baited with chicken eggs, and each trap is designed to capture a single animal. Traps were sometimes modified to increase tegu capture probability (Appendix A). Traps were deployed (often in pairs) alongside roads, canals, and levees, and in other vegetated and shaded locations. When surrounded by marsh or wet‐prairie habitat, tegus appear to spend more time in drier, higher‐elevation areas (Klug et al., 2015), and configuration of suitable habitat varied by locations. For example, suitable habitat in the Core area is mostly located along linear features interspersed within mostly unsuitable sawgrass marsh habitat. While these features dominate in the West and East areas, they are often adjacent to other suitable agricultural or natural habitat (Figure 1, Appendix A), including some that are ecologically sensitive (e.g., pine rockland habitats in Everglades National Park). Traps were deployed daily from Feb‐Oct each year with the exception of closures due to hurricanes or *force majeure*, and daily effort varied by year and area (Appendix A).

### 2.2 Modeling approach

We used an open robust design framework (Kendall et al., 1997; Pollock et al., 1990) to structure temporal dynamics, assuming time is divided into primary sampling periods t each consisting of multiple secondary occasions *j*, across which the population is assumed closed, and between which the population changes. Such an approach has been previously applied to estimate abundance, population trends, and detection in unmarked populations (Kéry et al., 2009). While such models often represent interyear dynamics, robust design has also been applied to approximate intrayear dynamics resulting from temporary emigration in removal models (Zhou et al., 2019) by subdividing removal effort throughout a year into multiple primary periods (Table 1).

**TABLE 1:** Parameter symbols and definitions for both models

Parameter	Definition	Model
$M_{y,t}$	Superpopulation abundance	Both
$\lambda_{y,t}$	Poisson abundance rate	Both
$\phi_{y,t}$	Availability bias	Both
$N_{y,t,j}$	Available abundance (day and period)	Both
${\hat{N}}_{y,t}$	Available population abundance	Both
$\pi_{y,\mathrm{tj}}$	Multinomial probability vector	Both
$y_{y,\mathrm{tj}}$	Daily removals	Both
$p_{y,t}$	Daily capture probability per year and period	Both
$\theta_{y}$	Yearly capture efficiency	Both
$1-z_{\mathrm{pro}b_{y}}$	The daily zero‐inflation rate per year	Both
$z_{y,\mathrm{tj}}$	Daily temporal suitability rate	Both
$p\mathrm{se}t_{y,t}$	Period effective capture probability (superpopulation)	Both
$p_{\mathrm{yea}r_{y}}$	Yearly effective capture probability of the superpopulation	Both
$R_{y,t}$	Total removals each year and period	RW
$\Delta_{y,t}$	Random variable for change in superpopulation during each time step between periods for each year and period	RW
$\mu_{\text{tren}d_{y}}$	Mean of the normal distribution for time steps for periods	RW
$sd_{\text{tren}d_{y}}$	Standard deviation of the normal distribution for time steps for periods	RW
$\Delta_{\mathrm{yea}r_{y}}$	Random time step between years	RW
$sd_{\mathrm{yea}r_{y}}$	Standard deviation of the normal distribution for time steps for years	RW
$\lambda_{ad_{y,t}}$	Expected adult population each year and period	InfoPM
$s_{\mathrm{ad}}$	Period survival rate of adults	InfoPM
$s_{ad_{\text{year}}}$	Adult survival rate each year	InfoPM
${\mathrm{BtA}}_{y,t}$	Animals transitioning to the adult age class each period	InfoPM
$IE_{y,t}$	Net migration each year and period	InfoPM
$R_{ad_{y,t}}$	Expected adult removals each year and period	InfoPM
$bp_{y}$	Period of the birth pulse each year	InfoPM
$b_{y-1}$	Effective birth rate in year y‐1 including juvenile survival	InfoPM
$\lambda_{ad_{y-1,t=bp_{y-1}}}$	Adult abundance during at the birth pulse in year y‐1	InfoPM
$\mu_{IE_{y}}$	Mean net migration each period	InfoPM
$sd_{IE_{y}}$	Standard deviation of net migration each period	InfoPM
${\mathrm{pc}}_{ad_{y,t}}$	Relative catchability of adults to juveniles each period and year	InfoPM
${\mathrm{pc}}_{\mathrm{ju}v_{y,t}}$	Relative catchability of juveniles to adults each period and year	InfoPM
$s_{\mathrm{juv}}$	Juvenile annual survival in year y‐1	InfoPM
$R_{{\mathrm{juv}}_{y,t}}$	Expected juvenile removals each year and period	InfoPM
${\lambda_{\mathrm{juv}}}_{y,t}$	Expected juvenile abundance each year and period	InfoPM
$np_{y,t}$	Number of periods since the birth pulse each period and year	InfoPM
$\mathrm{Ma}x_{\text{period}}$	Maximum number of periods in a year cycle (26 for tegus)	InfoPM
$\phi_{{\mathrm{juv}}_{y,t}}$	Relative juvenile availability	InfoPM
$\mu_{\text{emerg}e_{y}}$	Mean of the relative juvenile availability distribution	InfoPM
$sd_{\text{emerg}e_{y}}$	Standard deviation of the relative juvenile availability distribution	InfoPM
$M_{y,t}^{*}$	Superpopulation abundance corrected for juvenile availability	InfoPM

In general, we envisioned a removal sampling time series for a trapping array made up of the total daily removals and total daily trap effort in each management area across multiple years. We assumed that temporary emigration resulted from partial overlap of home ranges with effective capture arrays, and that the probability of temporary emigration changes throughout the year as both the activity centers and sizes of animal home ranges shifted throughout the year (and as animals are removed throughout the year). The total number of animals with home‐ranges overlapping the effective trapping area within a primary period was termed the superpopulation, *M*_*t*_, and the total number of animals available for capture within the trapping area at a given primary period t and sampling occasion *j* was the available population $N_{\mathrm{tj}}$. Because the total trapping area in each management area was relatively consistent over the timeframe of interest (and data were not included from traps in later years that were deployed beyond the original capture area), we assumed that comparisons in the superpopulation from year to year were meaningful. However, if a measure of the effective capture area each year could be calculated then it could be used to adjust superpopulation size accordingly. We assumed the superpopulation abundance changes over time through births, deaths, and net migration throughout the year, which we approximated with different transition functions. Because the composition of traps and sampling locations vary among locations and years, we modeled a different average capture efficiency for each location and year. Then, given the total daily removals, capture efficiency, and total capture effort in each primary period, we estimated the daily capture probability of an entire trap array, and the abundance of the superpopulation and available population of animals corresponding to the total trapping area in each primary period.

We found after some preliminary investigation that a 2‐week sampling window provided a good compromise in our application to tegus, with removal rates high enough for rigorous estimation (e.g., Davis et al., 2016), and sampling windows reasonably short enough to assume population closure (other than removal and temporary migration) given the study system. In comparison, total removal rates were generally not high enough for reliable estimation when using 1‐week primary periods, whereas we viewed primary periods of 3 weeks or greater as too long for which to reasonably assume population closure. We subdivided the trapping season each year into two‐week primary periods $\left(t\right)$ for a total of 26 each year, each made up of multiple sampling occasions (i.e., capture days) *j*. Because the range and frequency of trapping days differed between management regions, the number of active trap days in each primary period and number of active primary periods each year differed among management areas (Appendix A). In our analysis, we only used data from March–October when tegus are most active to avoid estimation issues resulting from extreme rarity.

### 2.3 Observation model

We extended previously established unmarked methods for demographically closed populations with temporary emigration (Chandler et al., 2011) to open population dynamics using open robust design and transition functions for abundance over time. We assume that superpopulation abundance $\left(M_{t}\right)$ in each primary period comes from a point pattern process based on a Poisson distribution with rate parameter (expected abundance), $\lambda_{t}$.Here we develop the case of a single year but extend the logic for multiple years in later sections (e.g., $\lambda_{y,t}$ ).

(1) $$M_{t}\sim \text{Poisson}\left(\lambda_{t}\right)$$

We also assume that the available abundance $\left(N_{\mathrm{tj}}\right)\;$ each period t and day j comes from a binomial process given the superpopulation abundance $\left(M_{t}\right)$ and availability $\left(\phi_{t}\right)$ for each period, and that capture histories $\left(y_{\mathrm{tj}}\right)$ follow a multinomial process with $\pi_{\mathrm{tj}}$ as the multinomial cell probabilities for capture each day j and period t. Thus, the likelihood of the model can be specified using an analytical Poisson approximation (Dorazio et al., 2005; Kéry & Royle, 2015; Royle, 2004; Royle & Dorazio, 2008):

(2) $$y_{\mathrm{tj}}\sim \text{Poisson}\left(\phi_{t}*\pi_{\mathrm{tj}}*\lambda_{\mathrm{tj}}\right)$$

Here, $\lambda_{t}$ refers to the expected superpopulation abundance, $\phi_{t}*\pi_{\mathrm{tj}}$ is equal to the multinomial probability vector after accounting for availability, and $\pi_{\mathrm{tj}}$ is defined for each day j of each period t based on daily capture probability $\left(p_{t}\right)$ for each period:

(3) $$\pi_{\mathrm{tj}}=p_{t}*{\left(1-p_{t}\right)}^{j-1}$$

Because it is important to account for variation in removal effort (Davis et al., 2016; St. Clair et al., 2012), we model daily capture probability in each primary period $\left(p_{t}\right)$ based on the total effort (number of traps, ${\text{Traps}}_{t}$) and the yearly capture efficiency $\theta_{y}$ (i.e., the per unit effort probability of capture):

(4) $$p_{t}=1-{\left(1-\theta_{y}\right)}^{{\text{Traps}}_{t}}$$

where ${\left(1-\theta_{y}\right)}^{{\text{Traps}}_{t}}$ is the probability of not being trapped. Thus, while $p_{t}$ varies between primary periods based on the number of traps deployed in each, we assume that $\theta_{y}$ is constant within each year.

We estimated parameters for each management area separately. We assumed that capture efficiency $\left(\theta_{y}\right)$ varied by year and had a vague beta prior ($\theta_{y}\sim \text{Beta}\left(1,1\right)$). We also assumed the availability bias varied each primary period, with a vague beta prior:

(5) $$\phi_{t}\sim \text{Beta}\left(1,1\right)$$

Because there was a high incidence of zeros in the removal data, we accounted for zero‐inflation (i.e., temporal suitability, some days are not suitable capturing any individuals) assuming a Bernoulli random effect (Kéry & Royle, 2015) at each sampling occasion given the temporal suitability parameter $\left({z_{\text{prob}}}_{y}\right)$, and daily temporal replication for $z_{\mathrm{tj}}$ each within each year (Equation 6). We incorporated zero‐inflation into the likelihood using zero‐inflated Poisson model (Equation 7):

(6) $$z_{\mathrm{tj}}\sim \text{Bernoulli}\left({z_{\text{prob}}}_{y}\right)$$

(7) $$y_{\mathrm{tj}}\sim \text{Poisson}\left(\lambda_{t}*\phi_{t}*\pi_{\mathrm{tj}}*z_{\mathrm{tj}}\right)$$

We also calculated derived parameters for the total effective removal probabilities of the superpopulation each primary period (*p*set_*t*_), given daily capture rates (*p*_*t*_), availability (*ϕ*_*t*_), and temporal suitability $\left({z_{\text{prob}}}_{y}\right)$ as:

(8) $$p\mathrm{se}t_{t}=1-{\left(1-p_{t}*{z_{\text{prob}}}_{y}*\phi_{t}\right)}^{N\text{days}}$$

This rate is relevant for both management and for model estimability (e.g., Davis et al., 2016) as the effective removal rate of the superpopulation each primary period. We obtained annual removal rate of animals which are present in the superpopulation as:

(9) $$p_{\text{year}}=1-\prod \left(1-p\mathrm{se}t_{t}\right)$$

### 2.4 Population dynamics

We imposed a Markovian structure on the expected superpopulation abundance each primary period by specifying an autoregressive function, $\lambda_{t+1}=f\left(\lambda_{t}-R_{t}\right)$, where $R_{t}$ is the total number of animals removed each primary period. Population models such as the exponential or logistic could be specified for function f (Hostetler & Chandler, 2015). However, recent work on removal models that also include temporary emigration and robust design suggests that a transition function with births and survival is not identifiable without ancillary information (Zhou et al., 2019). Thus, either simplified transition models which can be estimated without ancillary information, or more complex demographic models which can incorporate ancillary information are required. We implemented both approaches.

The first approach uses a temporal random walk (RW) with a linear trend (e.g., Holmes et al., 2021), diffuse priors, and does not require ancillary information. These models are also structurally similar to previous work in modeling nonlinear population trends in expected value of unmarked populations under a robust design (Kéry et al., 2009). The second approach extends the first to an age‐structured, informed population model (InfoPM) using prior information on birth rates, survival rates, timing of the birth pulse, and relative availability of juveniles to construct a single combined likelihood for all removals. The second model estimates an uncorrected superpopulation abundance $\left(M\right)$ that is directly comparable to the estimate from the RW model, in addition to a second superpopulation abundance estimate $\left(M^{*}\right)$ that is corrected for age structured population dynamics and capture rates.

### 2.5 Random walk model

#### 2.5.1 Within year dynamics

The RW model (e.g., Holmes et al., 2017; Scheuerell et al., 2015) is a state‐space model that treats a time series of changes in abundance as random steps $\left(\Delta_{y,t}\right)$ that come from a common distribution (usually a Gaussian) with an underlying mean trend $\left(\mu_{\text{trend}}\right)$ and variance $\left(sd_{\text{trend}}^{2}\right)$. The linear trend is also termed the “drift” for each time step. Adding in an index y for year, these models have the general functional form of:

(10) $$\lambda_{y,t+1}=\lambda_{y,t}+\Delta_{y,t}$$

(11) $$\Delta_{y,t}\sim \text{Normal}\left(\mu_{\text{trend}},sd_{\text{trend}}\right)$$

Here the mean and standard deviation may vary based on temporal covariates (e.g., season, year). We develop two different RW models with differing assumptions for $\mu_{\text{trend}}$: a yearly trend model (where means and sd differ by year), and a seasonal trend model (where means differ by season and year, and sd varies by year).

We defined four seasons based on tegu biology (Meshaka et al., 2019): March–April (copulation/breeding), May–June (hatching), July–August (post‐hatching), and September–October (pre‐hibernation). We included the total removals each primary period $\left(R_{t}\right)$ into the transition function as follows:

(12) $$\lambda_{t}=\lambda_{t-1}+\Delta_{t}-R_{t}$$

(13) $${\Delta_{\lambda }}_{t}\sim \text{Normal}\left(\mu_{\text{trend}},sd_{\text{trend}}\right)$$

We specified vague normal priors for $\mu_{\text{trend}}$, and a weakly informative half Cauchy prior (e.g., Lemoine, 2019) for $sd_{\text{trend}}$ as: $sd_{\text{trend}}\sim \text{half}T\left(1,5\right)$.

#### 2.5.2 Between year dynamics

After the first year of sampling, we linked the time series between years (where $\lambda_{y,t=1}$ is the abundance in the first primary period of year y, and $\lambda_{y-1,t=T}$ is abundance in the last primary period of year −1) assuming a single random time step $\left(\Delta_{y}^{\text{year}}\right)$ between each set of years, each with independent normal priors:

(14) $$\lambda_{y,t=1}=\lambda_{y-1,t=T}+\Delta_{y}^{\text{year}}$$

(15) $$\Delta_{y}^{\text{year}}\sim \text{Normal}\left(0,sd_{y}^{\text{year}}\right)$$

Finally, we linked the primary periods between years as described above in Equation 14, assuming an independent step from a noninformative normal distribution. Given that tegus hibernate most of the time between trapping seasons (November to February), we assumed $sd_{\text{year}}$ was equal to 31.62 (precision = 0.001), which is a weakly informative prior that suggests the change in the superpopulation between the end of 1 year's trapping season and start of the next years trapping season will likely fall within $\pm 2*sd_{y}^{\text{year}}$ (approximately 63 individuals). A graphical depiction of the RW model (Figure E‐1) and example JAGS code are provided in Appendix E. See the corresponding data release for this publication (Waddle et al., 2022) for the full code and data sets.

### 2.6 2‐age class informed population model

#### 2.6.1 Model formulation

There are three primary limitations to the RW approach: (1) the changes in abundance are not partitioned into births, deaths, and net migration, (2) the model does not account for age structure or bias due to low juvenile availability (and thus underestimates population size and overestimates removal probabilities), and (3) the population cannot be reliably projected into the future under different effort scenarios. Our second approach built upon the RW model to develop a 2‐age class informed population model (InfoPM), by using prior information on age‐structured vital rates (productivity and survival probabilities) and juvenile availability to overcome these limitations.

Given that empirical data sets on tegu vital rates in the invaded range are nonexistent, our source of prior information was a previous work by Johnson et al. (2017), who used a 3‐point expert elicitation process with 11 species experts to estimate stochastic belief distributions for tegu age‐structured vital rates while quantifying uncertainty. While our use of an informed vs integrated population model was currently out of necessity, the same sequential Bayesian model structure could also be used when empirical estimates of vital rates become available. Because tegu removal counts were non‐age structured, we develop a single combined likelihood function for removals across all age classes. Furthermore, we formulated it so that we can estimate similar quantities and infer to degree to which estimates for abundance and removal rates are biased when we do not account for age structure and relative juvenile availability bias.

Building directly on methods and data from Johnson et al. (2017), we started with a post‐birth‐pulse, 4‐age class matrix model with juveniles, 1, 2, and 3+ years (breeders) individuals (Appendix B). We reformulated this model as a pre‐birth‐pulse Leslie matrix, where juveniles were assumed implicit (i.e., the census takes place before the birth pulse, thus only animals in age classes 1 year and older are explicit in the model) and used matrix algebra to scalarize this population model as a single age class (Appendix B). By combining this model with a prediction for the juvenile age class (given informed priors for vital rates and time since birth for each primary period), we formulated a 2‐age class model with an implicit juvenile class and an explicit ‘adult’ class of all animals 1 year and older.

The population model that we used to derive informative priors assumed yearly time steps, whereas the time step for the InfoPM models was a 2‐week period. Because we assumed a single birth pulse, we did not need to scale the birth rates; however, annual survival rates needed to be scaled to correspond with the length of the primary periods (14 days). We made the simplifying assumption that survival rates for each primary period were constant given the annual survival rate, although survival is likely to vary throughout the year (e.g., during winter). Accordingly, the adult survival rate scaled to the primary period $\left(s_{\mathrm{ad}}\right)$ was defined as ${s_{\mathrm{ad}}}_{\text{year}}^{\left(\frac{1}{\mathrm{Ma}x_{\text{period}}}\right)}$. We assumed 26 primary periods each year $\left(\mathrm{Ma}x_{\text{period}}=26\right)$, of which between 14–18 were sampled.

Given this formulation, we modeled the two state variables: the adult superpopulation $\left(\lambda_{ad_{y,t}}\right)$ which has a population dynamics function defining the transition between primary periods, and the juvenile superpopulation $\left(\lambda_{{\mathrm{juv}}_{y,t}}\right)$ for which we make a prediction every primary period. Because the functions for predicting $\lambda_{ad_{y,t}}$ and $\lambda_{{\mathrm{juv}}_{y,t}}$ depend on the observation/removal model to predict removals from each age class, we first describe the observation model before detailing the transition functions. A graphical depiction of the InfoPM model (Figure E‐1) and example JAGS code are provided in Appendix E.

#### 2.6.2 Observation removal model for 2‐age class mode

We defined relative juvenile availability $\phi_{{\mathrm{juv}}_{y,t}}$ as a proportion of adult availability, which ranged from zero at the time of birth pulse to one just before the transition to the adult age class. Thus, overall juvenile availability ($\phi_{y,t}*\phi_{{\mathrm{juv}}_{y,t}})$ also tracks changes in adult availability over time based on seasonal influences. We assumed that after accounting for differential availability, animals were captured with the same probability. Relative availability of juveniles was likely driven by a combination of factors including differences in movement behavior, prey preference (e.g., likelihood of responding to egg bait), risk tolerance, and size of individuals. We assumed that these differences disappeared entirely by the time juveniles transitioned to adults.

Matechou et al. (2016) modeled births in open removal models using the concept of “emergence groups” which become available for capture according to a cumulative normal distribution. We used a similar approach to model the relative juvenile availability process as a cumulative normal distribution, with the parameters $\mu_{\text{emerge}}$ (corresponding to the day since birth where the relative juvenile availability is equal to 0.5) and ${\mathrm{sd}}_{\text{emerge}}$ which controls the rate of increase. To include uncertainty in this relationship, we assume $\mu_{\text{emerge}}$ and ${\mathrm{sd}}_{\text{emerge}}$ are also random variables with prior distributions. Based on discussions with tegu biologist and patterns in age‐cohorts in recently published capture data (Meshaka et al., 2019), we assumed a uniform prior between 150 and 210 days since birth (with a mean of 180 days) for $\mu_{\text{emerge}}$, and a half normal distribution (mean = 60, precision = 0.005) for $sd_{\text{emerge}},$ with hyper‐parameters varying by year and management area.

Another way to envision the relative juvenile availability process is as a threshold rather than a proportion of adult availability, where $\phi_{\mathrm{juv}}$ refers the proportion of juveniles which are above the size threshold needed to be available with the same rate as adults (i.e., large enough to encounter and respond to a baited trap). Under this interpretation, half of juveniles would reach this size by $\mu_{\text{emerge}}$, and $\phi_{{\mathrm{juv}}_{y,t}}*{\lambda_{\mathrm{juv}}}_{y,t}$ is the number of animals catchable at the same rate as adults.

#### 2.6.3 Combined likelihood of adults and juveniles

We developed a combined likelihood for total removals given the values of each state variable, and the relative availability of each age class:

(16) $$y_{y,t,j}\sim \text{Poisson}\left(\pi_{y,t,j}*z_{y,t,j}*\phi_{y,t}*\lambda_{ad_{y,t}}+\pi_{y,t,j}*z_{y,t,j}*\phi_{y,t}*\phi_{{\mathrm{juv}}_{y,t}}*{\lambda_{\mathrm{juv}}}_{y,t}\right)$$

where the first part of the expected value (before the addition sign) corresponds to the expected number of adult removals, and second part (after the addition sign) corresponds to the expected number of juvenile removals. This model is comparable to the non‐age structured RW model, and can be rearranged as

(17) $$y_{y,t,j}\sim \text{Poisson}\left(\left(\lambda_{ad_{y,t}}+\phi_{{\mathrm{juv}}_{y,t}}*{\lambda_{\mathrm{juv}}}_{y,t}\right)*\pi_{y,t,j}*z_{y,t,j}*\phi_{y,t}\right)$$

Thus $\left(\lambda_{ad_{y,t}}+\phi_{{\mathrm{juv}}_{y,t}}*\lambda_{\mathrm{ju}v_{y,t}}\right)$ is equivalent to $\lambda_{y,t}$ (the expectation for $M_{y,t}$) in Equation 7, which is the superpopulation size estimate from the RW model that does not account for the bias from a cryptic juvenile stage. We denoted the superpopulation size corrected for juvenile bias as $M_{y,t}^{*}$, which had the expected value $\lambda_{y,t}^{*}=\lambda_{ad_{y,t}}+\lambda_{{\mathrm{juv}}_{y,t}}$. We made predictions for both $M_{y,t}$ and $M_{y,t}^{*}$ assuming a Poisson distribution with expectations $\left(\lambda_{ad_{y,t}}+\phi_{{\mathrm{juv}}_{y,t}}*\lambda_{\mathrm{ju}v_{y,t}}\right)$ and $\lambda_{y,t}^{*}$.

#### 2.6.4 Adjusting effective removal rates for $M_{t}^{*}$

We calculate the total effective capture probabilities each period $\left(p\mathrm{se}t_{y,t}^{*}\right)$, and annual capture probabilities $\left(p_{\mathrm{yea}r_{y}}^{*}\right)$ in each management area, adjusting for the proportion of catchable to uncatchable animals due to juvenile bias from relative availability and age distribution each period as

(18) $$p_{\text{catc}h_{y,t}}=\frac{{\lambda_{\mathrm{ad}}}_{y,t}+\phi_{{\mathrm{juv}}_{y,t}}*{\lambda_{\mathrm{juv}}}_{y,t}}{{\lambda_{\mathrm{ad}}}_{y,t}+{\lambda_{\mathrm{juv}}}_{y,t}}$$

(19) $$p\mathrm{se}t_{y,t}^{*}=1-{\left(1-p_{\text{catc}h_{y,t}}*p_{y,t}*z_{\mathrm{pro}b_{y}}*\phi_{y,t}\right)}^{N\mathrm{day}s_{t}}$$

(20) $$p_{\mathrm{yea}r_{y}}^{*}=1-\prod \left(1-p\mathrm{se}t_{y,t}^{*}\right)$$

#### 2.6.5 Allocating adult and juvenile removals

Given the superpopulation of adults and juveniles in each period, along with the juvenile availability $\phi_{{\mathrm{juv}}_{y,t}}$, we calculate the expected age distribution of catchable animals that are adults $\left({\mathrm{pc}}_{y,t}^{\mathrm{ad}}\right)$ compared to juveniles (i.e., the proportion of catchable animals that are adults), which we used to calculate the expected number of removals in each age class as

(21) $${\mathrm{pc}}_{y,t}^{\mathrm{ad}}=\frac{{\lambda_{\mathrm{ad}}}_{y,t}}{{\lambda_{\mathrm{ad}}}_{y,t}+\phi_{{\mathrm{juv}}_{y,t}}*{\lambda_{\mathrm{juv}}}_{y,t}}$$

(22) $${\mathrm{pc}}_{y,t}^{\mathrm{juv}}=1-{\mathrm{pc}}_{ad_{t}}$$

where adult removals are defined as $R_{ad_{y,t}}={\mathrm{pc}}_{y,t}^{\mathrm{ad}}*R_{y,t}$ and juvenile removals are defined as: $R_{\mathrm{ju}v_{y,t}}={\mathrm{pc}}_{y,t}^{\mathrm{juv}}*R_{y,t}$.

#### 2.6.6 Adult population dynamics between primary periods

The population dynamic function for the adult age class for each year and primary period was defined as

(23) $$\lambda_{ad_{y,t+1}}=\lambda_{ad_{y,t}}*s_{\mathrm{ad}}+IE_{y,t}-R_{ad_{y,t}}+{\mathrm{BtA}}_{y,t}$$

where $\lambda_{ad_{y,t+1}}$ and $\lambda_{ad_{y,t}}$ are the expected number of adults in year *y* and period *t +* 1 and period *t*, respectively. $s_{\mathrm{ad}}$ is the scalarized adult survival probability between primary periods that accounts for age‐structured survival rate and the stable age distribution of animals 1 year to 3+ years old (Appendix B). $IE_{y,t}$ is the net migration between primary periods, $R_{ad_{y,t}}$ is a prediction for the number of adults removed in year y and primary period *t*, and ${\mathrm{BtA}}_{y,t}$ is the number of juveniles that transition to adults in each year and primary period, which is zero except for when t is the primary period the birth pulse each year. We assume net migration $\left(IE_{y,t}\right)$ follows a RW process with vague priors:

(24) $$IE_{y,t}\sim \text{Normal}\left(\mu_{IE_{y}},sd_{IE_{y}}\right)$$

We developed models with the same assumptions for $IE_{y,t}$ as we did for the $\Delta_{y,t}$ in our set of RW models. $R_{ad_{y,t}}$ in Equation 23 is the expected number of adult removals each year and primary period given the total number of removals $R_{y,t}$ and the proportion of catchable animals that are adults each primary period $\left({\mathrm{pc}}_{y,t}^{\mathrm{ad}}\right)$, where ${\mathrm{pc}}_{ad_{y,t}}$ is based on age structure and juvenile availability each primary period (Equation 21). ${\mathrm{BtA}}_{y,t}$ is the number of tegus transitioning to *year 1* individuals each primary period, which is zero in all periods except for the anniversary of the birth pulse $\left(t=bp_{t}\right)$:

(25) $${\mathrm{BtA}}_{y,t}=I\left(y,t=bp_{y}\right)*\left(B_{y}-R_{\mathrm{ju}v_{y}}^{\text{total}}\right)$$

(26) $$B_{y}=b_{y-1}*\lambda_{ad_{y-1,t=bp_{y-1}}}$$

where $B_{y}$ is the effective birth cohort after juvenile survival before accounting for removals, (i.e., the total size of the juvenile cohort that was born in year *y* − 1, that also survived the until the birth pulse in year y). $B_{y}$ is calculated based on $b_{y-1}$, the effective birth rate of the previous year (including juvenile survival), and the adult population at the time of the birth pulse in the previous year. We used informative priors for $b_{y-1}$ each year (Appendix B). $R_{\mathrm{ju}v_{y}}^{\text{total}}$ is a prediction for the total number of juveniles that have been removed since the birth pulse in year y−1, and it used to correct the size of the effective juvenile cohort transitioning to adults $\left({\mathrm{BtA}}_{y,t}\right)$.

We considered May 1st to be the anniversary date of the birth pulse, when juveniles begin hatching from eggs. We estimated the initial superpopulation abundance of adults ${\lambda_{\mathrm{ad}}}_{y=1,t=1}$ in the first year of sampling assuming a vague prior, $\text{Gamma}\left(0.5,0.000001\right)$, which approximates Jeffrey's prior for a Poisson rate parameter (Lunn et al., 2012). The birth cohort in the first year of sampling $B_{y=1}$ is dependent on the adult population in the year before the first year of removal data $\left(\lambda_{ad_{0,t=bp_{y-1}}}\right)$, and we use a uniform prior set to reasonable bounds for this parameter for each management area (e.g., between 0 and 1500 in the Core area).

#### 2.6.7 Juvenile abundance each primary period

We make a prediction for the expected abundance of the juvenile cohort each primary period $\left({\lambda_{\mathrm{juv}}}_{y,t}\right)$ based on the expected size of the initial cohort after birth, the juvenile survival rate, and a prediction for the total number of juveniles removed since the birth pulse:

(27) $${\lambda_{\mathrm{juv}}}_{y,t}=\frac{B_{y}}{s_{\mathrm{ju}v_{y}}^{\gamma_{y,t}}}-\sum_{t=\mathrm{bp}}^{t}R_{{\mathrm{juv}}_{y,t}}$$

The first term in Equation 27, $\left(\frac{B_{y}}{s_{\mathrm{ju}v_{y}}^{\gamma_{y,t}}}\right)$, is a prediction given the expected abundance of the effective birth cohort in year *y* $\left(B_{y}\right)\;$, scaled by the annual juvenile survival rate $\left(s_{\mathrm{juv}}\right)$, and exponential term $\left(\gamma_{y,t}=\frac{\left(\mathrm{Ma}x_{\text{period}}-\omega_{y,t}\right)}{\mathrm{Ma}x_{\text{period}}}\right)$ that adjusts $s_{\mathrm{ju}v_{y}}$ to account for the number of periods since the birth pulse $\left(\omega_{y,t}\right)$. When the number of primary periods since birth is zero, this term reduces to $\frac{B_{y}}{s_{\mathrm{ju}v_{y-1}}}$, or the initial number of juveniles born, and after 26 periods ($np_{t}=\mathrm{Ma}x_{\text{period}})$ it reduces to $B_{y}$, the number of juvenile that survive and transition to adults (before considering juvenile removals).

### 2.7 No‐removal scenario projections

We developed a “no‐removal” scenario for each InfoPM model that served as a counterfactual example to which to compare the observed population trajectory and to quantify the effectiveness of management. The ‘no‐removal’ scenario was modeled assuming the same survival rates, birth rates, and net migration each year and primary period as for observed scenario, only without the subtraction of removals from the population each period (i.e., dropping $R_{ad_{y,t}}$ and $R_{{\mathrm{juv}}_{y,t}}$ from Equations 23 and 27 and $R_{\mathrm{ju}v_{y}}^{\text{total}}$ from Equation 25). We did this within the MCMC model fitting procedure by cloning all abundance and birth cohort variables and dropping the removal terms (Appendix E). Animals that are were not removed contribute to the future superpopulation size in the no‐removal scenario as long as they survive and also contribute the birth cohort in subsequent years.

### 2.8 Model evaluation

We estimated the parameters of all variants of both models types (RW and InfoPM) separately for each management area and determined the best supported model within each model class based on fit to the data and model parsimony. We used posterior predictive checks as a goodness‐of‐fit test using the Freeman‐Tukey test statistic as a measure of and calculated the Bayesian *p*‐value and c‐hat (variance inflation factor) for each model and management area, where values near 0.5 and 1.0, respectively, correspond to a perfect fit (Kéry & Royle, 2015). For model selection, we used the Widely Applicable Information Criterion statistic, or WAIC (a fully Bayesian analog of AIC, Gelman et al., 2014; Hooten & Hobbes, 2015). WAIC is calculated starting with the computed log pointwise posterior predictive density and then adding a correction for effective number of parameters to adjust for overfitting (calculated as the sum of variances of individual terms in the log predictive density), where lower WAIC values are preferred. Between models with yearly vs yearly and seasonal differences in the mean trends, we defaulted to the simpler model (yearly) when WAIC scores were very close (delta‐WIAC <0.5 units). Because we used a single likelihood formulation for InfoPM (where all ancillary information came in as informative priors rather than additional data sets), the number of data points for each management area was the same as for the RW model. Furthermore, additional parameters of the InfoPM were informed with prior information. Thus, the total log‐likelihood values, effective number of parameters, and WAIC scores for the InfoPM models can be quite similar to the RW models. Although WAIC is appropriate for comparing and selecting the optimal prior specification in Bayesian models (e.g., with and without prior information, Gelman et al., 2014), we only use WAIC to select between models within each model class (i.e., RW vs InfoPM).

### 2.9 MCMC implementation

We used JAGS 4.3 (Plummer, 2003) through R version 3.4.3 (R core team, 2017), and the JagsUI package (Kellner, 2017) to implement our models. We estimated parameters for each management area separately. We initially conducted 10,000 iterations for adaptation, 100,000 for burn in, and 200,000 additional iterations after burn in using a thin rate of 10 and three parallel chains for a total of 60,000 samples. In some cases (e.g., the RW model for the Core management area), longer MCMC runs were required to achieve convergence (e.g., 1e^6^ total iterations with a thin rate of 20). We used visual inspection of MCMC chains and R‐hat to diagnose convergence, where R‐hat values <1.1 indicated convergence. Because posterior distributions for the abundance parameter tend to be positively skewed, we used the posterior median as a point estimator (e.g., Link, Converse, et al., 2018). Due to the asymmetry in these distributions, we also calculate the 95% credible intervals for superpopulation abundance and available abundance using the 95% highest posterior density interval, whereas for all other parameters we use a symmetric 95% credible interval based on the 2.5% and 97.5% quantiles.

### 2.10 Estimator post‐validation

We post‐validated our RW abundance estimator (which is based on empirical data only) under study conditions with a simulation study to assess bias and precision based on the point estimates for parameters in each location (e.g., Davis et al., 2016; Kéry & Royle, 2015; Zipkin & Saunders, 2018). After dropping simulations where the models did not converge, we evaluated 120 simulations each for the East and West area scenarios and 114 simulations for the Core area scenario. We calculated the bias distributions between point estimates from simulated data sets and the true values (using the point estimates for parameters from each management region) used when simulating the data sets and used the median as the measure of central tendency. We evaluate estimator accuracy for M using two different measures, the median bias for each parameter, and the proportion of point estimates within some threshold percentage (e.g., 20%) of the true value (see Appendix C for details).

## 3 RESULTS

**FIGURE 2:**
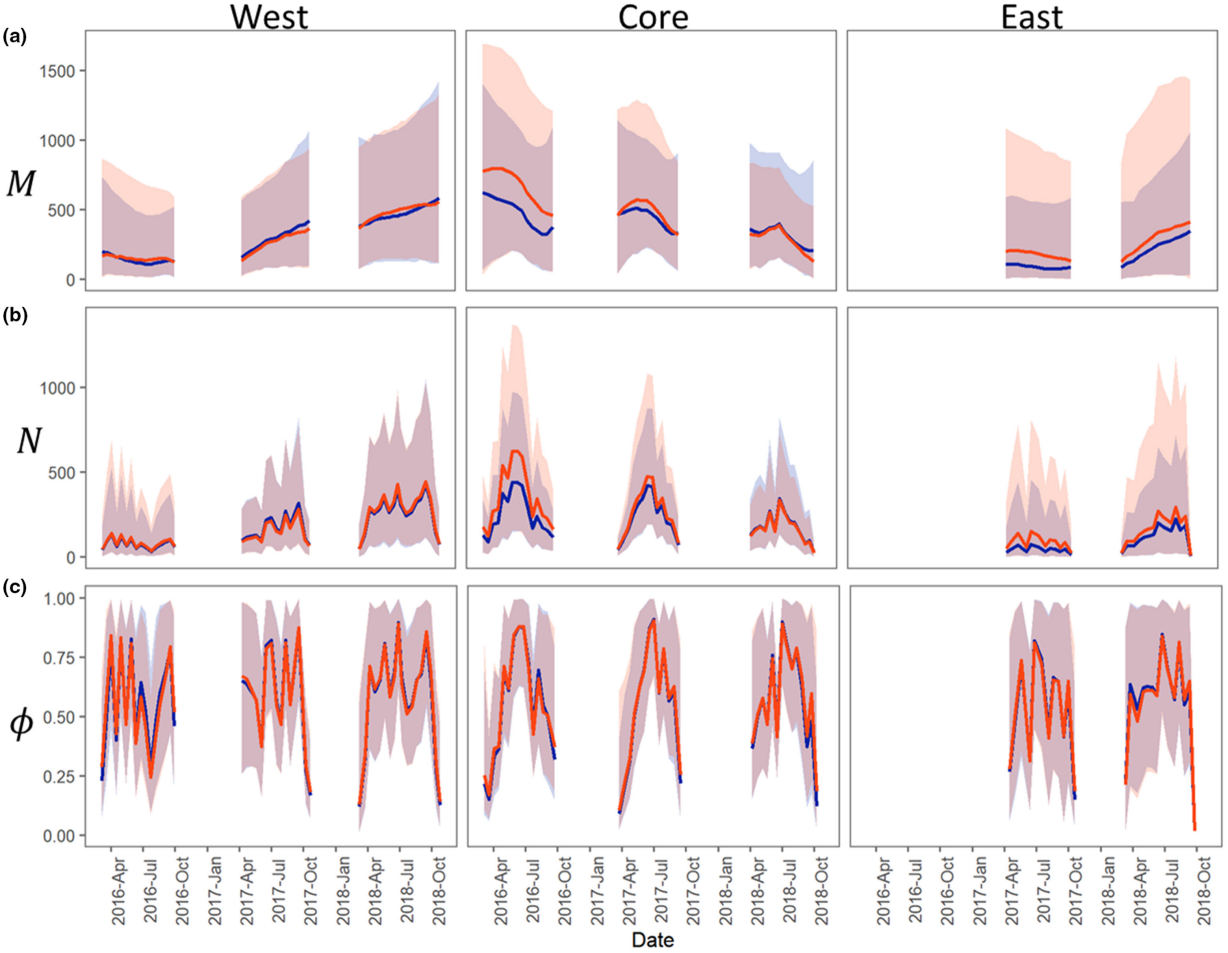
Estimates for superpopulation abundance $\left(M_{y,t}\right)$ (posterior medians and 95% CRIs), expected available abundance $\left({\hat{N}}_{y,t}\right)$ (posterior medians and 95% CRIs) and availability bias $\left(\phi_{y,t}\right)$ (posterior medians and 95% CRIs) in each management area using the best supported RW (orange) and InfoPM (blue) models. (a) Superpopulation abundance $\left(M_{y,t}\right)$ each primary period and year for each area, (b) mean available abundance $\left({\hat{N}}_{y,t}\right)$ each primary period and year for each area, and (c) availability bias $\left(\phi_{y,t}\right)$ each primary period and year for each area.

The RW and informed population models (InfoPM) had similar model fits (Table D‐7) and parameter estimates (Figure 2), and the top models in each management area showed no indication for lack of fit to the data (Table D‐7). The InfoPM estimates for *M* and *N* were generally more precise than for the RW except for the West management area (where estimates were equally precise), and for later part of 2018 in the Core region (Figure 2). The most parsimonious RW models and InfoPM models had mean trends (RW) and net migration (InfoPM) terms varying by year in the West and East areas and varying by year and season in the Core area (Table D‐7). In all cases, including zero inflation into the model to account for daily temporal suitability greatly improved model support and fit, while models without zero inflation often provided a poor fit to the data (Table D‐7).

In the West management area, superpopulation abundance (*M*) increased steadily from 2016 to 2018 (Figure 2). Superpopulation abundance also increased in the East management area from 2017 to 2018, especially in 2018 (Figure 2). In the Core area, however, *M* declined steadily over time with larger declines estimated for the RW than for the InfoPM (Figure 2). Based on these estimates, it appears that by the end of 2018 that *M* was higher in the West area than in the Core. In fact, abundance estimates in the West by the end of 2018 are similar those for the Core at the beginning of 2016. The mean available abundance in each area (*N*) tracks changes in *M* and availability bias ϕ over time, with lowest availability at the start (Feb–Mar) and end (Sept–Oct) of the trapping season and a peak in late spring and summer (May–June). Following the trend in superpopulation abundance and availability bias, the peaks in mean available abundance each year increased in the West and East areas over time and have decreased in the Core (Figure 2).

**FIGURE 3:**
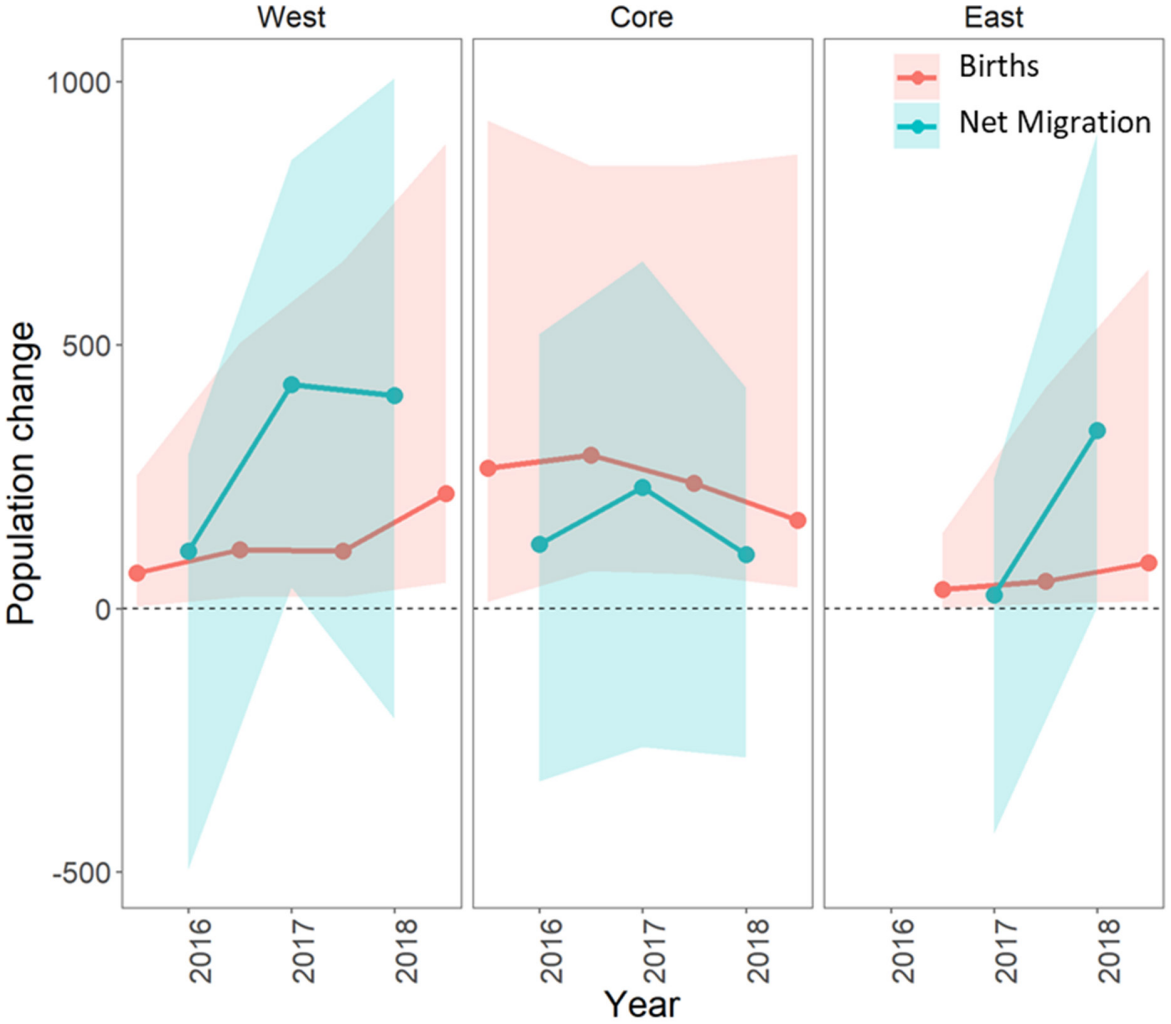
Partitioning adult recruitment each year in each management area into contributions of local births and net migration based on the InfoPM, depicting the posterior distributions (medians and 95% credible intervals) for each parameter. Net migration is summed over the entire year for each location, while ‘births’ represent effective births (or juveniles that are born the previous year, survive to the next birth pulse, and transition to adults) each year. Because the birth pulse is mid‐year, a single tegu cohort spans multiple capture seasons. Thus, we estimate one additional year of birth cohorts compared to estimates for net migration, where year for ‘births’ corresponds to the year of adult transition.

Estimates for adult recruitment via local births and net migration each year from the InfoPM indicate that population growth in each region was driven by a combination of both processes (Figure 3), although the importance of each component was different between management areas and over time. In the Core, point estimates for annual net migration were positive and varied over time (highest in 2017), but the posterior distributions showed some support for zero and negative values (the probabilities that total annual net migration was positive was 0.70 [2016], 0.82 [2017], and 0.72 [2018]). Adult recruitment from local births was higher each year than estimates for net migration (and were also higher in than the West and East regions) and showed a declining trend over time. In the West, posterior estimates for net migration were positive with strong non‐zero and non‐negative support (the probability that total annual net migration was positive was 0.75 [2016], 0.98 [2017], and 0.93 [2018]), and estimates increased between 2016 and 2017 before leveling off in 2018. Adult recruitment from local births increased each year in the West region and reached a similar magnitude as net migration in 2018 and as births in the Core in 2016. In the East, net migration was near zero in 2017, but was positive and significant in 2018 (probability that total annual net migration was positive was 0.40 [2017] and 0.98 [2018]), while births were generally low but increased each year.

**FIGURE 4:**
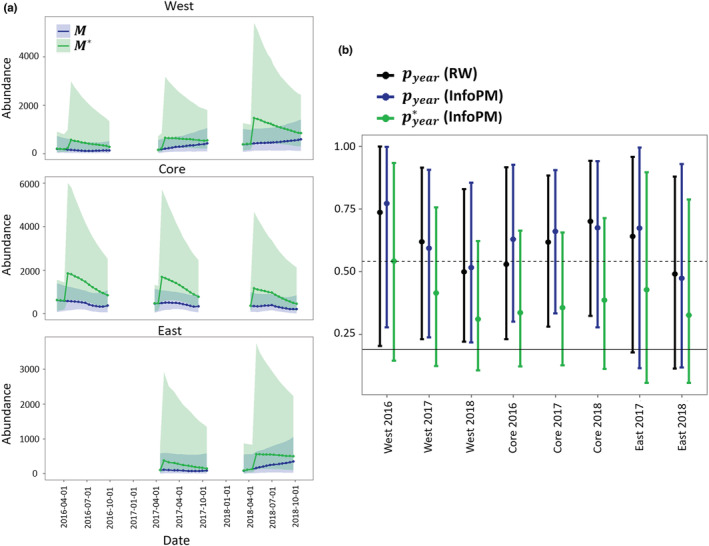
Predictions from the top InfoPM model for the superpopulation abundance and annual capture rates both uncorrected, and corrected, for relative juvenile availability bias. (a) Predictions from the top InfoPM model in each location comparing the superpopulation abundance $\left(M\right)$ uncorrected for hatchling bias and the corrected superpopulation estimate $\left(M^{*}\right)$. The difference in these parameters is driven by juvenile abundance, survival, and relative availability bias. The parameter estimates are most different directly after the birth pulse, and they converge to similar estimates directly before the birth pulse the next year. (b) Yearly effective capture probability in each location and year without and with corrections for juvenile availability bias. Annual capture probabilities $\left(p_{\text{year}}\right)$ account for capture efficiency, trap effort, availability bias, and zero inflation (daily suitability) in each year, whereas $p_{\text{year}}^{*}$ accounts for these processes in addition to juvenile availability and age‐structure throughout the year.

Capture efficiency (i.e., capture probability of a single tegu in a single trap) varied by year and management area (Appendix D, Tables D‐2–D‐6), showing a decreasing trend in the West and East areas over time and an increasing trend in the Core. Estimates for effective period capture rates given daily capture rates, availability, and zero inflation effective capture were similar between models within areas (Figure D‐1). Estimates of annual effective capture probabilities uncorrected for hatchling bias (i.e., effective capture probability of the observable/adult population) for animals that are in a superpopulation the entire year were high in all locations, with point estimates often greater than 0.50. However, correcting for hatchling availability bias and population dynamics led to more conservative estimates (Figure 4), with point estimates less than 0.5 for all but 1 year and management area (2016 in the West management area). Estimates of superpopulation size corrected for hatchling bias (M*) compared to the uncorrected superpopulation estimate (*M*) suggests that estimates for *M* missed a large number of animals immediately after the birth pulse; however, *M* and M* converged almost entirely just prior to the beginning of each birth pulse (Figure 4).

**FIGURE 5:**
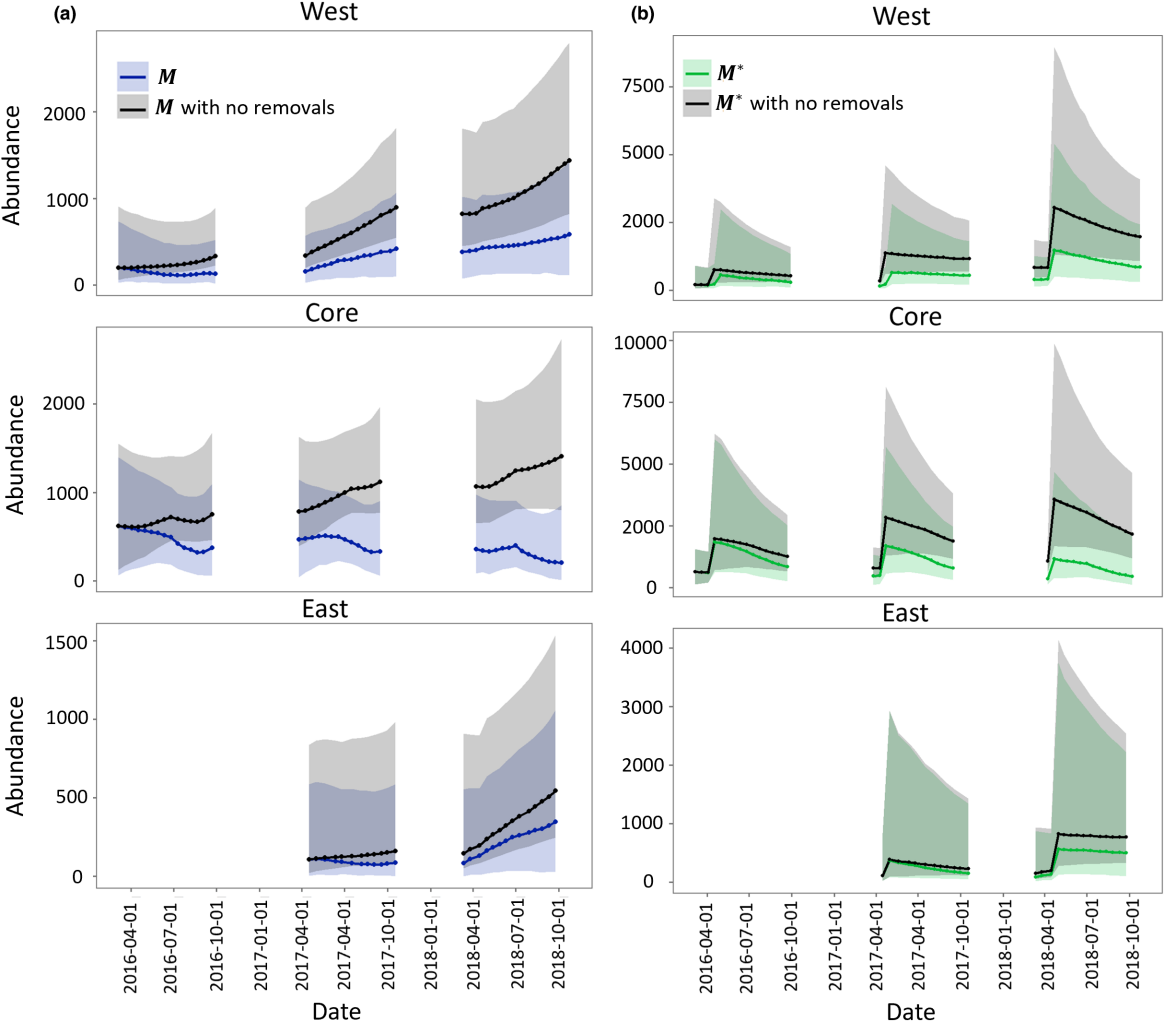
Evaluating the effectiveness of removal actions by comparing estimated trends in uncorrected $\left(M\right)$ and corrected $\left(M^{*}\right)$ superpopulation abundance estimates to predictions from no‐removal scenarios assuming the same dynamics parameters (birth rates, survival, and net migration) in each location and year except for the amount of removed animals. (a) No‐removal scenarios compared to estimates for catchable population $\left(M\right)$ in each location over time. (b) No‐removal scenarios compared to estimates for total abundance corrected for the juvenile age class $\left(M^{*}\right)$ in each location over time.

The yearly birth rates and population growth rates from the InfoPMs were generally more precise than our informative priors based on expert elicitations (Figure D‐2, Tables D‐4–D‐6), which speaks to information in the timeseries of count data updating of these variables. Yearly survival rates for all age classes did not deviate significantly from the priors (Appendix D). Finally, we showed that both *M* and M* (i.e., *M* corrected for hatchling bias) would be significantly higher in each management area by 2018 if removal actions had not taken place (Figure 5). The difference was greatest in the Core area, which had the highest starting population and highest removal effort, but the same pattern was also seen in the West area (and also in the East area to a lesser extent).

The results of our simulation study validated our RW abundance estimator given the expected field conditions in each management area. We found that our estimator was generally unbiased when the removal probabilities and superpopulation sizes were reasonably high, such as in the Core area (Appendix C, Figure C‐1). We found that around 99% of all estimates fell within the 95% credible intervals, around 40% of our estimates were within 20% of the true values when using parameter value for the Core area, and about 20% of estimates were within the same range for the West and East areas. The best performance in terms of bias and accuracy occurred when abundance, removal rates, and availability were high and after the first year of sampling.

## 4 DISCUSSION

In this work, we developed two different open robust‐design removal models to estimate population dynamics, capture probabilities, and time‐varying availability bias from removal sampling time‐series. These models were based on previous research in open unmarked abundance estimators that account for either temporary emigration (Chandler et al., 2011) or population dynamics (Dail & Madsen, 2011; Kéry et al., 2009; Matechou et al., 2016; Zipkin et al., 2014). We demonstrated the value of these models to inform invasive species management by applying them to multi‐year removal trapping data sets of invasive Argentine black and white tegus in three management areas in southern Florida and post‐validated the models via simulation under study conditions. We also built upon previous work in tegu population modeling (Johnson et al., 2017), providing a framework to update vital rate belief distributions with empirical information in the removal time series (Figure D‐2). These models are generally applicable to many removal‐sampling data sets collected in the course of conservation management, such as carcass recovery programs for marine mammals, species relocation programs, hunting time series data, and time‐of‐detection point counts. The models developed here represent important methodological advancements that account for common assumption violations in removal models and in general unmarked abundance estimators (i.e., N‐mixture models, Royle, 2004).

Because we constructed a single likelihood for total removals in the InfoPM, the fit of the two models to the count data are directly comparable (Table D‐7). Furthermore, the RW and InfoPM have parameters in common that allow for direct comparison including the uncorrected superpopulation ($M_{t}$) and mean available abundance $\left(N_{t}\right)$ (Figure 2). These estimates were often similar between the RW and InfoPM models (Figure 2) but were generally more precise for the InfoPMs. Comparing *M* to M* (the superpopulation estimate corrected for relative juvenile availability) highlights the importance of accounting for juvenile availability bias (Figure 4). Furthermore, because *M* can be interpreted as the superpopulation of animals old enough to respond to baited traps, both *M* and M* are meaningful quantities for ecological impacts and conservation management.

Parameter estimates for *M* and *N* from both models indicate that tegu abundance is increasing in the West and East management areas (Figure 2), which is consistent with rapid spread from the initial invasion point followed by local population growth despite management efforts. As of the end of 2018, our analysis suggests that tegu abundance in the West area is likely as large as or larger than in the Core area (Figure 2). This result is troubling given that West area is adjacent to and includes parts of Everglades National Park, an area with unique and threated biodiversity recognized as a UNESCO World Heritage Site. The increased abundance trend in the East area is also troubling given its proximity to threated American crocodiles and other biodiversity at Turkey Point Power Plant. The InfoPM suggests much of the population growth in the West and East areas is driven by net migration (Figure 3, Appendix D, Tables D‐5–D‐6), whereas adult recruitment from local births was most dominant in the Core area. In 2018 in the West, our results suggest that births and net migration contributed similarly. However, because births and immigration processes are confounded without ancillary data, the degree to which they can be reliably separated depends on the quality of prior information included for survival and birth rates. We used informative priors for survival and birth rates derived from expert elicitation, and these results should be interpreted considering the prior information and the model assumptions (e.g., Riecke et al., 2019).

Our analyses suggest that tegu abundance (*M* and M*) either decreased (RW and InfoPM) or at very least stabilized (InfoPM) each year since 2016 in the Core management area (Figure 2), where it would have grown otherwise without removal efforts (Figure 5). Given the model assumptions, our results suggest that tegu populations may be controllable, at least locally, given present trap densities in the Core region and similar levels of local population control may be also achievable in the West and East management areas with increased trapping effort. However, caution is warranted because tegus are prolific breeders (Meshaka et al., 2019) and large‐scale harvest in the native range for the skin trade appears to be sustainable (Fitzgerald, 1994), indicating that local control in the invasive range will be challenging. Furthermore, this conclusion is based on model assumptions and there may be other important processes responsible for this decline that we did not include in these models, such as density dependence, migration from the Core to other management areas, or fluctuation in habitat quality. Furthermore, because net migration was an important source of adult recruitment in the West and East management areas (Figure 3), immigration from outside locations could lead to population growth or maintenance growth despite otherwise local control. We also note that the number of traps and the density of traps based on area alone are not a directly comparable metric between areas given differences in trapping methods and in sizes of the effective capture area based on habitat configuration. Thus, we only model the effect of total effort relative to each area and year. However, ongoing habitat‐selection studies and tegu‐specific habitat maps are under development, which may facilitate efforts for comparable trap‐density measures and changes in the effective capture area over time. Further investigation into effort relationships, especially those relaxing the independence assumption, could help to gain a better understanding of the optimal formulation.

Estimates of availability in each management area supported our a priori expectations of tegu availability throughout the year, being lowest at the start (March) and end (October) of the year (cold season) and highest in the early summer (Figure 2). This trend was most pronounced in the Core area but was also present in the West and East areas (Figure 2). It could be that availability is easier to estimate in the Core area given the higher removal rates, or there are less suitable hibernation sites in the Core area due to generally shallower bedrock compared to the West and East areas, though there is no direct evidence of such a difference. Furthermore, suitable habitat for tegus is more isolated in the Core area compared to the West and East, where the effective trapping regions are adjacent to other upland habitat. Thus, opportunities for temporary migration may be lower in the Core than in the other areas during the middle of the year. Finally, fluctuation in availability in the East and West management areas highlights that availability is a complex process driven by the spatial distribution of tegus, movement and density dependent behaviors, the spatial distribution of food sources, and spatial arrangement of trap effort. Over time, removing tegus also alters the spatial distribution of tegus, and how they respond to these removals will influence availability. Fluctuating availability throughout the year highlights the importance of accounting for this bias when modeling population dynamics, lest changes in availability be misinterpreted as dynamics in superpopulation abundance. While informative covariates can be quite useful for estimating availability (Zhao & Royle, 2019), and a quadratic effect for day of year appears reasonable, additional covariates and random effects are likely needed to sufficiently inform these parameters, so we opted to estimate the availability directly from temporal replication.

By comparing the uncorrected estimates for *M* and annual capture probabilities to those corrected for relative juvenile available and age distribution throughout the year, we can glean the utility of using the InfoPM approach (Figure 4). Our results indicate that the *M* and M* can vary greatly throughout the year, with the largest difference directly after the birth pulse, and the least difference directly before the birth pulse. In fact, directly before the birth pulse the estimates converge, and thus this time of the year may be the best to estimate abundance using the RW approach. In all management areas, the annual removal rate uncorrected for juvenile bias (i.e., the capture rate of adult tegus) suggests that individuals have over a 50% probability of capture by the end of each year if they are present in the superpopulation for the entire year (Figure 4). However, this finding is hard to reconcile with increasing superpopulation abundance in the West and East areas (Figure 2). Furthermore, this estimate is somewhat at odds with the low incidence of recapture rates for telemetered animals in the West management area; however, trap avoidance after first capture may help to explain this difference. After correcting for juvenile bias due to relative juvenile availability and the expected proportion of juveniles in the population throughout the year, the annual removal rates were still generally high but more conservative (Figure 4) and provided more realistic estimates of removal rates given abundance trends. Importantly, these estimates for annual capture probabilities account for every source of bias estimated in these models (detection, availability, temporal suitability, relative juvenile availability, and proportion of juveniles in the population), and heterogeneity in these parameters across primary periods each year. Previous work on tegu population modeling found a removal rate of approximately 20% is needed to stabilize a single age‐structured population when there are no sources of immigration, and when all individuals are equally catchable (Johnson et al., 2017). However, hatchlings are removed at a lower rate than adults, and net migration is present (and often positive) in all populations, thus the true removal rate needed to stabilize or reduce these populations is likely much higher. We found that annual removal rates around 40% in the Core management area appear to have stabilized populations, while in the West and East areas, populations continued to increase despite annual removal rates of around 30%. Technically, when dynamics are maintained by net migration from a separate source, populations could increase or be maintained locally through continued immigration even with removal rates near 1, especially if the source population is large.

Thus, it is possible that the areas adjacent to the effective trapping area in each management area could maintain populations despite local control efforts. This is worth noting because the results from the InfoPM suggest that net migration was a large and significant driver of adult recruitment in the West and East management areas (Figure 3) and also to a lesser extent in the Core area. While the source of these migrants is unclear, private lands bordering the Core and West areas are mostly agricultural areas that are not managed through similar systematic trapping programs due to logistical difficulties. Such adjacent and unmanaged areas may provide critical spatial refugia from removal efforts and could hamper local population control efforts through spillover. Further research into the distribution and abundance of tegus in these locations combined with decision support tools for spatial population management may help to effectively plan the control of such spatially structured populations.

In this work, we used the InfoPM framework to predict an additional ‘no‐removal’ scenario in each management area to evaluate the past effectiveness of each removal program on tegu population dynamics. This model allows managers to address the questions of how removal efforts have affected the population directly, through present mortality, and indirectly, given future survival (i.e., some animals would have died nonetheless) and birth processes (i.e., removed animals cannot contribute to births). We demonstrate through counterfactual analysis that even though the populations have increased in the West and East areas, tegu abundances (both M*and *M*) would be significantly higher without trapping effort, and abundance in the Core area would have grown rather than declined (Figure 4). This framework could also be used to forecast the tegu populations in each management area under different management scenarios assuming average availability and capture conditions throughout a year. Furthermore, the estimates obtained from the InfoPM could be included in formal decision analysis, such as a Markov Decision Process (e.g., Williams, 2009) model where the actions are to decide the amount of trap effort to allocate at each primary period of the year.

The simulation study post‐validated the results of the RW model under similar conditions to those estimated for each management area and year. Our results indicate that we can reliably estimate superpopulation abundance and removal probabilities in all locations with reasonable precision and bias when removal rates and starting population sizes are high. The lowest biases were found in the Core area (median biases each year were within 2%) where removal probabilities and the starting superpopulation was highest. In the West and East areas, where starting *M* and removal probabilities were lower, estimates were less accurate (Appendix C, Figure C‐1), which agrees with previous work on estimating removal model parameters (Davis et al., 2016). For application for invasive species, we view these levels of bias as acceptable, especially given that alternatives (e.g., CPUE) have additional and unmeasured biases including imperfect detection and availability bias (Anderson, 2001). However, when applying this model to systems with lower starting population sizes or lower removal rates, biases would likely be larger, thus it is important to ensure the bias levels are acceptable for the intended application.

These models and applications have several limitations. The first is that we did not account for finer spatial influences in both the capture and population dynamics processes, but rather modeled the superpopulation corresponding to effective capture area in each management area. Future work may extend these models to a spatially explicit version using methods developed for spatially structured removal models (e.g., Kéry & Royle, 2015). A further enhancement would include specifying spatially explicit transition functions for local growth and movement probabilities. For example, recent work in open removal models with spatially explicit movement probabilities (Link, Schofield, et al., 2018) appears promising if extended to include availability bias or age‐structure. A spatial approach would also allow for more precise estimates of the relationship between capture probabilities and trap effort and could incorporate spatial covariates; however, the degree of temporary emigration bias due to partial overlap of home ranges will likely be higher which could make estimation challenging. A second limitation is that we used non‐age‐structured removal data in our InfoPM framework. Recent methods (Meshaka et al., 2019) to age tegus given size and time of year, or skeleton chronology, could be used in future work to explicitly include age‐structured removal data into the likelihood (e.g., Zipkin et al., 2014), but given the generally low incidence of juvenile captures, prior information may still be required. Because we make a prediction for the expected captures of adults and juveniles already with our combined likelihood approach (Equation 16), extending out InfoPM to age‐structured removals would only require separating the first half the of the combined equation the likelihood for adult removals, and the second half of the equation as: the likelihood for juvenile removals. Such an approach would also provide additional empirical information to estimate the relative juvenile availability distribution, vital rates, and abundance. A third limitation is that we did not incorporate density dependence, which becomes more important as populations continue to grow larger over time. A fourth limitation is the assumption that adult and juvenile survival are constant throughout the year since these are likely to vary due to seasonal influences. However, with the current formulation of net migration each period, a model with time varying survival would be overparameterized. Net migration is informed by the difference in population dynamics each period that is not explained by birth rates and constant survival rates, thus the net migration process absorbs differences in time varying survival, and it may be better termed “apparent net migration”.

Another key assumption of our model was that data are collected using a robust‐design framework; however, our data were collected throughout the entire year and so primary periods and temporal dynamics were approximated. We used a time period of 2‐weeks to designate primary periods, was long enough period to achieve high enough total removal rates to estimate the models (e.g., Davis et al., 2016), and short enough to reasonable assume population closure other than temporary emigration. Future work may evaluate the optimal blocking structure for estimating abundance given capture rates and collection periods. Finally, we did not account for differences in multiple types of removal methods (e.g., trap types); rather, we estimate the average capture efficiency of traps based in each location each year. Future work could extend this framework to estimate the capture efficiency of traps separately or investigate different relationships between effort and capture probabilities.

In this work, we developed open removal abundance estimators to overcome common violations of assumptions in removal time series, which arise due to population dynamics and time‐varying availability bias. Such models provide an important monitoring tools for invasive species management programs to evaluate past effort and efficiently plan for future management efforts. We demonstrated the utility of these models for the Argentine black and white tegus in South Florida and provide important insights into the past and future effectiveness of trapping programs. We importantly provided the first empirical evidence that populations may be controllable locally given present levels of trap densities in the Core management area, which provides an opportunity for successful control of invasive tegu populations. However, we highlight continued growth in the West and East areas, possible source locations in nearby spatial refugia, and continued potential for spread to ecologically sensitive areas as remaining challenges for management. Finally, the models we developed here can be applied to other removal monitoring programs to estimate population dynamics and inform conservation management while accounting for capture probabilities and availability bias.

## AUTHOR CONTRIBUTIONS

**Bradley Udell:** Conceptualization (lead); data curation (equal); formal analysis (lead); investigation (lead); methodology (lead); project administration (equal); validation (lead); visualization (lead); writing – original draft (lead); writing – review and editing (lead). **Julien Martin:** Conceptualization (supporting); formal analysis (equal); funding acquisition (equal); investigation (equal); methodology (equal); project administration (equal); resources (equal); supervision (equal); validation (supporting); visualization (supporting); writing – original draft (supporting); writing – review and editing (supporting). **Christina Romagosa:** Conceptualization (supporting); data curation (supporting); formal analysis (supporting); funding acquisition (equal); investigation (supporting); methodology (supporting); project administration (equal); resources (equal); supervision (equal); validation (supporting); visualization (supporting); writing – original draft (supporting); writing – review and editing (supporting). **Hardin Waddle:** Conceptualization (supporting); data curation (equal); formal analysis (supporting); funding acquisition (equal); investigation (supporting); methodology (supporting); project administration (equal); resources (equal); validation (supporting); visualization (supporting); writing – original draft (supporting); writing – review and editing (supporting). **Fred Johnson:** Conceptualization (supporting); formal analysis (supporting); funding acquisition (equal); investigation (supporting); methodology (supporting); project administration (equal); resources (equal); supervision (equal); validation (supporting); visualization (supporting); writing – original draft (supporting); writing – review and editing (supporting). **Bryan Falk:** Conceptualization (supporting); data curation (equal); funding acquisition (equal); investigation (supporting); methodology (equal); project administration (equal); resources (equal); validation (supporting); writing – original draft (supporting); writing – review and editing (supporting). **Amy Yackel Adams:** Conceptualization (supporting); data curation (equal); formal analysis (supporting); funding acquisition (equal); investigation (supporting); methodology (equal); project administration (equal); resources (equal); validation (supporting); writing – original draft (supporting); writing – review and editing (supporting). **Sarah Funck:** Conceptualization (supporting); data curation (equal); funding acquisition (equal); project administration (equal); resources (equal); writing – original draft (supporting); writing – review and editing (supporting). **Jennifer Ketterlin:** Conceptualization (supporting); data curation (equal); methodology (supporting); project administration (equal); resources (equal); writing – original draft (supporting); writing – review and editing (supporting). **Eric Suarez:** Conceptualization (supporting); data curation (equal); methodology (supporting); project administration (supporting); resources (supporting); writing – original draft (supporting); writing – review and editing (supporting). **Frank Mazzotti:** Conceptualization (supporting); data curation (equal); methodology (equal); project administration (equal); resources (equal); writing – original draft (supporting); writing – review and editing (supporting).

## Supporting information


Appendix S1

## Data Availability

Data and computer code (R and JAGS) are archived at: Waddle H., Udell B., Martin J., Romagosa C., Johnson F., Falk B., Yackel A. A., Funck S., Ketterlin J., Suarez E., and Mazzotti F. (2022) Data for analysis of open removal models with temporary emigration and population dynamics to inform invasive animal management. U.S. Geological Survey data release, https://doi.org/10.5066/P9NXHC0V.
