# Corrigendum

**DOI:** 10.1002/ece3.9315

**Published:** 2022-09-12

In the recent article by Udell et al. (2022), Dr. Christina Romagosa was put as the final author of the article. The correct order of authors is shown below:

Bradley Udell^1^ | Julien Martin^2,3^ | Hardin Waddle^2^ | Fred Johnson^4^ | Bryan Falk^5,6^ | Amy Yackel Adams^5^ | Sarah Funck^7^ | Jennifer Ketterlin^6^ | Eric Suarez^7^ | Frank Mazzotti^8^ | Christina Romagosa^1^

Lambda_tj (lambda with subscript t and j) in equation 2 should actually just be Lambda_t (lambda with subscript t):

(2) $$y_{\mathrm{tj}}\sim \text{Poisson}\left(\phi_{t}*\pi_{\mathrm{tj}}*\lambda_{t}\right)$$

We apologize for these errors.
