# Supplementary material for: Open removal models with temporary emigration and population dynamics to inform invasive animal management
Source: Ecol Evol. 2022 Aug 17;12(8):e9173. doi: 10.1002/ece3.9173 (PMC9382647; doi:10.1002/ece3.9173)
Supplement: Supplementary file 1 — Appendix S1 [file ECE3-12-e9173-s001.docx]

Supporting Information. Bradley J. Udell , Julien Martin , Christina M. Romagosa , J. Hardin Waddle , Fred Johnson , Bryan Falk , Amy A. Yackel Adams , Sarah Funk , Jennifer Ketterlin , Eric Suarez and Frank Mazzotti. Open removal models with temporary emigration and population dynamics to inform invasive animal management. Ecology and Evolution.

**Appendix A: Study area and removal trapping methodology**

Tegu removal data were obtained from agency partners with systematic removal trapping programs in three management areas. Tegus are managed in collaboration by Everglades National Park (ENP) and United States Geological Survey (USGS) (ENP/USGS) in the West management area, by University of Florida partners in the Core management area, and by Florida Fish and Wildlife Commission (FWC) in the East management area (Figure 1). The frequency of trapping, trap effort, and the composition of habitat and traps types varied between management areas and years. In general, traps were deployed between February-September, baited with chicken eggs, and checked daily. The mean number of traps, mean number of tegus caught per day, and the catch per unit effort (CPUE) for each period are reported for each management area (Figure A-1)

**
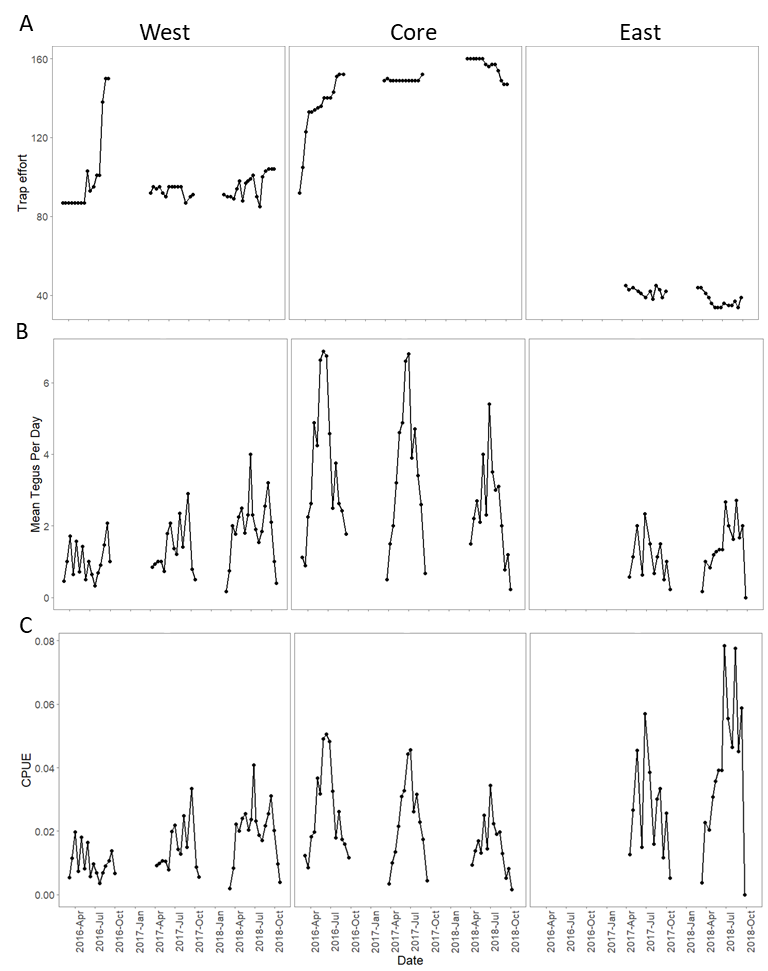
**

Figure A-1. Trap effort, mean animals caught per day, and mean CPUE for each primary period and management area. A) Trap effort. B) Mean animals caught per day. C) Mean CPUE per primary period.

West Management Area

Area Description

The ENP/USGS tegu study area is adjacent to the eastern boundary of and within Everglades National Park. Agricultural development is within, to the east of, and to north of the study area. The study area also includes parts of the Southern Glades Wildlife Environmental Area, the Frog Pond Wildlife Management Area, and the Rocky Glades Public Small Game Hunting Area, and these areas are dominated by freshwater marsh and marl prairie interspersed with hardwood hammocks and tree islands, as well as canals, levees, berms and other structures that are used to manage the hydrology of the area. The dominant vegetation of the marshes and prairies include sawgrass (*Cladium jamaicense*) and muhly grass (*Muhlenbergia capillaris*). Vegetation along elevated levees, where trap transects are located, includes native species such as poisonwood (*Metopium toxiferum*), strangler fig (*Ficus spp*.) willow (*Salix spp.)* and saltbush (*Baccharis halimifolia*), and exotic species such as Brazilian pepper (*Schinus terabinthafolius*), lead tree *(Leucaena leucocephala*), and various species of cane grass.

Trapping Methods

ENP/USGS conducted annual tegu live-trapping between February and October with temporary trap closures during hurricanes, deploying between 99-150 traps at a time at 123 trap locations (i.e., some locations had more than one trap). Traps were deployed along 10 transect lines (Table A-1) of varying length with traps spaced at 500-meter intervals. Transects were labeled using the section of the canal and a cardinal direction to indicate whether transects were on the east or west side of the canal or north or south of a landmark (Table A-1). Traps were baited with one chicken egg in a protective bait cage. In some instances, alternative bait of tuna, cat food, or attractant oil was trialed. Multiple traps types were deployed and mostly consisted of modified Havahart and Tomahawk traps (model number and dimensions [L × W × H]), of three sizes large (1089; 81 × 27 × 32), medium (1088; 61 × 20 × 20), and small (1025B; 46 × 13 × 13) to enhance capture of a size range of tegu lizards. 1025B model traps were modified in four ways to capture juvenile tegus: increased sensitivity of the trip plate to ≤10g, closed one of the two doors, installed wooden dowels to block gaps between doors, and attached 0.6-cm hardware cloth to trap exterior to minimize gap size. In 2016 (July to October), two trap types (1025B and 1089 or 1088) were place at selected trap locations. In 2017-2018, traps were alternated at trap locations so that in most cases, every third trap was a different size. All traps were placed on the ground in a shaded location and checked daily in 2016-17 and primarily weekdays in 2018. If adequate shade was not present, traps were manually covered with vegetation. All tegus caught were removed, euthanized, and necropsied immediately after euthanasia. Any use of trade, firm, or product names is for descriptive purposes only and does not imply endorsement by the U.S. government.

Table A-1. ENP/USGS transects name and description for tegu trapping adjacent to and within Everglades National Park, 2016-2108.

| Transect | Description |
| --- | --- |
| 424SW | SW 424^th^ St., west of the C-110 canal |
| AERO | Aerojet Road |
| C-110E | The east side of the C-110 canal, south of the old SFWMD structure |
| C-111EE | The east side of the C-111E canal |
| C-111EW | The west side of the C-111E canal |
| C-111N | The C-111 canal north of SR 9336 |
| C-111SE | The east side of the C-111 canal south of SR 9336 but north of the intersection with the C-111E canal |
| C-111SW | The west side of the C-111 canal south of SR 9336 and the C-111 canal south of the intersection with the C-111E canal |
| EVER | Everglades National Park |
| L-31WS | The L-31W canal south of SR 9336 |

Core Management Area

Area Description

The Core management area is the area surrounding the first location of establishment in the Southern Glades Wildlife Environmental area near the Miami-Dade Juvenile Residential Facility. The habitat consists of a disturbed short-hydroperiod marl prairie composed of wet prairies, tree islands, sawgrass (*Cladium jamaicense*), cocoplum (*Chrysobalanus icaco*), as well as tropical hammock plant communities (Pernas et al. 2012). Tegu traps were primarily arrayed along high elevation, manmade linear features including roads, levies, and canals (Figure A-1) along which tegus are dispersing to the West and East management areas. Given this landscape of linear features interspersed among mostly uninhabitable matrix of marsh habitat, effective trap densities per unit area are likely higher here than in the West management area where high elevation habitat is more widely distributed. Furthermore, given the relative scarcity of high elevation habitat and suitable limestone for burrowing, this management area may show different availability patterns than other management areas throughout the year.

Trapping Methods

UF deployed between 92-152 traps in 2016, 150 traps in 2017, and up to 160 traps in 2019 in Southern Glades Wildlife and Environmental Area and the C-111 basin of Miami-Dade County. Traps were deployed along the C-110 canal (North-South), SW 424^th^ Street (East-West), and along the southern portion of the C-111 canal and baited with chicken eggs. Starting February each year, traps were opened on Monday, checked daily, and closed each Friday with the exceptions of University and federal holidays or force majeure. UF used 45 Havahart model 1079 traps and 45 Tomahawk model S50 traps arranged in pairs of traps (one of each trap type). The remaining traps were deployed in drift fence arrays or as stand-alone traps without a pair. For drift fences, UF placed one to five traps along each drift fence array, depending on fence length and topography of surrounding habitat. Drift fences were constructed out of aluminum flashing held in place by rebar or along pre-existing concrete walls. Varying combinations of traps were deployed at each drift fence array consisting mostly of Tomahawk traps interspersed with a few Havahart and minnow traps.

East Management Area

Area Description

The habitat in this management area consists of one paved road (Card Sound Road) and one un-paved, gravel road (Tallahassee Road). Both of these roads are located in Florida City, Florida. Tallahassee Road has low vehicle traffic (mostly service vehicles) while Card Sound Road has much higher vehicle traffic (road leads from Florida City to the Keys). Traps along both roads were placed along the Department of Transportation (DOT) right-of-way that runs parallel to different sets of canal/marsh containing a mixture of trees and shrubs. Habitat types adjacent to roads include: urban, marsh, palmetto prairie, prairies and bogs, cropland, sand and gravel pits, quarry ponds, freshwater forested wetlands, and nurseries. This habitat consists of both native (e.g., cabbage palm [*Sabal palmetto*]) and exotic mixed hardwood trees and shrubs line the area between the roads and canal/marsh.

Trapping methods

In the 2017 tegu season, traps were set along Tallahassee road and Card Sound Road (25 traps per road, 50 total) at ~500 meters between each trap. In the 2018 season, the trap lines were slightly reconfigured to better target tegus along Card Sound road, putting 35 traps (~370 meters apart) on Card Sound and 15 on Tallahassee roads (~670 meters apart). A combination of Havahart and Tomahawk traps (mostly Havahart) were used. Traps typically were checked 4 days a week. Traps were deployed from February through October both years. Traps were placed within 10 ft of the vegetation line to provide cover. Leaf litter was put in the trap to cover bottom to mimic ground cover. All traps were baited with 1 raw chicken egg. Sometimes traps were damaged or stolen and were replaced as soon as possible. In 2018, approximately 60% of traps were also wrapped with hardware cloth.

**Appendix B: Estimating age-structured vital rates and informative priors for the InfoPM removal model for Argentine black and white tegus**

We built upon previous work which estimated belief distributions for age structure vital rates for Argentine black and white tegus in Florida from expert elicitation (Johnson et al. 2017). Belief distributions for each vital rate were estimated using a 3-point expert elicitation process with 11 experts. They constructed stochastic Leslie matrix population models using correlated samples from these belief distributions, one Leslie matrix per sample, which were then used to derive belief distributions for scalar growth rates and stable age distributions.

Similar to Johnson et al. 2017, we assumed that tegu populations were structured with 4 age classes: hatchlings/juveniles, 1 yr., 2 yr., and ≥3 yr. individuals, where only the ≥3 yr. age class breeds (Johnson et al 2017). Johnson et al. 2017 use a post-birth Leslie matrix model; however, we reparametrized this model as a pre-birth Leslie matrix model, which has advantages when converting to a 2-age class approximation (implicit juveniles and an explicit averaged adult class). As juveniles are implicit in pre-birth formulations, juvenile survival is included in the effective fecundity term, and only age classes 2-4 are tracked. The resulting 4-age class pre-birth Leslie matrix is as follows:

| $\boldsymbol{A}=\left[ \begin{matrix} 0 & 0 & Fs_{h} \\ s_{1} & 0 & 0 \\ 0 & s_{2} & s_{3} \end{matrix} \right]$ | (B-1) |
| --- | --- |

Thus, the effective birth rate $Fs_{h}$ in this model corresponds to the rate of animal’s transitioning to 1 year old individuals a year after the birth pulse, based abundance of individuals one year and older at the time of the birth pulse. Assuming a sex ratio of 0.5, the birth rate in a post-birth Leslie matrix model is calculated as 0.5*clutch size*egg survival, and for a pre-birth model, is calculated as 0.5*clutch size*egg survival*hatchling survival.

Table B-1. Medians and 95% credible intervals for age-specific vital rates derived from expert elicitation (Johnson et al. 2017).

| Parameter | Estimate |
| --- | --- |
| Survival-Hatchling | 0.211 (0.082 - 0.665) |
| Survival-1yr | 0.502 (0.268 - 0.740) |
| Survival-2yr | 0.631 (0.382 - 0.811) |
| Survival-3yr+ | 0.762 (0.511 - 0.898) |
| Clutch size | 25.803 (20.146 - 33.883) |
| Egg Survival | 0.894 (0.006 - 0.999) |

An eigen-analysis of the projection matrix $\boldsymbol{A}$ can be used to derive important population level quantities, such as the scalarized population growth rate and the stable age distribution. For example, using the point estimates of the age structured vital rate distributions led to a scalar population growth rate of 1.309847, and a stable age distribution of 1yr, 2yr, and 3yr+ individuals of 0.54, 0.22, and 0.24, and reproductive values of 1, 2.5, and 5.19. Reproductive values are the number of offspring that an individual is expected to produce over its remaining life space, which is dependent on the amount of future reproduction, the probability of surviving to realize it, and the time required for the offspring to be produced (Caswell 2001). Furthermore, the stable age distribution can be used to take a weighted average of survival across all age classes 1yr and up to yield the equivalent scalarized population-level survival rate.

Johnson et al. (2017) fit distributions to each variable (beta distributions for parameters between 0 and 1, and lognormal distributions for parameters between 0 and infinity) and simulated 50,000 correlated draws from these belief distribution using a Cholesky decomposition. We did the same and used these 50,000 sets of correlated samples to construct 50,000 projection matrices, which we used to derive samples of the resulting belief distribution for the scalar population growth rate (often referred to as $\lambda$ but for which we use $1+r_{d}$ to avoid confusion with the expected superpopulation abundance $\lambda_{t}$) and stable age distributions for each age class.

The stable age distribution (SAD) was derived for each Leslie matrix using an eigen-analysis (popbio R package). While the SAD has a stochastic distribution, it is based on equilibrium assumptions. However, there is a significant likelihood of transience due to age structure in the early stages of invasion (Johnson et al. 2017). Thus, the priors derived using the SAD distribution will likely be more certain than if transience is considered. In applied Bayesian models, it is sometimes desirable to decrease the precision/information in informative priors to make priors “weakly informative”, with the idea that the loss in precision from making the prior a bit too weak is less serious than the gain in robustness from including additional parts of parameter space that might be relevant. Given that SAD is structurally similar to a multinomial probability vector with probabilities summing to one, we simulated an additional stochastic noise for each SAD from a Dirichlet distribution, with probabilities equal to SAD*30, which results in the same means but a higher variance compared to the SAD distribution based directly on the eigen-analysis.

| $SA{D_{rand}}_{k}=Dirchlet(\mathrm{SAD}_{k}*30)$ | (B-2) |
| --- | --- |

We chose the value 30 after determining this induced additional noise at realistic levels, where higher values lead to less error, and lower values lead to more error.

We then derived the scalarized birth rate and survival probability for the averaged adult (year 1 and older) age class using the following procedure. First, we defined a 50,0000 starting population vectors $N_{0_{k}}$ based on the samples of the stochastic age distribution by multiplying the $SA{D_{rand}}_{k}$by 100, which yields all whole numbers for the initial population vector with the same age distribution.

| $N_{0_{k}}=SA{D_{rand}}_{k}*100$ | (B-3) |
| --- | --- |

We then used matrix algebra to calculate ${N_{k}}_{+}$the population vector at time t+1 given the initial abundance vector ${N_{0}}_{k}$ and Leslie projection matrix $A_{k}$ for each simulation k:

| ${N_{k}}_{+}=A_{k}N_{0_{k}}$ | (B-4) |
| --- | --- |

Next, we calculated the birth rate given the number of 1yr old individuals at time t+1, and the total population size a time t, noting that this formulation accounts for both the age distribution as the vital rates:

| $b_{k}=\frac{N_{k}^{+}\left[ 1 \right]}{\sum N_{0_{k}}}$ | (B-5) |
| --- | --- |

The scalar survival rate of the averaged “adult” age class can be calculated as the average across age classes weighted by the stable age distribution of each.

| $s_{vector_{k}}=\left[ s_{1_{k}}, {s_{2}}_{k}, s_{3_{k}} \right]$ | (B-6) |
| --- | --- |
| $\hat{s_{k}}=\sum(SA{D_{rand}}_{k}*s_{vector_{k}})$ | (B-7) |

As we also have simulations from the belief distribution for the hatchlings survival $s_{h_{k}}$, we have all the necessary pieces of prior information to inform the dynamics in a 2-age class InfoPM. Similar to Johnson et al. 2017, we fitted a beta distribution to adult survival and used this as the informative prior for annual adult survival.


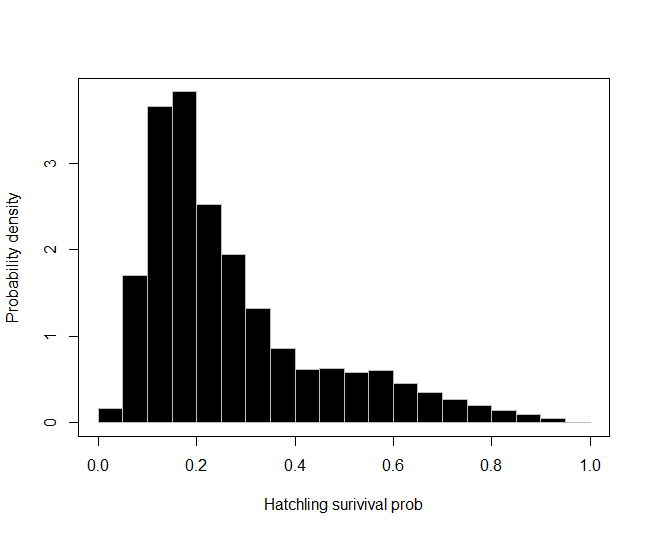


Figure B-1. The belief distribution for hatchling/juvenile survival estimated from Johnson et al. (2017). The mean was 0.273, median was 0.213, the standard deviation was 0.182, and the 95% CRI was 0.067 – 0.750)


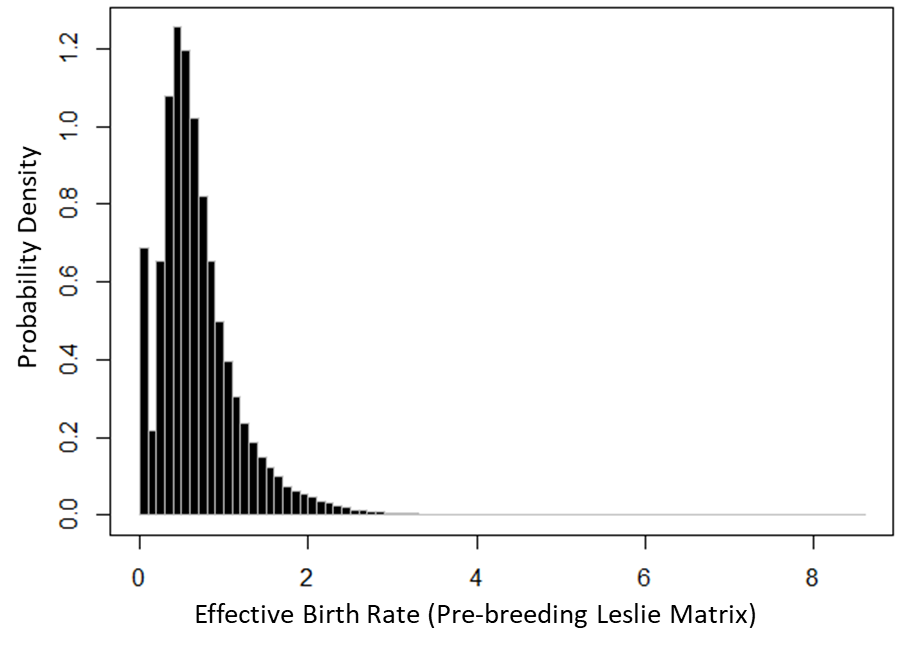


Figure B-2. The belief distribution for the effective birthrate (including hatching/juvenile survival, i.e., the rate of adults producing age 1 individuals) resulting from the stochastic, pre-breeding Leslie matrices. The mean was 0.693, median was 0.593, the standard deviation was 0.489, and the 95% CRI was 1.59e-5 – 1.970.


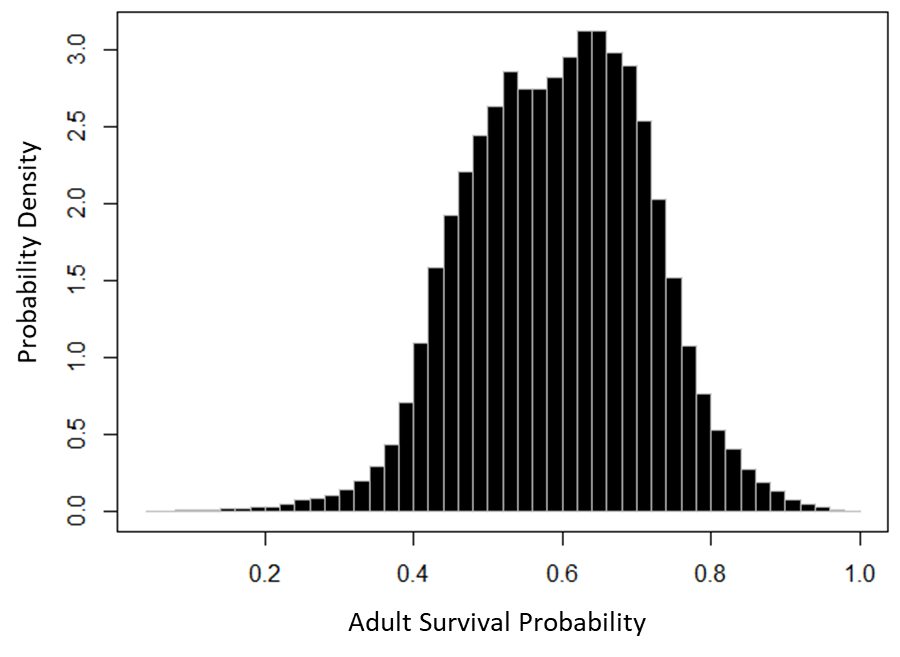


Figure B-3. The belief distribution for the adult survival resulting estimated from Johnson et al. (2017). The mean was 0.593, median was 0.597, the standard deviation was 0.119, and the 95% CRI was 0.366 – 0.817.

**Incorporating Correlations in Hatchling Survival and Effective Birth Rates**

A final consideration is the strong degree of correlation between juvenile (hatchling) survival, and the birth rate distribution, which is especially important to consider. We made a prediction of the juvenile age class based on the quotient of the effective birth and juvenile survival. In other words, the expected size of the hatchling cohort directly after the birth pulse is $\frac{B_{y}}{S_{h}}$, after 1 year is $B_{y}$, and after some number of primary periods since the birth pulse is $\frac{B_{y}}{\left( S_{h}^{\frac{Total Period- nperiod}{Total Period}} \right)}$. Thus, the effective birth rate is not independent of the juvenile survival, and correlations already exist between survival and birth rates due to correlations in the expert estimates, thus we accounted for the correlation structure in these parameters.


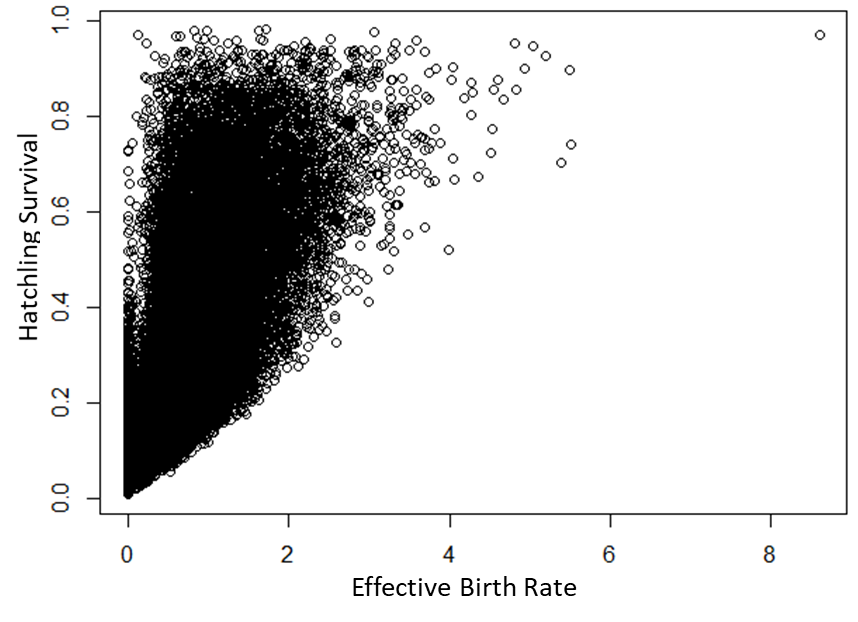


Figure B-4. Scatter plot of the correlated samples for effective birth rates B and the hatchling/juvenile survival. The correlation between samples was $\rho=0.$70.

We fitted a multivariate normal distribution to these parameters on appropriately transformed scales, using a log transformation for the birth rate and a logit transformation for the juvenile survival. We estimated the log-mean and log-variance of the birth rate using a lognormal distribution, and the logit-mean and logit-variance for hatchling survival probability based on the empirical mean and variance of the 50,000 transformed samples. We then estimated the correlation $\rho$ between parameter samples and constructed a covariance matrix based on variance and correlations.

| $\left[ \mathrm{logit} \left( {s_{juv}}_{y} \right), log\left( b_{y} \right) \right] \sim MVN\left( \left[ mu_{trans} \right], \Sigma_{trans} \right])$ | (B-8) |
| --- | --- |
| $\left[ mu_{trans} \right]=\left( \mathrm{logit} \left( \hat{{s_{juv}}_{y}} \right), log\left( \hat{b}_{y} \right) \right)$ | (B-9) |
| $\Sigma_{trans}=\left[ \begin{matrix} sd_{logit\left( Sh \right)}^{2} & \rho*sd_{logit\left( Sh \right)}*sd_{log\left( b \right)} \\ \rho*sd_{logit\left( Sh \right)}*sd_{log\left( b \right)} & sd_{log\left( b \right)}^{2} \end{matrix} \right]$ | (B-10) |

This resulted in an informative multivariate normal prior of juvenile survival and the birth rate on appropriately transformed scales. While we estimated and specified a prior correlation value $\rho$ = 0.7 for this distribution, we found these results were not sensitive to this prior correlation value, and prior values near zero and one yielded similar results.

This resulted in the following hyper-parameters for the informative priors. For adult survival, we used a beta distribution shape with parameters equal to 9.897216 and 6.755582. For hatchling survival and the effective birth rate distribution, we specified a multivariate normal distribution on the transformed parameter scale (a log link function for effective birth rates, and a logit link function for hatchling survival). We used a mean of 0.4045 and a standard deviation of 0.54 for the hatchling survival on the logit scale, and a mean of -0.5229491 and a standard deviation on the log scale of 0.6129441. Finally, we included a correlation of $\rho=$0.68 on the transformed scale.


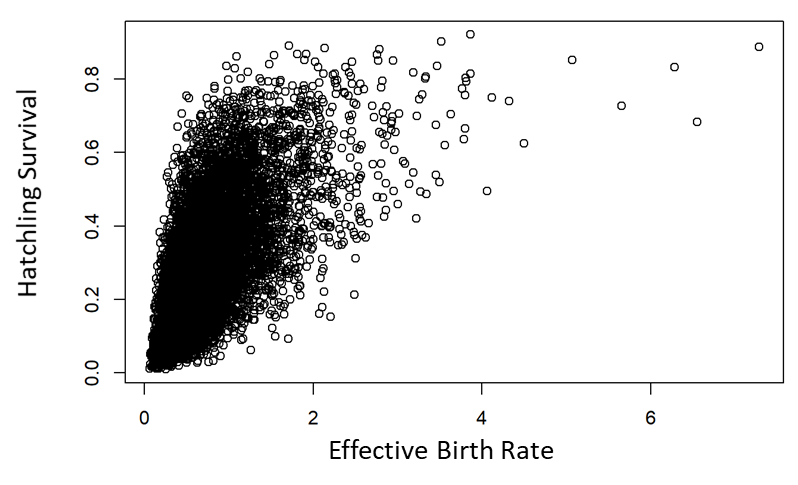


Figure B-5. Scatter plot of the samples from 10,000 samples from the multivariate normal correlated informative priors for effective birth rates and after back-transforming parameters. The correlation between samples was $\rho=$ 0.70.

**Appendix C:** **Post-validating the random walk model through simulation study**

We post-validated the application of our random walk model under study conditions for each year and management area using a simulation study (e.g., Davis et al. 2016, Zipkin et al. 2016). We simulated multiple data sets from the full generative model with temporary emigration and random walk dynamics given the parameter estimates for starting abundance, daily detection, temporal suitability, and availability bias in for best supported models. We then calculated three metrics to quantify bias and accuracy. The first metric is the number of estimates which fall within the 95% credible interval, where at least 95% of runs should fall within this range. The second and third metrics are based on summaries of the parameter bias distribution, where bias is defined as: $\frac{\left( \hat{M}-M_{true} \right)}{M_{true}}$, using the medians as the point estimates. For expected bias, we calculated the median of this distribution as the point estimate, and for accuracy we quantified the proportion of estimates that fell within 20% of $\hat{M}$ (i.e., the proportion of runs with an absolute value of bias less than 0.2. Finally, we also calculated the absolute bias $\left( \hat{M}-M_{true} \right)$ distributions as a comparison for areas and years with low starting abundances (e.g., the West and East management areas in the first year of sampling).

We simulated the full generative model in each location:

| $M_{t}\sim Poisson\left( \lambda_{t} \right)$ | (C-1) |
| --- | --- |
| $N_{tj} \sim Binomial\left( M_{t}, \phi_{t} \right)$ | (C-2) |
| $z_{tj} \sim Bernoulli\left( z_{prob} \right)$ | (C-3) |
| $y_{tj} \sim Poisson\left( N_{tj}*z_{tj}*\pi_{tj} \right)$ | (C-4) |
| $M_{t+1}=Poisson\left( M_{t}-R_{t}+\Delta_{t} \right)$ | (C-5) |
| $\Delta_{t} \sim Normal\left( \mu_{trend},sd_{trend} \right)$ | (C-6) |

In each location, we simulated dynamics and removals across the capture timeframe of each (3 years in the Core and West management areas, 2 years in the East areas). Due to time constraints, we ran adjusted MCMC settings in each location to values that converged in a reasonable time frame. In the Core and West management areas, we used 80,000 iterations with thin rate of 2 each after an adaptation period of 20,000 and a burn in of 40,000; For models in the East region we used 150,000 iterations with a thin rate of 2 each after an adaptation period of 50,000 and a burn in of 100,000. Using these settings, we simulated 130 data sets using for each set of conditions and discarded runs that had not reached convergence, for a total of 120 simulations each for the East and West area scenarios and 114 simulations for the Core area scenario. We then calculated the bias, accuracy, and the percent of estimates within the 95% credible intervals for each location and year. Values for $M_{true}$ ranged between 13-1002 in the Core area scenario (starting M = 800 in 2016), between 41-636 in for the West scenario (starting M = 225 in 2016), and between 50-442 in the East scenario (starting M = 300 in 2017). We show the results summarized below.


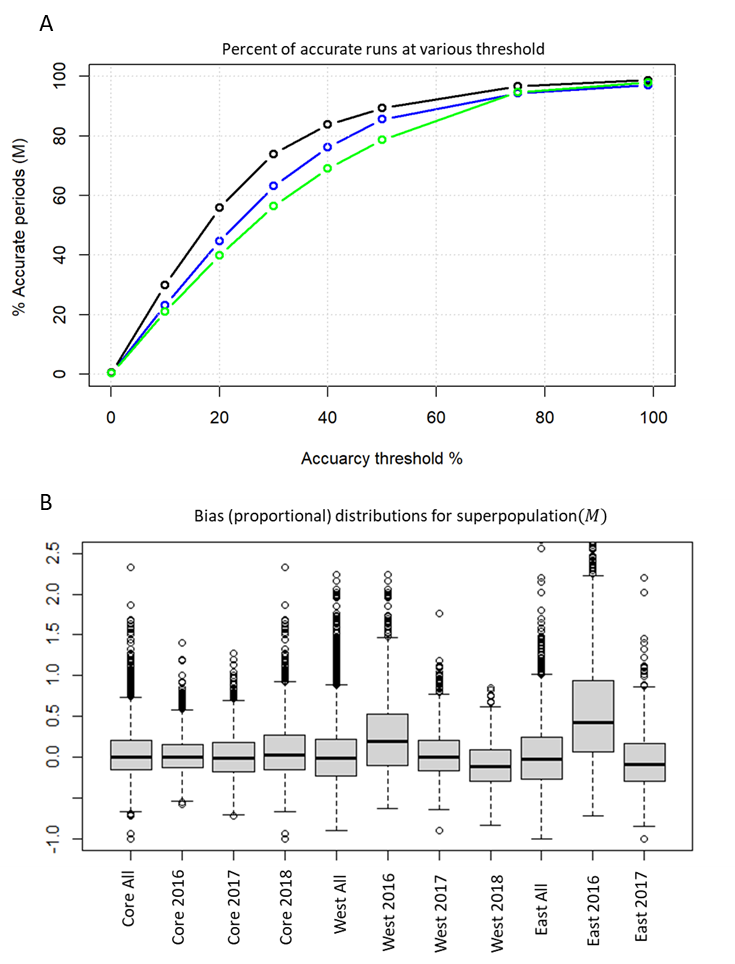


Figure C-1. Post-validating the random walk removal model with temporary emigration for each location using simulation. The estimates for $M_{t}$in all regions led to over 99% of estimates fell within the 95% credible intervals. A: Evaluation of estimator accuracy, measured as the percent of all estimates of $M_{t}$ across all years for each location which fall within the specified accuracy thresholds of the true values. Around 60% of estimates from the Core (black) were within 20% of the true value, while around 40% of the estimates from the West (blue) and East (green) regions were accurate to the same extent. B: Proportional bias distributions for $M$ for each region, overall and summarized by year. Note, the y-axis was truncated above 2.5 for visualization purposes, which does not display some extreme values for the East region in 2016 (values as high as 10 were driven by a small starting population size). Estimates were generally unbiased for all locations and years, except for the West region and East region in first year of sampling where starting abundance was low. Biases were less in subsequent years. The lowest biases were observed for all years in the Core region, which were near zero (e.g., median bias = 0.006 across all years).

**
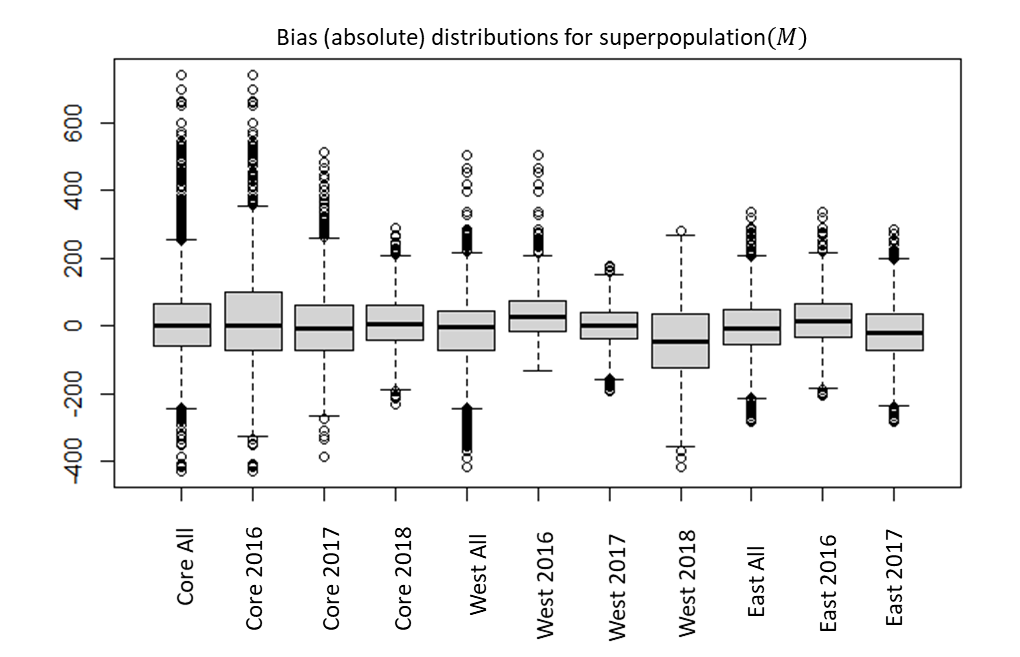
**

Figure C-2. Absolute (i.e., total number of animals) bias distributions for $M$ for each management area (overall and each year). Absolute biases in the East and West regions in 2016 are more similar to the overall mean for each region than their corresponding proportional biases (which were large due to small starting population sizes).

**Appendix D:** **supplemental results from tegu abundance removal models**

Model Estimates

RW Models

West management area: trend by year

Table D-1. Additional parameter estimates for the random walk model in the West management area. Parameters include: mean trends by year$\left( \mu\right)$, standard deviation of the random walk by year $\left( sd \right),$capture efficiency by year $\left( \theta\right)$, and the temporal suitability parameter from the zero inflation process by year $\left( z_{prob} \right).$ For parameter definitions, see Table 2-1.

| Parameter | Mean | Sd | LCI | Median | UCI |
| --- | --- | --- | --- | --- | --- |
| $\mu_{1}$ | 8.37269 | 9.97859 | -17.95162 | 10.44878 | 22.10495 |
| $\mu_{2}$ | 35.28869 | 13.40498 | 10.57035 | 33.86145 | 65.53433 |
| $\mu_{3}$ | 29.52098 | 15.85712 | 0.45008 | 28.12886 | 64.68490 |
| $\theta_{1}$ | 0.00019 | 0.00015 | 0.00002 | 0.00015 | 0.00056 |
| $\theta_{2}$ | 0.00012 | 0.00006 | 0.00003 | 0.00011 | 0.00027 |
| $\theta_{3}$ | 0.00011 | 0.00006 | 0.00003 | 0.00009 | 0.00025 |
| $sd_{1}$ | 7.73359 | 11.21436 | 0.19303 | 4.31821 | 36.67855 |
| $sd_{2}$ | 9.24905 | 17.37078 | 0.18729 | 4.47895 | 48.45307 |
| $sd_{3}$ | 10.93588 | 24.99543 | 0.18895 | 4.58145 | 61.90102 |
| ${z_{prob}}_{1}$ | 0.88770 | 0.05593 | 0.77385 | 0.88958 | 0.98688 |
| ${z_{prob}}_{2}$ | 0.93154 | 0.04292 | 0.83677 | 0.93677 | 0.99577 |
| ${z_{prob}}_{3}$ | 0.76884 | 0.04112 | 0.68574 | 0.76971 | 0.84650 |

Core management area: trends by year and season

Table D-2. Additional parameter estimates for the random walk model in the Core region. Parameters include: mean trends by year and season$\left( \mu\right)$, standard deviation of the random walk by year $\left( sd \right),$capture efficiency by year $\left( \theta\right)$, and the temporal suitability parameter from the zero inflation process by year $\left( z_{prob} \right).$

| Parameter | Mean | Sd | LCI | Median | UCI |
| --- | --- | --- | --- | --- | --- |
| $\mu_{1,1}$ | 19.08201 | 33.19404 | -46.708 | 19.82115 | 81.69742 |
| $\mu_{1,2}$ | 20.55622 | 31.11221 | -43.598 | 22.07794 | 77.13197 |
| $\mu_{1,3}$ | -11.9148 | 27.93881 | -67.2723 | -11.5027 | 43.44363 |
| $\mu_{1,4}$ | -0.34231 | 30.31044 | -60.0102 | -0.23868 | 58.78689 |
| $\mu_{2,1}$ | 30.95357 | 33.88494 | -37.0785 | 31.89182 | 94.64875 |
| $\mu_{2,2}$ | 28.11014 | 31.57923 | -36.6197 | 29.53966 | 85.40741 |
| $\mu_{2,3}$ | -4.67083 | 27.65172 | -59.5092 | -4.42033 | 49.47618 |
| $\mu_{2,4}$ | -0.09848 | 31.73068 | -62.1725 | -0.07007 | 62.09175 |
| $\mu_{3,1}$ | 12.4343 | 30.39355 | -47.7088 | 12.60437 | 71.63176 |
| $\mu_{3,2}$ | 40.49377 | 25.96749 | -17.3348 | 43.23143 | 85.1507 |
| $\mu_{3,3}$ | -4.18499 | 21.56786 | -49.9918 | -3.05605 | 35.93087 |
| $\mu_{3,4}$ | -13.7874 | 26.69376 | -64.191 | -14.7722 | 41.85238 |
| $sd_{1}$ | 12.08574 | 22.80226 | 0.194462 | 4.902815 | 76.81987 |
| $sd_{2}$ | 30.68991 | 85.62008 | 0.228226 | 6.085332 | 228.0759 |
| $sd_{3}$ | 10.1801 | 18.93452 | 0.185379 | 4.669537 | 56.14236 |
| $\theta_{1}$ | 9.67E-05 | 5.92E-05 | 3.08E-05 | 7.90E-05 | 0.000255 |
| $\theta_{2}$ | 0.000131 | 5.60E-05 | 4.09E-05 | 0.000124 | 0.000258 |
| $\theta_{3}$ | 0.000142 | 6.25E-05 | 4.34E-05 | 0.000133 | 0.000288 |
| ${z_{prob}}_{1}$ | 0.95456 | 0.024966 | 0.897395 | 0.957777 | 0.993103 |
| ${z_{prob}}_{2}$ | 0.770418 | 0.042822 | 0.682828 | 0.771926 | 0.849997 |
| ${z_{prob}}_{3}$ | 0.758366 | 0.043325 | 0.67042 | 0.759486 | 0.839836 |

East management area: trend by year

Table D-3. Additional parameter estimates for the random walk model in the East region. Parameters include: mean trends by year and season$\left( \mu\right)$, standard deviation of the random walk by year $\left( sd \right),$capture efficiency by year $\left( \theta\right)$, and the temporal suitability parameter from the zero inflation process by year $\left( z_{prob} \right).$

| Parameter | Mean | Sd | LCI | Median | UCI |
| --- | --- | --- | --- | --- | --- |
| $\mu_{1}$ | -0.55406 | 18.41555 | -44.505 | 2.600814 | 31.51248 |
| $\mu_{2}$ | 27.24034 | 19.9459 | -13.7846 | 25.72751 | 69.29992 |
| $sd_{1}$ | 23.60941 | 37.03975 | 0.573077 | 13.9099 | 104.9846 |
| $sd_{2}$ | 56.06835 | 223.786 | 0.658814 | 16.75442 | 271.1512 |
| $\theta_{1}$ | 0.00055 | 0.000591 | 4.00E-05 | 0.000336 | 0.002267 |
| $\theta_{2}$ | 0.00034 | 0.000307 | 3.46E-05 | 0.000248 | 0.001198 |
| ${z_{prob}}_{1}$ | 0.898958 | 0.068993 | 0.742859 | 0.909553 | 0.995193 |
| ${z_{prob}}_{2}$ | 0.95695 | 0.035711 | 0.867713 | 0.965759 | 0.998578 |

InfoPM Models

West management area: trend by year

Table D-4. Additional parameter estimates for the InfoPM in the West region. Parameters include: mean trends by year and season$\left( \mu\right)$, standard deviation of the random walk by year $\left( sd \right),$capture efficiency by year $\left( \theta\right)$, and the temporal suitability parameter from the zero inflation process by year $\left( z_{prob} \right)$ adult abundance at the birth pulse in year 0 $\left( {\lambda_{ad}}_{0} \right)$ adult abundance at the birth pulse in year y-1$\left( {\lambda_{ad}}_{y-1} \right)$, the effective birth rates each year $\left( b \right),$ prior for the effective birth rate $\left( b_{prior} \right)$ annual hatchling survival $(s_{H})$, prior for annual hatchling survival $(s_{H_{prior}})$, mean of the hatchling availability distribution $(\mu_{emerg})$, standard deviation of the hatchling distribution $({sd}_{emerge})$, the effective size of the hatchling cohort after one year $\left( B \right),$the size of the hatchling cohort immediately after the birth pulse $\left( H_{BP} \right)$, period survival rate of adults $(s)$, yearly survival rates of adults $\left( s_{y} \right)$, and yearly growth from net migration $(IE_{total})$.

| Parameter | Mean | Sd | LCI | Median | UCI |
| --- | --- | --- | --- | --- | --- |
| ${\lambda_{ad}}_{0}$ | 193.3794 | 240.2433 | 0.5256 | 109.0088 | 889.3570 |
| ${\lambda_{ad}}_{y-1}$ | 122.3181 | 70.6617 | 6.7895 | 119.8140 | 242.9333 |
| $\mu_{1}$ | 1.7846 | 13.1357 | -34.0398 | 4.9922 | 19.1566 |
| $\mu_{2}$ | 30.0549 | 17.2261 | -5.9791 | 29.5730 | 65.5059 |
| $\mu_{3}$ | 21.2711 | 19.3699 | -18.3256 | 20.8388 | 61.8660 |
| $\theta_{1}$ | 0.000185 | 0.000130 | 2.69E-05 | 0.000152 | 0.000511 |
| $\theta_{2}$ | 9.75E-05 | 5.12E-05 | 2.66E-05 | 8.78E-05 | 0.000223 |
| $\theta_{3}$ | 9.85E-05 | 5.46E-05 | 2.96E-05 | 8.62E-05 | 0.000239 |
| $b_{1}$ | 0.6739 | 0.4198 | 0.1801 | 0.5733 | 1.7837 |
| $b_{2}$ | 0.7913 | 0.4840 | 0.1976 | 0.6761 | 2.0520 |
| $b_{3}$ | 0.7200 | 0.4610 | 0.1894 | 0.6010 | 1.9499 |
| $b_{4}$ | 0.6096 | 0.3545 | 0.1679 | 0.5297 | 1.5230 |
| $b_{prior}$ | 0.7111 | 0.4617 | 0.1789 | 0.5944 | 1.9547 |
| $\mu_{emerge_{1}}$ | 179.9833 | 17.3074 | 151.5327 | 179.6667 | 208.4472 |
| $\mu_{emerge_{2}}$ | 180.3646 | 16.9889 | 151.8301 | 180.5169 | 208.4736 |
| $\mu_{emerge_{3}}$ | 178.4879 | 17.3683 | 151.3338 | 177.7217 | 208.2715 |
| ${s_{H}}_{1}$ | 0.2721 | 0.1650 | 0.0470 | 0.2390 | 0.6664 |
| ${s_{H}}_{2}$ | 0.3058 | 0.1726 | 0.0579 | 0.2754 | 0.7010 |
| ${s_{H}}_{3}$ | 0.2746 | 0.1683 | 0.0480 | 0.2401 | 0.6754 |
| ${s_{H}}_{4}$ | 0.2571 | 0.1571 | 0.0438 | 0.2254 | 0.6295 |
| ${s_{H}}_{prior}$ | 0.2772 | 0.1695 | 0.0476 | 0.2433 | 0.6784 |
| ${sd}_{emerge_{1}}$ | 61.8250 | 29.2558 | 8.7903 | 60.8410 | 121.6388 |
| ${sd}_{emerge_{2}}$ | 57.7181 | 25.4144 | 11.5258 | 56.2715 | 111.1014 |
| ${sd}_{emerge_{3}}$ | 75.5336 | 27.0642 | 19.6708 | 76.3036 | 127.8080 |

Table D-4. Continued

| Parameter | Mean | Sd | LCI | Median | UCI |
| --- | --- | --- | --- | --- | --- |
| $B_{1}$ | 82.1859 | 68.0293 | 3.0000 | 67.0000 | 253.0000 |
| $B_{2}$ | 150.5559 | 128.7413 | 21.0000 | 112.0000 | 505.0000 |
| $B_{3}$ | 166.1012 | 186.6592 | 22.0000 | 109.0000 | 660.0250 |
| $B_{4}$ | 282.9407 | 227.6319 | 49.0000 | 218.0000 | 883.0000 |
| $H_{BP_{1}}$ | 612.3701 | 677.9060 | 90.2840 | 402.1421 | 2371.6634 |
| $H_{BP_{2}}$ | 643.3432 | 673.3525 | 107.3700 | 433.6493 | 2493.9675 |
| $H_{BP_{3}}$ | 1340.8276 | 1157.1025 | 267.4166 | 1019.6964 | 4367.8051 |
| $s$ | 0.9795 | 0.0080 | 0.9609 | 0.9805 | 0.9920 |
| $s_{y}$ | 0.5952 | 0.1186 | 0.3547 | 0.5994 | 0.8120 |
| $s_{y_{prior}}$ | 0.5924 | 0.1199 | 0.3499 | 0.5966 | 0.8124 |
| ${z_{prob}}_{1}$ | 0.8887 | 0.0557 | 0.7742 | 0.8910 | 0.9872 |
| ${z_{prob}}_{2}$ | 0.9321 | 0.0431 | 0.8363 | 0.9373 | 0.9959 |
| ${z_{prob}}_{3}$ | 0.7685 | 0.0409 | 0.6858 | 0.7693 | 0.8457 |
| $I{E_{total}}_{1}$ | 57.6414 | 195.3074 | -494.5218 | 108.6311 | 293.6031 |
| $I{E_{total}}_{2}$ | 434.8409 | 198.4101 | 39.6343 | 424.9869 | 851.9610 |
| $I{E_{total}}_{3}$ | 406.8317 | 296.7092 | -210.5541 | 404.1774 | 1007.4431 |
| $sd_{1}$ | 6.9937 | 9.3687 | 0.1627 | 4.0608 | 32.6513 |
| $sd_{2}$ | 8.4077 | 12.6825 | 0.1837 | 4.3598 | 43.4720 |
| $sd_{3}$ | 11.0344 | 21.5351 | 0.1993 | 4.6510 | 67.0326 |

Core management areas: trend by year and season

Table D-5. Additional parameter estimates for the InfoPM in the Core region. Parameters include: mean trends by year and season$\left( \mu\right)$, standard deviation of the random walk by year $\left( sd \right),$capture efficiency by year $\left( \theta\right)$, and the temporal suitability parameter from the zero inflation process by year $\left( z_{prob} \right)$, adult abundance at the birth pulse in year 0 $\left( {\lambda_{ad}}_{0} \right)$, adult abundance at the birth pulse in year y-1$\left( {\lambda_{ad}}_{y-1} \right)$, the effective birth rates each year $\left( b \right),$ prior for the effective birth rate $\left( b_{prior} \right)$, annual hatchling survival $(s_{H})$, prior for annual hatchling survival $(s_{H_{prior}})$, mean of the hatchling availability distribution $(\mu_{emerg})$, standard deviation of the hatchling distribution $({sd}_{emerge})$, the effective size of the hatchling cohort after one year $\left( B \right),$the size of the hatchling cohort immediately after the birth pulse $\left( H_{BP} \right)$, period survival rate of adults $(s)$, yearly survival rates of adults $\left( s_{y} \right)$, and yearly growth from net migration $(IE_{total})$.

| Parameter | Mean | Sd | LCI | Median | UCI |
| --- | --- | --- | --- | --- | --- |
| ${\lambda_{ad}}_{0}$ | 301.4951 | 319.6932 | 0.5702 | 194.3205 | 1138.9032 |
| ${\lambda_{ad}}_{y-1}$ | 602.0462 | 403.1720 | 27.9650 | 551.0895 | 1418.1763 |
| $\mu_{1,1}$ | 18.3905 | 31.0642 | -42.8737 | 18.4894 | 77.5559 |
| $\mu_{1,2}$ | 26.7717 | 27.8849 | -32.0517 | 28.5889 | 76.8988 |
| $\mu_{1,3}$ | -16.3771 | 26.1288 | -68.5615 | -16.2771 | 36.1380 |
| $\mu_{1,4}$ | -5.0238 | 30.4583 | -65.1247 | -5.0696 | 54.7029 |
| $\mu_{2,1}$ | 28.6254 | 33.2257 | -38.8962 | 29.6947 | 89.2050 |
| $\mu_{2,2}$ | 27.7966 | 30.3994 | -35.6600 | 29.7069 | 81.8167 |
| $\mu_{2,3}$ | -10.9386 | 28.3025 | -66.5791 | -10.8461 | 44.1980 |
| $\mu_{2,4}$ | 0.0046 | 31.6380 | -61.5848 | -0.0585 | 62.4408 |
| $\mu_{3,1}$ | 9.0450 | 30.0895 | -51.3950 | 9.4271 | 66.5311 |
| $\mu_{3,2}$ | 38.4144 | 26.0670 | -18.5971 | 41.1761 | 83.3299 |
| $\mu_{3,3}$ | -8.9415 | 23.7515 | -58.1347 | -7.9325 | 36.4630 |
| $\mu_{3,4}$ | -17.2402 | 30.0290 | -74.6631 | -18.4550 | 43.1538 |
| $\theta_{1}$ | 0.0001277 | 6.24E-05 | 4.38E-05 | 0.0001157 | 0.0002792 |
| $\theta_{2}$ | 0.0001495 | 5.91E-05 | 5.38E-05 | 0.0001421 | 0.0002853 |
| $\theta_{3}$ | 0.0001368 | 6.42E-05 | 3.72E-05 | 0.0001282 | 0.0002859 |
| $b_{1}$ | 0.5870 | 0.3507 | 0.1687 | 0.5087 | 1.5120 |
| $b_{2}$ | 0.5802 | 0.3312 | 0.1597 | 0.5059 | 1.4318 |
| $b_{3}$ | 0.5771 | 0.3231 | 0.1671 | 0.5035 | 1.4359 |
| $b_{4}$ | 0.5760 | 0.3378 | 0.1606 | 0.4972 | 1.4577 |
| $b_{prior}$ | 0.7134 | 0.4633 | 0.1793 | 0.5939 | 1.9678 |
| $\mu_{emerge_{1}}$ | 180.1556 | 17.3658 | 151.5201 | 180.2181 | 208.5694 |
| $\mu_{emerge_{2}}$ | 183.0150 | 17.2540 | 152.0191 | 184.6259 | 208.8469 |
| $\mu_{emerge_{3}}$ | 180.0778 | 17.7650 | 151.3489 | 180.2211 | 208.6028 |

Table D-5. Continued

| Parameter | Mean | Sd | LCI | Median | UCI |
| --- | --- | --- | --- | --- | --- |
| ${s_{H}}_{1}$ | 0.2562 | 0.1570 | 0.0456 | 0.2238 | 0.6320 |
| ${s_{H}}_{2}$ | 0.2655 | 0.1562 | 0.0493 | 0.2360 | 0.6410 |
| ${s_{H}}_{3}$ | 0.2437 | 0.1489 | 0.0437 | 0.2125 | 0.6035 |
| ${s_{H}}_{4}$ | 0.2560 | 0.1611 | 0.0428 | 0.2231 | 0.6476 |
| ${s_{H}}_{prior}$ | 0.2776 | 0.1694 | 0.0478 | 0.2428 | 0.6777 |
| ${sd}_{emerge_{1}}$ | 60.1539 | 14.1972 | 32.5764 | 60.2015 | 88.0318 |
| ${sd}_{emerge_{2}}$ | 61.2155 | 15.2414 | 31.2202 | 61.2762 | 90.8634 |
| ${sd}_{emerge_{3}}$ | 66.1335 | 15.4815 | 33.9301 | 66.9462 | 94.5303 |
| $B_{1}$ | 315.7403 | 246.8499 | 13.0000 | 266.0000 | 926.0000 |
| $B_{2}$ | 332.1966 | 203.5182 | 71.0000 | 291.0000 | 840.0000 |
| $B_{3}$ | 288.9415 | 213.3889 | 65.0000 | 238.0000 | 841.0000 |
| $B_{4}$ | 231.4791 | 218.0359 | 40.0000 | 167.0000 | 862.0000 |
| $H_{BP_{1}}$ | 1581.1215 | 1268.5185 | 351.3644 | 1238.3436 | 4877.9228 |
| $H_{BP_{2}}$ | 1495.6045 | 1231.4768 | 322.7758 | 1157.7931 | 4732.9103 |
| $H_{BP_{3}}$ | 1078.9725 | 997.7116 | 214.3831 | 797.8845 | 3716.6512 |
| $I{E_{total}}_{1}$ | 114.7006 | 220.4798 | -328.8661 | 122.2895 | 521.8063 |
| $I{E_{total}}_{2}$ | 217.5024 | 238.2938 | -263.3327 | 229.5872 | 659.2653 |
| $I{E_{total}}_{3}$ | 89.9221 | 176.5706 | -283.4151 | 102.8256 | 419.0252 |
| $s$ | 0.9807 | 0.0074 | 0.9639 | 0.9816 | 0.9924 |
| $s_{y}$ | 0.6129 | 0.1134 | 0.3845 | 0.6164 | 0.8203 |
| $s_{y_{prior}}$ | 0.5936 | 0.1197 | 0.3497 | 0.5980 | 0.8133 |
| ${z_{prob}}_{1}$ | 0.9549 | 0.0248 | 0.8983 | 0.9582 | 0.9935 |
| ${z_{prob}}_{2}$ | 0.7700 | 0.0430 | 0.6812 | 0.7716 | 0.8489 |
| ${z_{prob}}_{3}$ | 0.7578 | 0.0434 | 0.6697 | 0.7589 | 0.8395 |
| $sd_{1}$ | 12.3158 | 23.7816 | 0.2102 | 4.7774 | 82.3136 |
| $sd_{2}$ | 19.3449 | 37.0763 | 0.2109 | 5.7437 | 134.0236 |
| $sd_{3}$ | 12.3739 | 23.4383 | 0.1897 | 4.9205 | 82.9697 |

East management area: trend by year

Table D-6. Additional parameter estimates for the InfoPM in the East region. Parameters include: mean trends by year and season$\left( \mu\right)$, standard deviation of the random walk by year $\left( sd \right),$capture efficiency by year $\left( \theta\right)$, and the temporal suitability parameter from the zero inflation process by year $\left( z_{prob} \right)$, adult abundance at the birth pulse in year 0 $\left( {\lambda_{ad}}_{0} \right)$, adult abundance at the birth pulse in year y-1$\left( {\lambda_{ad}}_{y-1} \right)$, the effective birth rates each year $\left( b \right),$ prior for the effective birth rate $\left( b_{prior} \right)$, annual hatchling survival $(s_{H})$, prior for annual hatchling survival $(s_{H_{prior}})$, mean of the hatchling availability distribution $(\mu_{emerg})$, standard deviation of the hatchling distribution $({sd}_{emerge})$, the effective size of the hatchling cohort after one year $\left( B \right),$the size of the hatchling cohort immediately after the birth pulse $\left( H_{BP} \right)$, period survival rate of adults $(s)$, yearly survival rates of adults $\left( s_{y} \right)$, and yearly growth from net migration $(IE_{total})$.

| Parameter | Mean | Sd | LCI | Median | UCI |
| --- | --- | --- | --- | --- | --- |
| ${\lambda_{ad}}_{0}$ | 127.5432 | 204.1158 | 0.1892 | 54.6284 | 777.9371 |
| ${\lambda_{ad}}_{y-1}$ | 71.6668 | 42.5632 | 3.7514 | 69.5442 | 145.3911 |
| $\mu_{1}$ | -0.4169 | 15.2977 | -38.3300 | 2.3165 | 23.3222 |
| $\mu_{2}$ | 28.3100 | 17.6963 | -2.0900 | 25.7568 | 68.2972 |
| $\theta_{1}$ | 0.000922 | 0.000727 | 6.79E-05 | 0.000736 | 0.002775 |
| $\theta_{2}$ | 0.000444 | 0.000368 | 7.31E-05 | 0.000332 | 0.0015 |
| $b_{1}$ | 0.6577 | 0.4013 | 0.1756 | 0.5621 | 1.6776 |
| $b_{2}$ | 0.5663 | 0.3432 | 0.1580 | 0.4863 | 1.4633 |
| $b_{3}$ | 0.6852 | 0.4546 | 0.1768 | 0.5647 | 1.9523 |
| $b_{prior}$ | 0.7126 | 0.4658 | 0.1773 | 0.5904 | 1.9689 |
| $\mu_{emerge_{1}}$ | 180.0507 | 17.3491 | 151.5355 | 180.0647 | 208.5146 |
| $\mu_{emerge_{2}}$ | 179.9592 | 17.4572 | 151.3880 | 180.0134 | 208.5420 |
| ${s_{H}}_{1}$ | 0.2661 | 0.1622 | 0.0456 | 0.2327 | 0.6477 |
| ${s_{H}}_{2}$ | 0.2405 | 0.1531 | 0.0406 | 0.2071 | 0.6161 |
| ${s_{H}}_{3}$ | 0.2683 | 0.1666 | 0.0458 | 0.2322 | 0.6695 |
| ${s_{H}}_{prior}$ | 0.2759 | 0.1678 | 0.0465 | 0.2433 | 0.6675 |
| ${sd}_{emerge_{1}}$ | 62.5982 | 29.6648 | 9.1517 | 61.5594 | 123.3630 |
| ${sd}_{emerge_{2}}$ | 75.1460 | 30.8280 | 12.6397 | 76.3206 | 133.6281 |
| $B_{1}$ | 44.9852 | 38.7280 | 1.0000 | 35.5000 | 144.0000 |
| $B_{2}$ | 86.4882 | 116.7047 | 9.0000 | 52.0000 | 420.0000 |
| $B_{3}$ | 143.5559 | 197.5673 | 14.0000 | 87.0000 | 645.0000 |
| $H_{BP_{1}}$ | 447.9080 | 635.3865 | 45.6258 | 255.0785 | 2098.7409 |
| $H_{B{}_{2}}$ | 639.4661 | 915.1364 | 71.1849 | 386.3116 | 2865.9536 |
| $s$ | 0.9789 | 0.0083 | 0.9599 | 0.9799 | 0.9919 |
| $s_{y}$ | 0.5872 | 0.1214 | 0.3448 | 0.5905 | 0.8094 |

Table D-6. Continued

| Parameter | Mean | Sd | LCI | Median | UCI |
| --- | --- | --- | --- | --- | --- |
| $s_{y_{prior}}$ | 0.5937 | 0.1194 | 0.3543 | 0.5970 | 0.8144 |
| ${z_{prob}}_{1}$ | 0.8992 | 0.0692 | 0.7410 | 0.9104 | 0.9951 |
| ${z_{prob}}_{2}$ | 0.9570 | 0.0358 | 0.8671 | 0.9658 | 0.9987 |
| $I{E_{total}}_{1}$ | -5.9781 | 168.8104 | -427.4641 | 26.0561 | 247.7010 |
| $I{E_{total}}_{2}$ | 374.5817 | 230.9367 | 2.4249 | 338.3425 | 904.4543 |
| $sd_{1}$ | 7.8092 | 11.9713 | 0.1479 | 4.1258 | 40.0299 |
| $sd_{2}$ | 8.3888 | 13.8927 | 0.1732 | 4.2604 | 42.7941 |

Model Selection and Fit

Table D-7. Model fit and parsimony for each set of model assumptions in each management area. Bayesian p values indicate a good fit for the top models (not close to 0 or 1), as does chat (values close to 1). The best RW models and their corresponding InfoPMs are marked with an asterisk for easy identification. Zero inflated models were always better than non-zero inflated versions. The seasonal trend model was preferred in the Core region, while the yearly trend model was preferred in the West and East management areas.

| Location | Model | ZIP | Trend | WAIC | Delta-WAIC |  | Bayes-p | chat |
| --- | --- | --- | --- | --- | --- | --- | --- | --- |
| *Core | RW | Yes | Season | 1426.805 | 0 |  | 0.309423 | 1.146987 |
| Core | RW | Yes | Year | 1428.247 | 1.442 |  | 0.313661 | 1.125949 |
| Core | RW | No | Season | 1782.15 | 355.345 |  | 0.007917 | 2.269338 |
| Core | RW | No | Year | 1781.866 | 355.061 |  | 0.007756 | 2.269887 |
|  |  |  |  |  |  |  |  |  |
| *Core | InfoPM | Yes | Season | 1427.515 | 0 |  | 0.317156 | 1.125008 |
| Core | InfoPM | Yes | Year | 1429.437 | 1.9222 |  | 0.320133 | 1.122815 |
| Core | InfoPM | No | Season | 1782.716 | 355.200 |  | 0.310933 | 1.128151 |
| Core | InfoPM | No | Year | 1782.022 | 354.507 |  | 0.008089 | 2.269261 |
|  |  |  |  |  |  |  |  |  |
| West | RW | Yes | Season | 1597.79 | 1.847 |  | 0.48035 | 1.011152 |
| *West | RW | Yes | Year | 1595.943 | 0 |  | 0.488461 | 1.008945 |
| West | RW | No | Season | 1757.213 | 161.27 |  | 0.063194 | 1.361325 |
| West | RW | No | Year | 1755.652 | 159.709 |  | 0.065342 | 1.360384 |
|  |  |  |  |  |  |  |  |  |
|  |  |  |  |  |  |  |  |  |
| West | InfoPM | Yes | Season | 1597.321 | 1.9734 |  | 0.48123 | 1.010868 |
| *West | InfoPM | Yes | Year | 1595.348 | 0 |  | 0.487989 | 1.008932 |
| West | InfoPM | No | Season | 1757.014 | 161.6667 |  | 0.064674 | 1.361073 |
| West | InfoPM | No | Year | 1755.775 | 160.4272 |  | 0.067259 | 1.361648 |
|  |  |  |  |  |  |  |  |  |
| East | RW | Yes | Season | 498.9993 | 0.2835 |  | 0.688169 | 0.919674 |
| *East | RW | Yes | Year | 498.7158 | 0 |  | 0.688925 | 0.918758 |
| East | RW | No | Season | 512.7444 | 14.0286 |  | 0.537529 | 0.995156 |
| East | RW | No | Year | 512.4308 | 13.715 |  | 0.537982 | 0.995048 |
|  |  |  |  |  |  |  |  |  |
| East | InfoPM | Yes | Season | 0.48123 | 0 |  | 0.692317 | 0.917348 |
| *East | InfoPM | Yes | Year | 0.487989 | 0.2839 |  | 0.6924 | 0.917139 |
| East | InfoPM | No | Season | 0.064674 | 19.0970 |  | 0.522222 | 1.002409 |
| East | InfoPM | No | Year | 0.067259 | 12.3977 |  | 0.644444 | 0.981135 |

Capture Probabilities


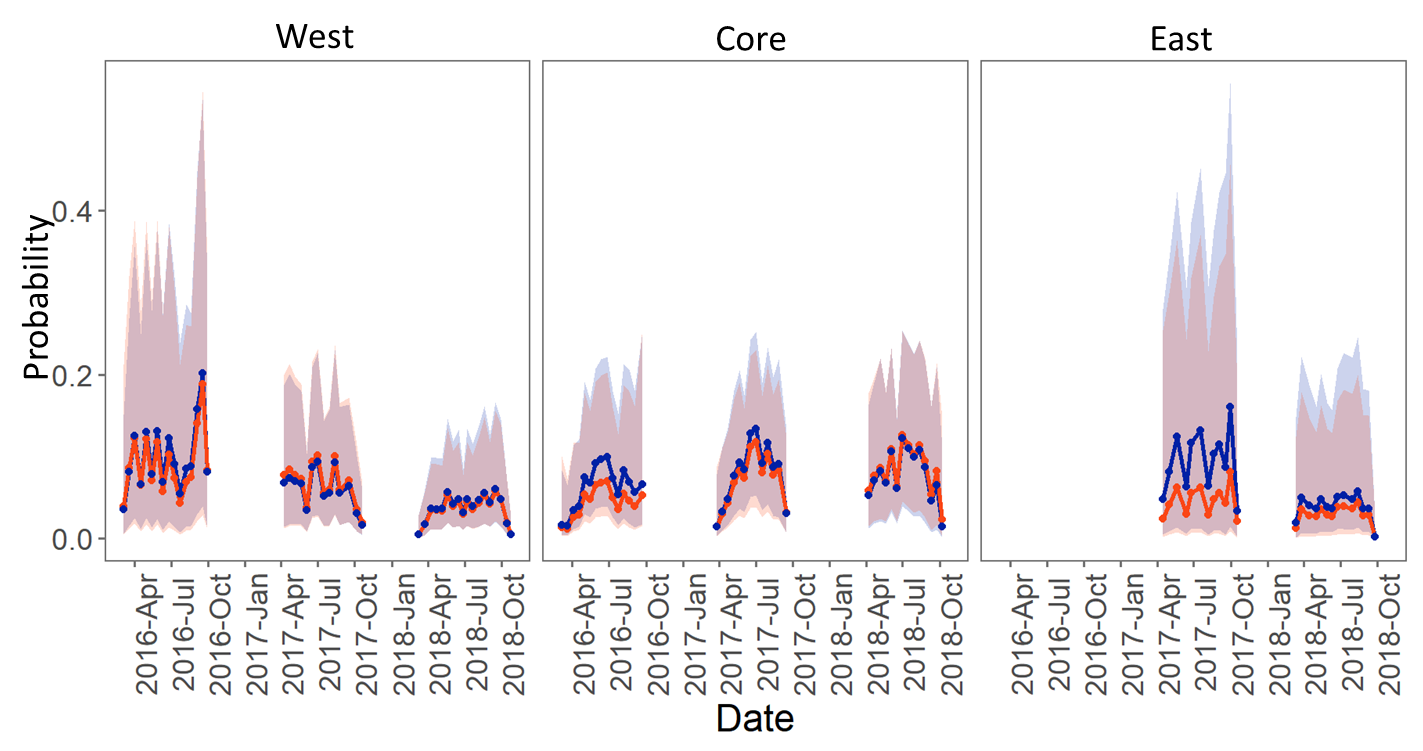


Figure D-1. Period-wide total capture probabilities each over time of the superpopulation after accounting for detectability, zero inflation and availability processes.

Growth Rate Estimates


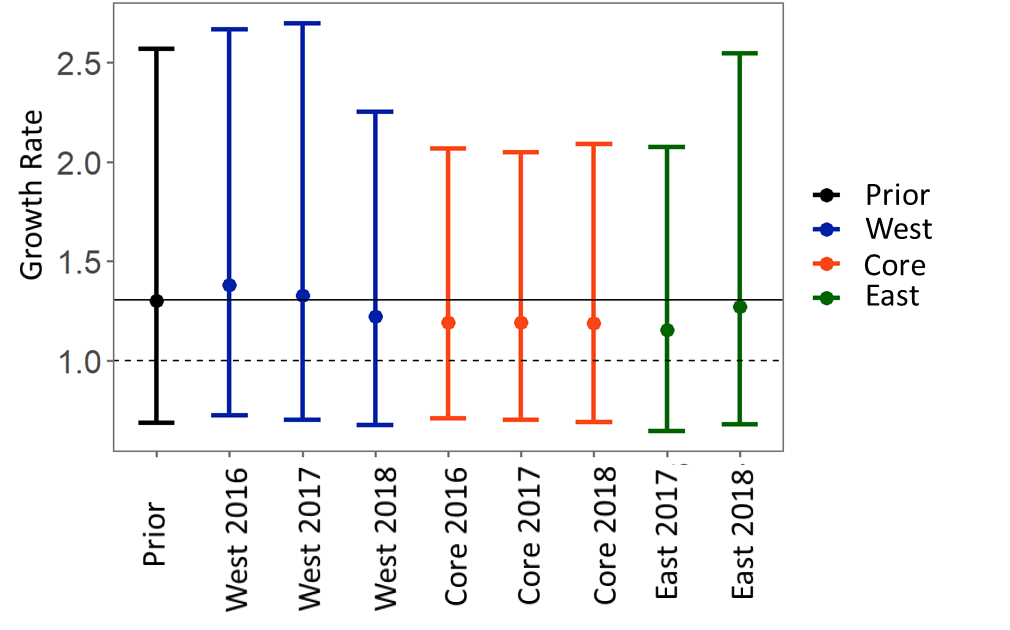


Figure D-2. Resolving uncertainty in the tegu growth rate belief distribution (prior) based on expert information using empirical removal data each year and management area. The growth rate is derived from scalar estimates for annual adult survival $s$ and birth rate each year $b_{y}$ as $s+b_{y}$. The posteriors strongly resemble the informative priors indicating the prior information is quite informative to the model, however some of the estimates, particularly those from Core region have considerable smaller CRIs and lower means than the priors, reflecting the update in the growth rate belief distribution based on empirical removal data.

**Appendix E: Graphical models and annotated JAGS code examples for the RW and InfoPM models**

*Graphical depictions of the RW and InfoPM models*


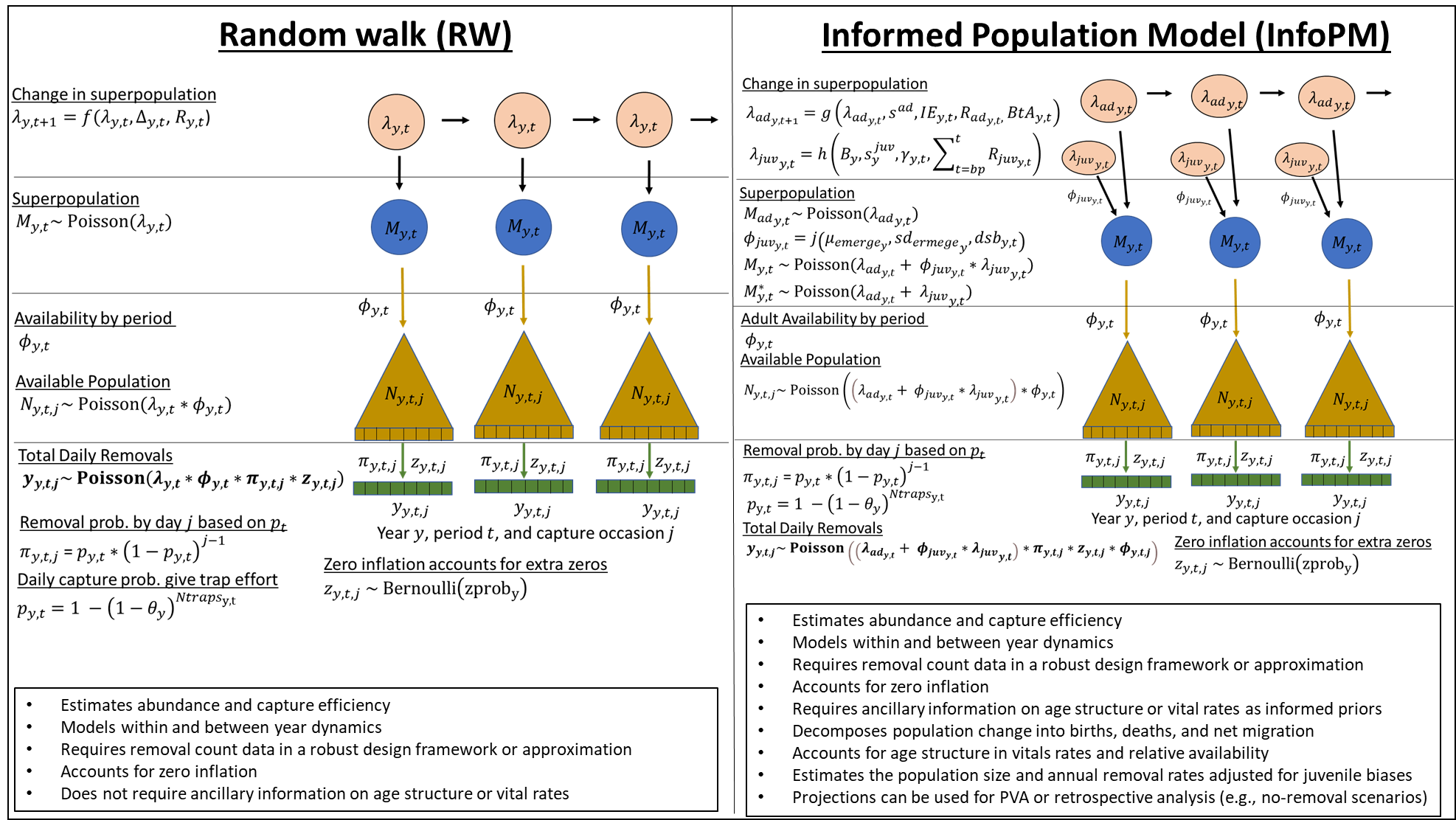


Figure E-1. Graphical representation of the hierarchical models and parameters for the random walk models (RW, left) and informed population models (InfoPM, right). The likelihood functions for each model are depicted in bold. In the RW model, the superpopulation dynamic function for the random walk, $f\left( \lambda_{y,t},\Delta_{y,t}, R_{y,t} \right)$, predicts $\lambda_{y,t+1}$, the expected superpopulation abundance year *y* and primary period *t+1* given: the expected superpopulation abundance in the primary period before $\left( \lambda_{y,t} \right)$, the change in abundance $\left( \Delta_{y,t} \right)$, and the total removals of each period $\left( R_{y,t} \right)$. In the InfoPM, the superpopulation dynamic function for the adult age class, $g\left( \lambda_{ad_{y,t}},s^{ad},IE_{y,t}, R_{ad_{y,t},}BtA_{y,t} \right)$, predicts the expected adult population at time *t+1* $\left( \lambda_{ad_{y,t+1}} \right)$given: the expected adult population the time step before $\left( \lambda_{ad_{y,t}} \right)$, adult survival scaled to the 2-week primary period $\left( s^{ad} \right)$, net migration $\left( IE_{y,t} \right)$, predicted adult removals $\left( R_{ad_{y,t},} \right)$, and the number of juveniles transitioning to adults $\left( BtA_{y,t} \right)$ which is zero unless *t* is the anniversary period of the birth pulse. Function $h\left( B_{y}, s_{y}^{juv},\gamma_{y,t},\sum_{t=bp}^{t} R_{{juv}_{y,t}} \right)$ makes a prediction for the expected number of juveniles each year and primary period $\left( \lambda_{{juv}_{y,t}} \right)$ based on the abundance of the effective juvenile cohort $\left( B_{y} \right)$(the number of juveniles born the previous year that also survive to become adults, before accounting for removals), the juvenile survival in year *y* $\left( s_{y}^{juv} \right)$, an exponential term $\left( \gamma_{y,t} \right)$ that scales juvenile abundance to the number surviving until year $y$ and primary period $t$ based on the number of primary periods since the birth pulse (Equation 27 in the main text), and a prediction for the total number of juveniles removed from the population since the birth pulse $\left( \sum_{t=bp}^{t} R_{{juv}_{y,t}} \right).$ $B_{y}$ is calculated based on the adult population at the time of birth pulse and the effective birth rate $\left( b_{y-1} \right)$ in year $y-1$, where the effective birth rate includes juvenile survival. Function $j\left( \mu_{emerge_{y}},s{d_{ermege}}_{y}, dsb_{y,t} \right)$ is a cumulative normal distribution with mean $\mu_{emerge_{y}}$ and standard deviation $s{d_{ermege}}_{y}$, which predicts the relative juvenile availability $\left( \phi_{juv_{y,t}} \right)$ each year and primary period given the number of days since the birth pulse $\left( dsb_{y,t} \right).$ See Methods section in the main text and Appendix B for more details.

**Random walk example**

*Parameter Definitions for the RW model*

Table E-1: Parameter definitions of each variable in the random walk model for the Core management area.

| **Parameter** | **Definition** |
| --- | --- |
| mu_trend1 | Vector of size 4, with the mean change (one for each season) in superpopulation each primary period in 2016. |
| mu_trend2 | Vector of size 4, with the mean change (one for each season) in superpopulation each primary period in 2017. |
| mu_trend3 | Vector of size 4, with the mean change (one for each season) in superpopulation each primary period in 2018. |
| theta | Vector of size 3, for the per unit effort daily removal probability each year (one for each year). |
| IE1 | Total change in superpopulation between primary periods from the random walk process for each primary period in 2016. |
| IE2 | Total change in superpopulation between primary periods from the random walk process for each primary period in 2017. |
| IE3 | Total change in superpopulation between primary periods from the random walk process for each primary period in 2018. |
| beta0 | The initial superpopulation size in the first primary period of 2016. |
| lambda | Poisson intensity for the superpopulation abundance for each primary period in 2016. |
| lambda2 | Poisson intensity for the superpopulation abundance for each primary period in 2017. |
| lambda3 | Poisson intensity for the superpopulation abundance for each primary period in 2018. |
| M | Superpopulation abundance state for each primary period in 2016. |
| M2 | Superpopulation abundance state for each primary period in 2017. |
| M3 | Superpopulation abundance state for each primary period in 2018. |
| phi | Availability for each primary period in 2016. |
| phi2 | Availability for each primary period in 2017. |
| phi3 | Availability for each primary period in 2018. |
| z_prob | Dailly temporal suitability (zero inflation) parameter for 2016. |
| z_prob2 | Dailly temporal suitability (zero inflation) parameter for 2017. |
| z_prob3 | Dailly temporal suitability (zero inflation) parameter for 2018. |
| N | Average available population abundance for each primary period in 2016. |
| N2 | Average available population abundance for each primary period in 2017. |
| N3 | Average available population abundance for each primary period in 2018. |
| p | Daily capture probability given trap effort and theta[1] each primary period in 2016. |
| p2 | Daily capture probability given trap effort and theta[2] each primary period in 2017. |
| p3 | Daily capture probability given trap effort and theta[3] each primary period in 2018. |
| pcap_super_eff | Total capture probability for superpopulation population each primary period in 2016. |
| pcap_super_eff2 | Total capture probability superpopulation population each primary period in 2017. |
| pcap_super_eff3 | Total capture probability superpopulation population each primary period in 2018. |
| fit1.data | Fit stats of the data to calculate posterior predictive checks for 2016. |
| fit1.pred | Fit stats of the predicted data to calculate posterior predictive checks for 2016. |
| fit2.data | Fit stats of the data to calculate posterior predictive checks for 2017. |
| fit2.pred | Fit stats of the predicted data to calculate posterior predictive checks for 2017. |
| fit3.data | Fit stats of the data to calculate posterior predictive checks for 2018. |
| fit3.pred | Fit stats of the predicted data to calculate posterior predictive checks for 2018. |
| lppd | Log likelihood of each data point in 2016. |
| lppd2 | Log likelihood of each data point in 2017. |
| lppd3 | Log likelihood of each data point in 2018. |

*Annotated JAGS code for the RW model (Core area)*

cat("
 model {

 #zero inflation parameter for 2016
 z_prob~ dbeta(1,1)

 #Periods in 2016
 for(i in 1:maxp){ # loop over primary periods
 phi[i]~ dbeta(1,1) #probability of availability each period

 M[i] ~ dpois(lambda[i]) # superpopulation abundance
 N[i] = M[i]* phi[i] # expectation for available abundance

 p[i] = 1-pow((1-theta[1]),ntraps[i]) #daily capture probability each period given total number of traps

 #total capture probability each period, not accounting for zero inflation or availability
 pcap[i] = 1-pow((1-p[i]),maxday[i])

 # Now also accounting for availability and zero inflation
 pcap_super_eff[i] = 1-pow((1-phi[i]*p[i]*z_prob),maxday[i])

 }

 # likelihood 2016
 # loop over capture days
 for(i in 1:nhists){

 z[i]~ dbern(z_prob) # zero inflation
 y[i] ~ dpois(e1[i]) # likelihood of captures given analytical Poisson expectation of the lambda and the multinomial capture probability
 y.pred[i] ~ dpois(e1[i])

 e1[i] = phi[period[i]]*pi[i]* lambda[period[i]] * z[i] #analytical Poisson expectation of the lambda and the multinomial capture probability

 pi[i] = p[period[i]]*pow(1-p[period[i]],capind[i]-1) #multinomial capture probability each capture day given period and capture day of each

 #monitor the likelihood and log-likelihood of each data point
 ppd[i] <- dpois(y[i],e1[i]) #likelihood for WAIC
 lppd[i] <- log(ppd[i])

 }


 # the change in superpopulation abundance between each primary period
 for(i in 2:maxp){

 lambda[i] = max( lambda[(i-1)]+lambda_change[(i-1)],0)

 lambda_change[i-1] = - R[i-1]+ IE1[i-1] #change includes removals from period before and change from RW

 }

 # Random walk process for each primary period in 2016
 for(i in 1:(maxp-1)){
 IE1[i] ~ dnorm(mu_trend1[indp1[i]],tau1)
 }


 #fit statistics 2016
 for(i in 1:nhists){
 resid1[i] = pow(pow(y[i],.5)-pow(e1[i],.5),2) #calc freeman tukey stat
 resid1.pred[i] = pow(pow(y.pred[i],.5)-pow(e1[i],.5),2) #calc freeman tukey stat

 }

 fit1.data = sum(resid1[])
 fit1.pred = sum(resid1.pred[])

 #Priors for the sd of the RW process each year
 #Half Cauchy priors (half t with 1 degree of freedom)
 tau1 ~ dscaled.gamma(5, 1) #2016
 tau2 ~ dscaled.gamma(5, 1) #2017
 tau3 ~ dscaled.gamma(5, 1) #2018

 sd1 = 1/sqrt(tau1)
 sd2 = 1/sqrt(tau2)
 sd3 = 1/sqrt(tau3)


 # per unit (trap) capture probability each year
 for(i in 1:3){

 theta[i] ~ dunif(0,1)

 }

 #mean trend each season and year
 for(i in 1:4){
 mu_trend1[i] ~ dnorm(0,0.001)
 mu_trend2[i] ~ dnorm(0,0.001)
 mu_trend3[i] ~ dnorm(0,0.001)

 }

 # Prior for abundance for the first primary period in 2016
 lambda[1] = beta0

 beta0 ~ dgamma(.5,0.00001)

 # Now repeat everything for 2017 and 2018
 ## 2017

 #likelihood
 z_prob2~ dbeta(1,1)

 for(i in 1:maxp2){
 phi2[i]~ dbeta(1,1)

 M2[i] ~ dpois(lambda2[i])
 N2[i] = M2[i]* phi2[i]

 p2[i] = 1-pow((1-theta[2]),ntraps2[i])
 pcap2[i] = 1-pow((1-p2[i]),maxday2[i])
 pcap_super2[i] = 1-pow((1-phi2[i]*p2[i]),maxday2[i])

 pcap_eff2[i] = 1-pow((1-p2[i]*z_prob2),maxday2[i])
 pcap_super_eff2[i] = 1-pow((1-phi2[i]*p2[i]*z_prob2),maxday2[i])
 }

 for(i in 1:nhists2){
 ppd2[i] <- dpois(y2[i],e2[i])
 lppd2[i] <- log(ppd2[i])

 z2[i]~ dbern(z_prob2)

 y2[i] ~ dpois(e2[i])
 y.pred2[i] ~ dpois(e2[i])

 e2[i] = phi2[period2[i]]*pi2[i]* lambda2[period2[i]]* z2[i]

 pi2[i] = p2[period2[i]]*pow(1-p2[period2[i]],capind2[i]-1)

 }

 for(i in 2:maxp2){

 lambda2[i] = max( lambda2[(i-1)]+lambda_change2[(i-1)], 0)

 lambda_change2[i-1] = - R2[i-1] + IE2[i-1]
 }

 for(i in 1:(maxp2-1)){
 IE2[i] ~ dnorm(mu_trend2[indp2[i]],tau2)
 }

 lambda2[1] = max(0,lambda[maxp] + YIE1)

 YIE1 ~ dnorm(0,0.001) #inter year change in abundance between last period of 2016 and first of 2017

 #fit statistics
 for(i in 1:nhists2){
 resid2[i] = pow(pow(y2[i],.5)-pow(e2[i],.5),2) #calc freeman tukey stat
 resid2.pred[i] = pow(pow(y.pred2[i],.5)-pow(e2[i],.5),2) #calc freeman tukey stat

 }

 fit2.data = sum(resid2[])
 fit2.pred = sum(resid2.pred[])

 # now do it all again for 2018
 ### 2018

 z_prob3~ dbeta(1,1)

 #liklihood
 for(i in 1:maxp3){
 phi3[i]~ dbeta(1,1)

 M3[i] ~ dpois(lambda3[i])
 N3[i] = M3[i]* phi3[i]

 p3[i] = 1-pow((1-theta[3]),ntraps3[i])
 pcap3[i] = 1-pow((1-p3[i]),maxday3[i])
 pcap_super3[i] = 1-pow((1-phi3[i]*p3[i]),maxday3[i])

 pcap_eff3[i] = 1-pow((1-p3[i]*z_prob3),maxday3[i])
 pcap_super_eff3[i] = 1-pow((1-phi3[i]*p3[i]*z_prob3),maxday3[i])

 }

 for(i in 1:nhists3){

 ppd3[i] <- dpois(y3[i],e3[i])
 lppd3[i] <- log(ppd3[i])

 z3[i]~ dbern(z_prob3)

 y3[i] ~ dpois(e3[i])
 y.pred3[i] ~ dpois(e3[i])

 e3[i] = phi3[period3[i]]*pi3[i]* lambda3[period3[i]]* z3[i]

 pi3[i] = p3[period3[i]]*pow(1-p3[period3[i]],capind3[i]-1)

 }


 for(i in 2:maxp3){

 lambda3[i] = max( lambda3[(i-1)]+lambda_change3[(i-1)],0)

 lambda_change3[i-1] = - R3[i-1] + IE3[i-1]

 }


 for(i in 1:(maxp3-1)){
 IE3[i] ~ dnorm(mu_trend3[indp3[i]],tau3)
 }

 lambda3[1] = max(0,lambda2[maxp2] + YIE2 )

 YIE2 ~ dnorm(0,0.001) #inter year change in abundance between last period of 2017 and first of 2018

 #fit statistics
 for(i in 1:nhists3){
 resid3[i] = pow(pow(y3[i],.5)-pow(e3[i],.5),2) #calc freeman tukey stat
 resid3.pred[i] = pow(pow(y.pred3[i],.5)-pow(e3[i],.5),2) #calc freeman tukey stat

 }

 fit3.data = sum(resid3[])
 fit3.pred = sum(resid3.pred[])

 }
 ",fill=TRUE,file="Core_Pois_ZIP_RW_YTseason.txt")

*Parameter definitions for the InfoPM*

Table E-2: Parameter definitions of each variable in the InfoPM model for the Core management area.

| **Parameter** | **Definition** |
| --- | --- |
| mu_trend1 | Vector of size 4, with the mean net migration rates each season in 2016. |
| mu_trend2 | Vector of size 4, with the mean net migration rates each season in 2017. |
| mu_trend3 | Vector of size 4, with the mean net migration rates each season in 2018. |
| theta | Vector of size 3, for the per unit effort daily removal probability each year (one for each year). |
| IE1 | Net migration in adult superpopulation in each primary period in 2016. |
| IE2 | Net migration in adult superpopulation in each primary period in 2017. |
| IE3 | Net migration in adult superpopulation in each primary period in 2018. |
| IE1_total | Total net migration in adult superpopulation in 2016. |
| IE2_total | Total net migration in adult superpopulation in 2017. |
| IE3_total | Total net migration in adult superpopulation in 2018. |
| sd1 | Sd of the random walk process for net migration in 2016. |
| sd2 | Sd of the random walk process for net migration in 2017. |
| sd3 | Sd of the random walk process for net migration in 2018. |
| b | Vector of size 4 with the scalar birth rate for the juvenile cohort from 2015-2016 [1], 2016-2017 [2], 2017-2018 [3], and 2018-2019 [4]. |
| b_prior | Prior for the scalar birth rate for comparison. |
| HS_year | Vector of size 4 with the annual juvenile survival probability in 2015-2016 [1], 2016-2017 [2], 2017-2018 [3], and 2018-2019 [4]. |
| HS_year_prior | Prior of juvenile survival for comparison. |
| B | The total effective juvenile cohort (from 2015-2016) accounting for annual juvenile survival, and also the number of new adults in 2016 (before subtracting juvenile removals since the birth pulse in 2015). |
| B2 | The total effective juvenile cohort (from 2016-2017) accounting for annual juvenile survival, and also the number of new adults in 2017 (before subtracting juvenile removals since the birth pulse in 2016). |
| B3 | The total effective juvenile cohort (from 2017-2018) accounting for annual juvenile survival, and also the number of new adults in 2018 (before subtracting juvenile removals since the birth pulse in 2017). |
| B4 | The total effective juvenile cohort (from 2018-2019) accounting for annual juvenile survival, and also the number of new adults in 2019 (before subtracting juvenile removals since the birth pulse in 2018). |
| beta0 | The initial adult population size in the first primary period of 2016. |
| lambda_ym1 | The population size of breeding adults (at the birth pulse) in year 2015, which is multiplied by the birth rate in 2015 to yield the juvenile cohort for the first half of 2016 |
| mu_emergeA | Mean of the cumulative normal distribution on juvenile availability (emergence/maturity) into the catch-able population given days since birth pulse (2016). |
| mu_emergeA2 | Mean of the cumulative normal distribution on juvenile availability (emergence/maturity) into the catch-able population given days since birth pulse (2017). |
| mu_emergeA3 | Mean of the cumulative normal distribution on juvenile availability (emergence/maturity) into the catch-able population given days since birth pulse (2018). |
| sd_emergeA | Sd of the cumulative normal distribution on juvenile availability (emergence/maturity) into the catch-able population given days since birth pulse (2016). |
| sd_emergeA2 | Sd of the cumulative normal distribution on juvenile availability (emergence/maturity) into the catch-able population given days since birth pulse (2017). |
| sd_emergeA3 | Sd of the cumulative normal distribution on juvenile availability (emergence/maturity) into the catch-able population given days since birth pulse (2018). |
| b_add | Prediction for the total number of juveniles alive and present in each primary period in 2016 that are also mature enough to be catch-able (i.e., available). |
| H1 | Prediction for the total number of juveniles alive and present in each primary period in 2016 that are NOT mature enough to be catch-able. |
| b_add + H1 | Total juveniles alive and present in each primary period in 2016. |
| b_add2 | Prediction for the total number of juveniles alive and present in each primary period in 2017 that are also mature enough to be catch-able |
| H2 | Prediction for the total number of juveniles alive and present in each primary period in 2017 that are NOT mature enough to be catch-able. |
| b_add2 + H2 | Total juveniles alive and present in each primary period in 2017. |
| b_add3 | Prediction for the total number of juveniles alive and present in each primary period in 2018 that are also mature enough to be catch-able |
| H3 | Prediction for the total number of juveniles alive and present in each primary period in 2018 that are NOT mature enough to be catch-able. |
| b_add3 + H3 | Total juveniles alive and present in each primary period in 2018. |
| surv | Adult survival scaled for each primary period (constant across all years). |
| surv_year | Annual adult survival (constant across all years). |
| surv_year_prior | Prior for annual adult survival (constant across all years) for comparison. |
| M_ad | Superpopulation abundance state of adults for each primary period in 2016. |
| M2_ad | Superpopulation abundance state of adults for each primary period in 2017. |
| M3_ad | Superpopulation abundance state of adults for each primary period in 2018. |
| lambda | Poisson intensity for the superpopulation abundance of adults for each primary period in 2016. |
| lambda2 | Poisson intensity for the superpopulation abundance of adults for each primary period in 2017. |
| lambda3 | Poisson intensity for the superpopulation abundance of adults for each primary period in 2018. |
| M | Superpopulation abundance state of catch-able animals (lambda + b_add) for each primary period in 2016. |
| M2 | Superpopulation abundance state of catch-able animals (lambda 2+ b_add2) for each primary period in 2017. |
| M3 | Superpopulation abundance state of catch-able animals (lambda3 + b_add3) for each primary period in 2018. |
| lambda_catch | Poisson expectation for the superpopulation abundance (M) of catch-able animals (lambda + b_add) for each primary period in 2016. |
| lambda2_catch | Poisson expectation for the superpopulation abundance (M2) of catch-able animals (lambda2 + b_add2) for each primary period in 2017. |
| Lambda3_catch | Poisson expectation for the superpopulation abundance (M3) of catch-able animals (lambda3 + b_add3) for each primary period in 2018. |
| lambda_all | Superpopulation of all animals (adults + juveniles) or (lambda + b_add + H1) each primary period in 2016. |
| lambda2_all | Superpopulation of all animals (adults + juveniles) or (lambd2a + b_add2 + H2) each primary period in 2017. |
| lambda3_all | Superpopulation of all animals (adults + juveniles) or (lambda3 + b_add3 + H3) each primary period in 2018. |
| phi | Adult availability for each primary period in 2016. |
| phi2 | Adult availability for each primary period in 2017. |
| phi3 | Adult availability for each primary period in 2018. |
| z_prob | Dailly temporal suitability (zero inflation) parameter for 2016. |
| z_prob2 | Dailly temporal suitability (zero inflation) parameter for 2017. |
| z_prob3 | Dailly temporal suitability (zero inflation) parameter for 2018. |
| N | Available population abundance of catch-able animals for each primary period in 2016. |
| N2 | Available population abundance of catch-able animals for each primary period in 2017. |
| N3 | Available population abundance of catch-able animals for each primary period in 2018. |
| R_ad1 | Prediction for the number of adults removed each primary period in 2016. |
| R_ad2 | Prediction for the number of adults removed each primary period in 2017. |
| R_ad3 | Prediction for the number of adults removed each primary period in 2018. |
| R_H1 | Prediction for the number of juveniles removed each primary period in 2016. |
| R_H2 | Prediction for the number of juveniles removed each primary period in 2017. |
| R_H3 | Prediction for the number of juveniles removed each primary period in 2018. |
| p_ad1 | Expected proportion of removals that are adults each primary period in 2016. |
| p_ad2 | Expected proportion of removals that are adults each primary period in 2017. |
| p_ad3 | Expected proportion of removals that are adults each primary period in 2018. |
| p_H1 | Expected proportion of removals that are juveniles each primary period in 2016. |
| p_H2 | Expected proportion of removals that are juveniles each primary period in 2017. |
| p_H3 | Expected proportion of removals that are juveniles each primary period in 2018. |
| p | Daily capture probability given trap effort each primary period in 2016. |
| p2 | Daily capture probability given trap effort each primary period in 2017. |
| p3 | Daily capture probability given trap effort each primary period in 2018. |
| pcap_super_eff | Total capture probability for the catch-able superpopulation population each primary period in 2016. |
| pcap_super_eff2 | Total capture probability for the catch-able superpopulation population each primary period in 2017. |
| pcap_super_eff3 | Total capture probability for the catch-able superpopulation population each primary period in 2018. |
| pcap_super_eff_all | Total capture probability for the superpopulation population each primary period in 2016 accounting for juv. availability and age distribution |
| pcap_super_eff_all2 | Total capture probability for the superpopulation population each primary period in 2017 accounting for juv. availability and age distribution |
| pcap_super_eff_all3 | Total capture probability for the superpopulation population each primary period in 2018 accounting for juv. availability and age distribution |
| catch_avail | The proportion of catch-able to non-catchable animals in the superpopulation (M_ad + b_add) / lambda_all each primary period in 2016. |
| catch_avail2 | The proportion of catch-able to non-catchable animals in the superpopulation (lambda2 + b_add2) / lambda2_all each primary period in 2017. |
| catch_avail3 | The proportion of catch-able to non-catchable animals in the superpopulation (lambda3 + b_add3) / lambda3_all each primary period in 2018. |
| lambda_rem | Adult superpopulation in the no-removal scenario for each primary period in 2016. |
| lambda_rem2 | Adult superpopulation in the no-removal scenario for each primary period in 2017. |
| lambda_rem3 | Adult superpopulation in the no-removal scenario for each primary period in 2018. |
| lambda_rem_catch | The catch-able superpopulation (adults + available juvs.) in the no-removal scenario for each primary period in 2016. |
| lambda_rem2_catch | The catch-able superpopulation (adults + available juvs.) in the no-removal scenario for each primary period in 2017. |
| lambda_rem3_catch | The catch-able superpopulation (adults + available juvs.) in the no-removal scenario for each primary period in 2018. |
| lambda_rem_all | Total superpopulation (adults + juvs.) in the no-removal scenario for each primary period in 2016. Also the expectation for M* in the no-removal scenario. |
| lambda2_rem_all | Total superpopulation (adults + juvs.) in the no-removal scenario for each primary period in 2017. Also the expectation for M2* in the no-removal scenario. |
| lambda3_rem_all | Total superpopulation (adults + juvs.) in the no-removal scenario for each primary period in 2018. Also the expectation for M3* in the no-removal scenario. |
| fit1.data | Fit stats of the data to calculate posterior predictive checks for 2016. |
| fit1.pred | Fit stats of the predicted data to calculate posterior predictive checks for 2016. |
| fit2.data | Fit stats of the data to calculate posterior predictive checks for 2017. |
| fit2.pred | Fit stats of the predicted data to calculate posterior predictive checks for 2017. |
| fit3.data | Fit stats of the data to calculate posterior predictive checks for 2018. |
| fit3.pred | Fit stats of the predicted data to calculate posterior predictive checks for 2018. |
| lppd | Log likelihood of each data point in 2016. |
| lppd2 | Log likelihood of each data point in 2017. |
| lppd3 | Log likelihood of each data point in 2018. |

# *JAGS code for the InfoPM (Core region)*

cat("
 model {

 # Year 2016 start

 # loop over all primary periods in year 1
 for(i in 1:maxp){ #maxp is the total number of periods in year 1

 phi[i]~ dbeta(1,1) #availability of each primary period in year 1


 #prediction for the catch-able superpopulation,
 #i.e., adults + juveniles that are large enough to respond to traps each primary period in year 1
 M[i] ~ dpois(lambda_catch[i])

 #prediction for the adult cohort (i.e., year 1 and older) superpopulation each primary period in year 1
 M_ad[i] ~ dpois(lambda[i])

 N[i] = M[i]* phi[i] #expectation for the available population each primary period in year 1
 N_ad[i] = M_ad[i]* phi[i] #expectation for the available population of adults (1yr and older) each primary period in year 1


 #capture probabilities
 #relationship between p and ntraps
 p[i] = 1-pow((1-theta[1]),ntraps[i]) #daily removal rate, before adjusting for availability zero-inflation


 }

 #Likelihood
 #loop over all trap nights i in year 1
 for(i in 1:nhists){

 # removal probability each capture day in year 1 given the capture day of each primary period
 # note that capind is the capture day of each primary period
 pi[i] = p[period[i]]*pow(1-p[period[i]],capind[i]-1)


 #expected value of the Poisson process (expected number of captures)
 e1[i] = phi[period[i]]*pi[i]* lambda_catch[period[i]] * z[i]
 z[i]~ dbern(z_prob) #daily zero inflation status for each capture night

 y[i] ~ dpois(e1[i]) #likelihood of the data

 #variables to calculate fit statistics and WAIC
 y.pred[i] ~ dpois(e1[i]) # prediction for PPC

 ppd[i] <- dpois(y[i],e1[i]) #likelihood calculation for WAIC
 lppd[i] <- log(ppd[i])

 }

 # Population dynamics between primary periods and additional variables needed for their calculation
 # there are generally 4 loops per year, 1: primary period 1, 2: primary periods 2:(birthpulse-1), 3: birthpulse, and 4) birthpulse+1 : end of year
 # When the birthpulse is in primary period 2 ...
 # there are 3 loops per year, 1: primary period 1, 2: birthpulse, and 4) birthpulse+1 : end of year
 # When the birthpulse is in primary period 3 ...
 # there are 4 loops per year, 1: primary period 1, 2:primary period 2, 3: birthpulse, and 4) birthpulse+1 : end of year


 # note that most variables have also been cloned to model a no-removal scenario
 # All variables corresponding to the no-removal scenario have '_rem' appended to the end
 # M, N, B (size of the birth cohort each year) are different

 # Now loop over the first primary period in year 1
 # All juveniles present are from the birth cohort in the year before
 # Note: after this first primary period, we must also include population dynamics
 for(i in 1){

 # probA: juvenile availability, i.e., the proportion of juveniles mature enough to capture given day of year
 # jdjlag[i] = total days since the birth pulse in the prior year
 # mu_emergeA: mean day since birth pulse for the juvenile availability, inflection point of the cumulative normal
 # tau_emergeA: precision of the cumulative normal function for juvenile availability

 probA[i] <- pnorm(jdjlag[i], mu_emergeA,tau_emergeA)

 # the total juveniles in the birth cohort that are catch-able (available) for the primary period
 b_add[i] = (B/HS_year[1]^((26-jdH1[i])/26)) * probA[i] # prediction for total juveniles, scaled by hatching survival, assuming 26 survival periods a year
 # next, do the same for the no-removal scenario
 b_add_rem[i] = (B_rem/HS_year[1]^((26-jdH1[i])/26)) *probA[i] #catch-able birth cohort in no removal scenario

 #now the total number of juveniles that are not catch-able (regular and no-removal scenarios)
 H1[i] = B/HS_year[1]^((26-jdH1[i])/26) - b_add[i] # number of juveniles that are not catch-able
 H1_rem[i] = B_rem/HS_year[1]^((26-jdH1[i])/26) - b_add_rem[i] #Total juveniles each primary period in the no-removal stage

 #total population size (adults + non-available juvs. + available juvs.)
 lambda_all[i] = lambda[i] + H1[i] + b_add[i] # size of the total population, adults + catch-able juvs + non-catch-able juvs.
 lambda_rem_all[i] = lambda_rem[i] + H1_rem[i] + b_add_rem[i] # size of total population (no removal) adults + juvs

 # the total size of the catch-able population (adults + available juvs.)
 lambda_catch[i] = lambda[i] + b_add[i] # the total number of animals that are available for capture
 lambda_rem_catch[i] = lambda_rem[i] + b_add_rem[i] # the total number of animals that are available for capture in no-removal

 #the proportion of catch-able to non-catch-able animals. Used to adjust the total annual capture rates, and allocate removals between adults and juveniles.
 catch_avail[i] = lambda_catch[i] / lambda_all[i] #the proportion of the population that is catch-able


 }


 # now do the repeat the loop, but from primary period 2 until the primary period just before the birthpulse
 # B_period1 = 4
 # now we incorporate population dynamics
 #note, we also track a prediction for
 # 1) the total number of juveniles, and
 # 2) the expected number of removals from each age class (Adults:R_ad1, Juveniles:R_H1)
 for(i in 2:(B_period1-1)){

 probA[i] <- pnorm(jdjlag[i], mu_emergeA,tau_emergeA)


 b_add[i] =(B/HS_year[1]^((26-jdH1[i])/26)) * probA[i]
 b_add_rem[i] = (B_rem/HS_year[1]^((26-jdH1[i])/26)) *probA[i]


 H1[i] = B/HS_year[1]^((26-jdH1[i])/26) - b_add[i]
 H1_rem[i] = B_rem/HS_year[1]^((26-jdH1[i])/26) - b_add_rem[i]

 lambda_all[i] = lambda[i] + H1[i] + b_add[i]- sum(R_H1[1:(i-1)]) #note, we also minus the expected number of the birth cohort that has been removed
 lambda_rem_all[i] = lambda_rem[i] + H1_rem[i] + b_add_rem[i]

 lambda_catch[i] = lambda[i] + b_add[i] - sum(R_H1[1:(i-1)])
 lambda_rem_catch[i] = lambda_rem[i] + b_add_rem[i]

 #the total proportion of the population that is catch-able
 catch_avail[i] = lambda_catch[i] / lambda_all[i]

 # now we include dynamics
 lambda[i] = max( lambda[(i-1)]+lambda_change[(i-1)],0)
 lambda_change[i-1] = - (lambda[(i-1)] - R_ad1[i-1])*(1-surv) - R_ad1[i-1]+ IE1[i-1]
 lambda_rem[i] = max(lambda_rem[(i-1)] - (1-surv)* lambda_rem[(i-1)] + IE1[i-1],0)

 # the proportion of the catch-able that animals are adults
 p_ad1[i-1] = (lambda[i-1]/(lambda[i-1] + b_add[i-1]))

 # the proportion of catch-able animals that are juveniles
 p_H1[i-1] = (b_add[i-1]/(lambda[i-1] + b_add[i-1]))

 # the expected number of adult removals
 R_ad1[i-1] = R[i-1]* p_ad1[i-1]

 # the expected number of juvenile removals
 R_H1[i-1] = R[i-1]*p_H1[i-1]


 }

 # B_period1 = 4
 # now loop over primary periods of the birth pulse
 # note, a new birth cohort is used from this primary period, until the primary period before the birth pulse next year
 for(i in B_period1){


 probA[i] <- pnorm(jdj1[i], mu_emergeA2,tau_emergeA2)

 #note, B2 is the birth cohort from 2016
 # HS_year[2]is the juv. survival for the 2016 birth cohort
 # jdH1 is the number of primary periods since birth, with 26 periods each year

 b_add[i] = (B2/HS_year[2]) * probA[i]
 b_add_rem[i] =(B2_rem/HS_year[2]) *probA[i]


 H1[i] = B2/HS_year[2] - b_add[i]
 H1_rem[i] = B2_rem/HS_year[2] -b_add_rem[i]

 # note that sum(R_H1[(B_period1-1):(i-1)]) is the total number of juveniles removed since the birth pulse, which is subtracted from the total number of animals.
 # Note that the total number of adults removed is accounted for in the dynamics for lambda each period which is a few lines down.

 lambda_all[i] = lambda[i] + H1[i] + b_add[i]
 lambda_rem_all[i] = lambda_rem[i] + H1_rem[i] + b_add_rem[i]

 lambda_catch[i] = lambda[i] + b_add[i]
 lambda_rem_catch[i] = lambda_rem[i] + b_add_rem[i]

 catch_avail[i] = lambda_catch[i] / lambda_all[i]

 #Transitioning juveniles to adults on the birth pulse period
 yr1s[i] = ifelse(i==B_period1,B - R_H1p_tot,0)
 yr1s_rem[i] = ifelse(i==B_period1,B_rem,0)

 lambda[i] = max( lambda[(i-1)]+lambda_change[(i-1)] ,0)
 # Note that - (lambda[(i-1)] - R_ad1[i-1])*(1-surv) subtracts mortalities, i.e., adults after removal * mortality rate
 # R_ad1[i-1] is the prediction for the total number of adults removed
 lambda_change[i-1] = - (lambda[(i-1)] - R_ad1[i-1])*(1-surv) - R_ad1[i-1]+ IE1[i-1] + yr1s[i]
 lambda_rem[i] = max(lambda_rem[(i-1)] - (1-surv)* lambda_rem[(i-1)] + IE1[i-1] +yr1s_rem[i] ,0)


 p_ad1[i-1] = (lambda[i-1]/(lambda[i-1] + b_add[i-1]))
 p_H1[i-1] = (b_add[i-1]/(lambda[i-1] + b_add[i-1]))


 R_ad1[i-1] = R[i-1]*p_ad1[i-1]
 R_H1[i-1] = R[i-1]*p_H1[i-1]


 }


 # loop from 1 period past the birth pulse unti the end period of 2016
 for(i in (B_period1+1):maxp){


 probA[i] <- pnorm(jdj1[i], mu_emergeA2,tau_emergeA2)

 #note, B2 is the birth cohort from 2016
 # HS_year[2]is the juv. survival for the 2016 birth cohort
 # jdH1 is the number of primary periods since birth, with 26 periods each year

 b_add[i] = (B2/HS_year[2]^((26-jdH1[i])/26)) * probA[i]
 b_add_rem[i] =(B2_rem/HS_year[2]^((26-jdH1[i])/26)) *probA[i]


 H1[i] = B2/HS_year[2]^((26-jdH1[i])/26) - b_add[i]
 H1_rem[i] = B2_rem/HS_year[2]^((26-jdH1[i])/26) -b_add_rem[i]

 # note that sum(R_H1[(B_period1-1):(i-1)]) is the total number of juveniles removed since the birth pulse, which is subtracted from the total number of animals.
 # Note that the total number of adults removed is accounted for in the dynamics for lambda each period which is a few lines down.

 # note that sum(R_H1[(B_period1):(i-1)]), or the expected number of juvs removed since B_period1 is subtracted from lambda_all and lambda_catch
 lambda_all[i] = lambda[i] + H1[i] + b_add[i] - sum(R_H1[(B_period1):(i-1)])
 lambda_rem_all[i] = lambda_rem[i] + H1_rem[i] + b_add_rem[i]

 lambda_catch[i] = lambda[i] + b_add[i] - sum(R_H1[(B_period1):(i-1)])
 lambda_rem_catch[i] = lambda_rem[i] + b_add_rem[i]

 catch_avail[i] = lambda_catch[i] / lambda_all[i]

 #Transitioning juveniles to adults on the birth pulse period
 #Note, these are all zero, but are kept for variable structure completeness
 yr1s[i] = ifelse(i==B_period1,B - R_H1p_tot,0)
 yr1s_rem[i] = ifelse(i==B_period1,B_rem,0)

 lambda[i] = max( lambda[(i-1)]+lambda_change[(i-1)] ,0)
 # Note that - (lambda[(i-1)] - R_ad1[i-1])*(1-surv) subtracts mortalities, i.e., adults after removal * mortality rate
 # R_ad1[i-1] is the prediction for the total number of adults removed
 lambda_change[i-1] = - (lambda[(i-1)] - R_ad1[i-1])*(1-surv) - R_ad1[i-1]+ IE1[i-1] + yr1s[i]
 lambda_rem[i] = max(lambda_rem[(i-1)] - (1-surv)* lambda_rem[(i-1)] + IE1[i-1] +yr1s_rem[i] ,0)


 p_ad1[i-1] = (lambda[i-1]/(lambda[i-1] + b_add[i-1]))
 p_H1[i-1] = (b_add[i-1]/(lambda[i-1] + b_add[i-1]))


 R_ad1[i-1] = R[i-1]*p_ad1[i-1]
 R_H1[i-1] = R[i-1]*p_H1[i-1]


 }

 # Prediction for total juveniles caught before and after bulse pulse each year
 R_H1p_tot = sum(R_H1[1:(B_period1-1)]) # pre-birth pulse 2016
 R_H1a_tot = sum(R_H1[B_period1:(maxp-1)]) # post-birth pulse 2016
 R_H2p_tot = sum(R_H2[1:(B_period2-1)]) # pre-birth pulse 2017
 R_H2a_tot = sum(R_H2[B_period2:(maxp2-1)]) # post-birth pulse 2017

 R_H3p_tot = sum(R_H3[1:(B_period3-1)]) # pre-birth pulse 2018
 # post birth pulse in 2018 not tracked here, but are included in the model

 for(i in 1:(maxp-1)){
 IE1[i] ~ dnorm(mu_trend1[indp1[i]],tau1)
 }


 lambda[1] = beta0
 lambda_rem[1] = beta0

 beta0 ~ dgamma(.5,0.00001) #Prior for the starting adult population size in the first primary period of year 1 (2016)


 lambda_ym1 ~ dunif(0,1500) #Prior for the starting adult population size at the birth pulse in year 0 (2015)

 B ~ dpois(b[1]*(lambda_ym1[1]) )# Birth cohort to start year 1, remnants from the cohort in year 0
 # lambda_ym1 is the size of the adult age class just before the birth pulse 2015, with all juveniles transitioned to adults
 B_rem ~ dpois( b[1]*lambda_ym1[1]) # Birth cohort for the no-removal scenario


 #now some derived parameters for the total removal rates with and with availability and zero-inflation
 # loop over primary periods in 2016 again
 for(i in 1:maxp){
 # total removal rate of the available population each primary period, before adjusting for zero-inflation
 pcap[i] = 1-pow((1-p[i]),maxday[i])


 #removal rate of the adult (1yr and older) superpopulation each primary period, after adjusting for availability
 pcap_super[i] = 1-pow((1-phi[i]*p[i]),maxday[i])

 #removal rate of the adult (1yr and older) superpopulation each primary period, after adjusting for zero-inflation
 pcap_eff[i] = 1-pow((1-p[i]*z_prob),maxday[i])


 #removal rate of the adult (1yr and older) superpopulation each primary period, after adjusting for availability and zero-inflation
 pcap_super_eff[i] = 1-pow((1-phi[i]*p[i]*z_prob),maxday[i])

 #removal rate of the superpopulation each primary period, after adjusting for availability, zero-inflation AND also ...
 # the proportion of catch-able population of the total population
 # note that catch_avail[i], which is defined in the model later, is the proportion of available pop. to total pop.
 # which is calculated as (adults[i] + juv_availability[i] * juveniles[i]) / (adults[i] + juveniles[i])

 pcap_super_eff_all[i] =1-pow((1-phi[i]*p[i]*z_prob* catch_avail[i]),maxday[i])

 }

 #fit statistics for 2016
 for(i in 1:nhists){
 resid1[i] = pow(pow(y[i],.5)-pow(e1[i],.5),2) #calc freeman tukey stat
 resid1.pred[i] = pow(pow(y.pred[i],.5)-pow(e1[i],.5),2) #calc freeman tukey stat

 }

 fit1.data = sum(resid1[])
 fit1.pred = sum(resid1.pred[])


 #priors
 # Model priors for all years
 #removal rate per unit effort each year
 #theta[1] is 2016, theta[2] is 2017, theta[3] is 2018
 for(i in 1:3){

 theta[i] ~ dunif(0,1)

 }


 #hatching survival rate for each primary period in year 1 based on the annual hatchling survival rate in year 1
 H_surv= pow(HS_year,1/26) #scale annual survival to period survival

 #annual survival rate of adult tegus
 surv_year ~ dbeta(sb1,sb2)
 surv_year_prior ~ dbeta(sb1,sb2) # simulate a prior to compare to the posterior

 surv= pow(surv_year,1/26) #scale adult survival in year 1


 #joint prior for the scalar, pre-birth pulse census birth rate and juvenile annual survival rate on link scales

 # first the correlation matrix that becomes the precision of the multivariate normal (e.g. Schaub & Kery (2021).)
 prec_mat[1:2,1:2] = inverse(covar_mat[1:2,1:2])

 #birth rate and juvenile survival priors, one for each year
 for(i in 1:4){
 ShAndb[i,1:2]~ dmnorm(mu_vec[1:2],prec_mat[1:2,1:2])

 b[i] = min(3,exp(ShAndb[i,1])) #ensure scalar birth rates cannot exceed the extreme value of 3, which is biologically unrealistic for vertbrate species .
 # e.g., if b =3, even very low survival probabilities (e.g. 0.1) yeilds a population growth rate of 3 + 0.1 = 3.1, or a doubling time of ~ 0.612 years!

 logit(HS_year[i]) = ShAndb[i,2] #transform juvenile (hatchling) survival
 }

 #a realization of the prior for reference, not included in the likelihood
 ShAndb_prior_part[1:2]~ dmnorm(mu_vec[1:2],prec_mat[1:2,1:2])
 b_prior <- min(3,exp(ShAndb_prior_part[1]))
 logit(HS_year_prior) = ShAndb_prior_part[2]


 # the total net migrants for 2016, sum over all primary periods
 IE1_total = sum(IE1[])

 #the trend each year and season of the RW net migration process
 for(i in 1:4){
 mu_trend1[i] ~ dnorm(0,0.001)

 }


 #Half cauchy priors (half t with 1 degree of freedom) for the sd of each RW net migration process each year across all seasons
 tau1 ~ dscaled.gamma(5, 1)
 sd1 = 1/sqrt(tau1)


 #temporal suitability (zero inflation parameter) in the daily capture rate
 z_prob~ dbeta(1,1) #2016

 #mean and sd of the juvenile availability distribution (cumulative normal) in 2015-2016 given days since the birth pulse
 mu_emergeA ~ dunif(150,210)
 tau_emergeA= 1/sd_emergeA^2
 sd_emergeA~dnorm(60,.005)T(0,) #sd of the culm norm distribution for hatchling emergence into capture-able pop


 #######
 ##END of year 2016
 #######################################
 YIE1 ~ dnorm(0,0.001) # net population change between last primary period of 2016 and first primary period of 2017


 #Priors for 2016 and 2017
 tau2 ~ dscaled.gamma(5, 1)
 tau3 ~ dscaled.gamma(5, 1)

 sd2 = 1/sqrt(tau2)
 sd3 = 1/sqrt(tau3)

 # Total net migration across all primary periods 2017 (IE2_total) and 2018 (IE3_total)
 IE2_total = sum(IE2[])
 IE3_total = sum(IE3[])

 # mean of seasonal trends for 2017 and 2018
 for(i in 1:4){
 mu_trend2[i] ~ dnorm(0,0.001)
 mu_trend3[i] ~ dnorm(0,0.001)

 }


 #mean and sd of the juvenile availability distribution (cumulative normal) in 2016-2017 given days since the birth pulse
 mu_emergeA2 ~ dunif(150,210)
 tau_emergeA2= 1/sd_emergeA2^2
 sd_emergeA2~dnorm(60,.005)T(0,)


 #mean and sd of the juvenile availability distribution (cumulative normal) in 2017-2018 and 2018-2019 given days since the birth pulse
 mu_emergeA3 ~ dunif(150,210)
 tau_emergeA3= 1/sd_emergeA3^2
 sd_emergeA3~dnorm(60,.005)T(0,)


 ## 2017


 #fit statistics
 for(i in 1:nhists2){
 resid2[i] = pow(pow(y2[i],.5)-pow(e2[i],.5),2) #calc freeman tukey stat
 resid2.pred[i] = pow(pow(y.pred2[i],.5)-pow(e2[i],.5),2) #calc freeman tukey stat

 }

 fit2.data = sum(resid2[])
 fit2.pred = sum(resid2.pred[])


 #liklihood
 z_prob2~ dbeta(1,1)

 for(i in 1:maxp2){
 phi2[i]~ dbeta(1,1)


 M2[i] ~ dpois(lambda2_catch[i])
 M2_ad[i] ~ dpois(lambda2[i])

 N2[i] = M2[i]* phi2[i]
 N2_ad[i] = M2_ad[i]* phi2[i]

 p2[i] = 1-pow((1-theta[2]),ntraps2[i])
 pcap2[i] = 1-pow((1-p2[i]),maxday2[i])
 pcap_super2[i] = 1-pow((1-phi2[i]*p2[i]),maxday2[i])

 pcap_eff2[i] = 1-pow((1-p2[i]*z_prob2),maxday2[i])
 pcap_super_eff2[i] = 1-pow((1-phi2[i]*p2[i]*z_prob2),maxday2[i])
 pcap_super_eff2_all[i] =1-pow((1-phi2[i]*p2[i]*z_prob2*catch_avail2[i]),maxday2[i])

 }

 for(i in 1:nhists2){
 ppd2[i] <- dpois(y2[i],e2[i])
 lppd2[i] <- log(ppd2[i])

 z2[i]~ dbern(z_prob2)

 y2[i] ~ dpois(e2[i])
 y.pred2[i] ~ dpois(e2[i])

 e2[i] = phi2[period2[i]]*pi2[i]* lambda2_catch[period2[i]]* z2[i]

 pi2[i] = p2[period2[i]]*pow(1-p2[period2[i]],capind2[i]-1)


 }


 # first primary period in 2017

 for(i in 1){


 probA2[i] <- pnorm(jdj1_lag[i], mu_emergeA2,tau_emergeA2)

 b_add2[i] = (B2/HS_year[2]^((26-jdH2[i])/26)) * probA2[i]
 b_add_rem2[i] = (B2_rem/HS_year[2]^((26-jdH2[i])/26)) * probA2[i]

 H2[i] = B2/HS_year[2]^((26-jdH2[i])/26) -b_add2[i]
 H2_rem[i] = B2_rem/HS_year[2]^((26-jdH2[i])/26) -b_add_rem2[i]

 # R_H1a_tot is the expected number of juvs. (2016-2017) removed after the birth pulse in 2016
 lambda2_all[i] = lambda2[i] + H2[i] + b_add2[i] - R_H1a_tot
 lambda2_rem_all[i] = lambda_rem2[i] + H2_rem[i] + b_add_rem2[i]

 lambda2_catch[i] = lambda2[i] + b_add2[i] - R_H1a_tot
 lambda2_rem_catch[i] = lambda_rem2[i] + b_add_rem2[i]

 catch_avail2[i] = lambda2_catch[i] / lambda2_all[i]


 }


 # Loop over period 2 (B_period2-1) which is the period before the birth pulse
 # B_period2 = 3
 for(i in 2:2){


 probA2[i] <- pnorm(jdj1_lag[i], mu_emergeA2,tau_emergeA2)

 b_add2[i] = (B2/HS_year[2]^((26-jdH2[i])/26)) * probA2[i]
 b_add_rem2[i] = (B2_rem/HS_year[2]^((26-jdH2[i])/26)) * probA2[i]

 H2[i] = B2/HS_year[2]^((26-jdH2[i])/26) -b_add2[i]
 H2_rem[i] = B2_rem/HS_year[2]^((26-jdH2[i])/26) -b_add_rem2[i]

 # R_H1a_tot is the expected number of juvs. (2016-2017) removed in the later part of 2016
 # sum(R_H2[1:(i-1)]) is the expected number of juvs. (2016-2017) removed before period i in 2017
 lambda2_all[i] = lambda2[i] + H2[i] + b_add2[i] - R_H1a_tot - sum(R_H2[1:(i-1)])
 lambda2_rem_all[i] = lambda_rem2[i] + H2_rem[i] + b_add_rem2[i]

 lambda2_catch[i] = lambda2[i] + b_add2[i] - R_H1a_tot - sum(R_H2[1:(i-1)])
 lambda2_rem_catch[i] = lambda_rem2[i] + b_add_rem2[i]


 catch_avail2[i] = lambda2_catch[i] / lambda2_all[i]

 lambda2[i] = max( lambda2[(i-1)]+lambda_change2[(i-1)],0)
 lambda_change2[i-1] = - (lambda2[(i-1)] - R_ad2[i-1])*(1-surv) - R_ad2[i-1]+ IE2[i-1]
 lambda_rem2[i] = max(lambda_rem2[(i-1)] - (1-surv)* lambda_rem2[(i-1)] + IE2[i-1] ,0)


 p_ad2[i-1] = (lambda2[i-1]/(lambda2[i-1] + b_add2[i-1]))
 p_H2[i-1] = (b_add2[i-1]/(lambda2[i-1] + b_add2[i-1]))


 R_ad2[i-1] = R2[i-1]* p_ad2[i-1]
 R_H2[i-1] = R2[i-1]* p_H2[i-1]


 }

 # Loop period of the birth pulse
 for(i in B_period2){


 probA2[i] <- pnorm(jdj2[i], mu_emergeA3,tau_emergeA3)


 b_add2[i] = (B3/HS_year[3]) * probA2[i]
 b_add_rem2[i] = (B3_rem/HS_year[3]) * probA2[i]

 H2[i] = B3/HS_year[3] -b_add2[i]
 H2_rem[i] = B3_rem/HS_year[3] - b_add_rem2[i]


 lambda2_all[i] = lambda2[i] + H2[i] + b_add2[i]
 lambda2_rem_all[i] = lambda_rem2[i] + H2_rem[i] + b_add_rem2[i]

 lambda2_catch[i] = lambda2[i] + b_add2[i]
 lambda2_rem_catch[i] = lambda_rem2[i] + b_add_rem2[i]

 catch_avail2[i] = lambda2_catch[i] / lambda2_all[i]


 yr1s2[i] = ifelse(i==B_period2,B2 – (R_H1a_tot - R_H2p_tot),0)
 yr1s2_rem[i] = ifelse(i==B_period2,B2_rem,0)


 lambda2[i] = max( lambda2[(i-1)]+lambda_change2[(i-1)],0)
 lambda_change2[i-1] = - (lambda2[(i-1)] - R_ad2[i-1])*(1-surv) - R_ad2[i-1]+ IE2[i-1] + yr1s2[i]
 lambda_rem2[i] = max(lambda_rem2[(i-1)] - (1-surv)* lambda_rem2[(i-1)] + IE2[i-1]+ yr1s2_rem[i] ,0)


 p_ad2[i-1] = (lambda2[i-1]/(lambda2[i-1] + b_add2[i-1]))
 p_H2[i-1] = (b_add2[i-1]/(lambda2[i-1] + b_add2[i-1]))


 R_ad2[i-1] = R2[i-1]* p_ad2[i-1]
 R_H2[i-1] = R2[i-1]*p_H2[i-1]


 }


 # loop from 1 period past the birth pulse until the last period of 2017

 for(i in (B_period2+1):maxp2){


 probA2[i] <- pnorm(jdj2[i], mu_emergeA3,tau_emergeA3)


 b_add2[i] = (B3/HS_year[3]^((26-jdH2[i])/26)) * probA2[i]
 b_add_rem2[i] = (B3_rem/HS_year[3]^((26-jdH2[i])/26)) * probA2[i]

 H2[i] = B3/HS_year[3]^((26-jdH2[i])/26) -b_add2[i]
 H2_rem[i] = B3_rem/HS_year[3]^((26-jdH2[i])/26) - b_add_rem2[i]

 # sum(R_H2[(B_period2):(i-1)]) is the expected number of juvs. (2017-2018) removed after the birth pulse and before period i in 2017
 lambda2_all[i] = lambda2[i] + H2[i] + b_add2[i] - sum(R_H2[(B_period2):(i-1)])
 lambda2_rem_all[i] = lambda_rem2[i] + H2_rem[i] + b_add_rem2[i]

 lambda2_catch[i] = lambda2[i] + b_add2[i] - sum(R_H2[(B_period2):(i-1)])
 lambda2_rem_catch[i] = lambda_rem2[i] + b_add_rem2[i]

 catch_avail2[i] = lambda2_catch[i] / lambda2_all[i]


 yr1s2[i] = ifelse(i==B_period2,B2 – (R_H1a_tot - R_H2p_tot),0)
 yr1s2_rem[i] = ifelse(i==B_period2,B2_rem,0)


 lambda2[i] = max( lambda2[(i-1)]+lambda_change2[(i-1)],0)
 lambda_change2[i-1] = - (lambda2[(i-1)] - R_ad2[i-1])*(1-surv) - R_ad2[i-1]+ IE2[i-1] + yr1s2[i]
 lambda_rem2[i] = max(lambda_rem2[(i-1)] - (1-surv)* lambda_rem2[(i-1)] + IE2[i-1]+ yr1s2_rem[i] ,0)


 p_ad2[i-1] = (lambda2[i-1]/(lambda2[i-1] + b_add2[i-1]))
 p_H2[i-1] = (b_add2[i-1]/(lambda2[i-1] + b_add2[i-1]))


 R_ad2[i-1] = R2[i-1]* p_ad2[i-1]
 R_H2[i-1] = R2[i-1]*p_H2[i-1]


 }

 for(i in 1:(maxp2-1)){
 IE2[i] ~ dnorm(mu_trend2[indp2[i]],tau2)
 }


 lambda2[1] = max(0,lambda[maxp] - (1-pow(surv,NPoff1))*lambda[maxp] + YIE1)
 lambda_rem2[1] = max(0,lambda_rem[maxp] - (1-pow(surv,NPoff1))*lambda_rem[maxp]+ YIE1)

 B2 ~ dpois(b[2]*(lambda[B_period1])) # Birth cohort 2016-2017
 B2_rem ~ dpois( b[2]*(lambda_rem[B_period1])) # Birth cohort no-removal scenario 2016-2017


 ### 2018


 #fit statistics
 for(i in 1:nhists3){
 resid3[i] = pow(pow(y3[i],.5)-pow(e3[i],.5),2) #calc freeman tukey stat
 resid3.pred[i] = pow(pow(y.pred3[i],.5)-pow(e3[i],.5),2) #calc freeman tukey stat

 }

 fit3.data = sum(resid3[])
 fit3.pred = sum(resid3.pred[])


 z_prob3~ dbeta(1,1)

 #liklihood
 for(i in 1:maxp3){
 phi3[i]~ dbeta(1,1)


 M3[i] ~ dpois(lambda3_catch[i])
 M3_ad[i] ~ dpois(lambda3[i])

 N3[i] = M3[i]* phi3[i]
 N3_ad[i] = M3_ad[i]* phi3[i]


 p3[i] = 1-pow((1-theta[3]),ntraps3[i])
 pcap3[i] = 1-pow((1-p3[i]),maxday3[i])
 pcap_super3[i] = 1-pow((1-phi3[i]*p3[i]),maxday3[i])

 pcap_eff3[i] = 1-pow((1-p3[i]*z_prob3),maxday3[i])
 pcap_super_eff3[i] = 1-pow((1-phi3[i]*p3[i]*z_prob3),maxday3[i])
 pcap_super_eff3_all[i] = 1-pow((1-phi3[i]*p3[i]*z_prob3*catch_avail3[i]),maxday3[i])


 }

 for(i in 1:nhists3){

 ppd3[i] <- dpois(y3[i],e3[i])
 lppd3[i] <- log(ppd3[i])

 z3[i]~ dbern(z_prob3)

 y3[i] ~ dpois(e3[i])
 y.pred3[i] ~ dpois(e3[i])

 e3[i] = phi3[period3[i]]*pi3[i]* lambda3_catch[period3[i]]* z3[i]

 pi3[i] = p3[period3[i]]*pow(1-p3[period3[i]],capind3[i]-1)


 }


 # first primary period in 2018

 for(i in 1){


 probA3[i] <- pnorm(jdj2_lag[i], mu_emergeA3,tau_emergeA3)


 b_add3[i] = (B3/HS_year[3]^((26-jdH3[i])/26)) * probA3[i]
 b_add_rem3[i] =(B3_rem/HS_year[3]^((26-jdH3[i])/26)) * probA3[i]

 # R_H2a_tot is the expected number of juvs. (2017-2018) removed in after the birth pulse in 2017

 lambda3_all[i] = lambda3[i] + H3[i] + b_add3[i] - R_H2a_tot
 lambda3_rem_all[i] = lambda_rem3[i] + H3_rem[i] + b_add_rem3[i]

 H3[i] = B3/HS_year[3]^((26-jdH3[i])/26) -b_add3[i]
 H3_rem[i] = B3_rem/HS_year[3]^((26-jdH3[i])/26) -b_add_rem3[i]

 lambda3_catch[i] = lambda3[i] + b_add3[i] - R_H2a_tot
 lambda3_rem_catch[i] = lambda_rem3[i] + b_add_rem3[i]


 catch_avail3[i] = lambda3_catch[i] / lambda3_all[i]


 }


 #B_period3 = 2
 # Loop period of the birth pulse
 for(i in B_period3){

 #Note, because we dont have data to inform mu_emergeA4 in the second half of 2018,
 # assume the same culm. normal distribution as the year before (i.e., using mu_emergeA3 )
 probA3[i] <- pnorm(jdj3[i], mu_emergeA3,tau_emergeA3)


 b_add3[i] = (B4/HS_year[4]) * probA3[i]
 b_add_rem3[i] = (B4_rem/HS_year[4]) * probA3[i]

 lambda3_all[i] = lambda3[i] + H3[i] + b_add3[i]
 lambda3_rem_all[i] = lambda_rem3[i] + H3_rem[i] + b_add_rem3[i]

 H3[i] = B4/HS_year[4] - b_add3[i]
 H3_rem[i] = B4_rem/HS_year[4] - b_add_rem3[i]

 lambda3_catch[i] = lambda3[i] + b_add3[i]
 lambda3_rem_catch[i] = lambda_rem3[i] + b_add_rem3[i]

 catch_avail3[i] = lambda3_catch[i] / lambda3_all[i]


 lambda3[i] = max( lambda3[(i-1)]+lambda_change3[(i-1)],0)


 yr1s3[i] = ifelse(i==B_period3,B3 – (R_H2a_tot - R_H3p_tot),0)
 yr1s3_rem[i] = ifelse(i==B_period3,B3_rem,0)


 lambda_change3[i-1] = - (lambda3[(i-1)] - R_ad3[i-1])*(1-surv)- R_ad3[i-1] + IE3[i-1] + yr1s3[i]
 lambda_rem3[i] = max( lambda_rem3[(i-1)] - (1-surv)* lambda_rem3[(i-1)] + IE3[i-1]+ yr1s3_rem[i], 0)


 p_ad3[i-1] = (lambda3[i-1]/(lambda3[i-1] + b_add3[i-1]))
 p_H3[i-1] = (b_add3[i-1]/(lambda3[i-1] + b_add3[i-1]))

 R_ad3[i-1] = R3[i-1]*p_ad3[i-1]
 R_H3[i-1] = R3[i-1]* p_H3[i-1]


 }


# Loop from period of the birth pulse until the last primary period of the year
 for(i in (B_period3+1):maxp3){

 #Note, because we dont have data for 2016 to inform mu_emergeA4 in the second half of 2018,
 # assume the same mean and sd as the year before (i.e., using mu_emergeA3 )
 probA3[i] <- pnorm(jdj3[i], mu_emergeA3,tau_emergeA3)


 b_add3[i] = (B4/HS_year[4]^((26-jdH3[i])/26)) * probA3[i]
 b_add_rem3[i] = (B4_rem/HS_year[4]^((26-jdH3[i])/26)) * probA3[i]

 # sum(R_H3[(B_period3):(i-1)]) is the expected number of juvs. removed after the birthpulse in 2018 and before period i
 lambda3_all[i] = lambda3[i] + H3[i] + b_add3[i] - sum(R_H3[(B_period3):(i-1)])
 lambda3_rem_all[i] = lambda_rem3[i] + H3_rem[i] + b_add_rem3[i]

 H3[i] = B4/HS_year[4]^((26-jdH3[i])/26) - b_add3[i]
 H3_rem[i] = B4_rem/HS_year[4]^((26-jdH3[i])/26) - b_add_rem3[i]

 lambda3_catch[i] = lambda3[i] + b_add3[i]- sum(R_H3[(B_period3):(i-1)])
 lambda3_rem_catch[i] = lambda_rem3[i] + b_add_rem3[i]

 catch_avail3[i] = lambda3_catch[i] / lambda3_all[i]


 lambda3[i] = max( lambda3[(i-1)]+lambda_change3[(i-1)],0)


 yr1s3[i] = ifelse(i==B_period3,B3 - (R_H2a_tot - R_H3p_tot),,0)
 yr1s3_rem[i] = ifelse(i==B_period3,B3_rem,0)


 lambda_change3[i-1] = - (lambda3[(i-1)] - R_ad3[i-1])*(1-surv)- R_ad3[i-1] + IE3[i-1] + yr1s3[i]
 lambda_rem3[i] = max( lambda_rem3[(i-1)] - (1-surv)* lambda_rem3[(i-1)] + IE3[i-1]+ yr1s3_rem[i], 0)


 p_ad3[i-1] = (lambda3[i-1]/(lambda3[i-1] + b_add3[i-1]))
 p_H3[i-1] = (b_add3[i-1]/(lambda3[i-1] + b_add3[i-1]))

 R_ad3[i-1] = R3[i-1]*p_ad3[i-1]
 R_H3[i-1] = R3[i-1]* p_H3[i-1]


 }

 for(i in 1:(maxp3-1)){
 IE3[i] ~ dnorm(mu_trend3[indp3[i]],tau3)
 }


 lambda3[1] = max(0,lambda2[maxp2] - (1-pow(surv,NPoff2))*lambda2[maxp2]+ YIE2 )
 lambda_rem3[1] = max(0,lambda_rem2[maxp2] - (1-pow(surv,NPoff2))*lambda_rem2[maxp2]+ YIE2 )


 B3 ~ dpois( b[3]*(lambda2[B_period2] )) # Birth cohort
 B3_rem ~ dpois( b[3]*(lambda_rem2[B_period2])) # Birth cohort

 B4 ~ dpois( b[4]*(lambda3[B_period3])) # Birth cohort
 B4_rem ~ dpois( b[4]*(lambda_rem3[B_period3])) # Birth cohort


 YIE2 ~ dnorm(0,0.001) #inter year immagration and emigration

 mu_emerge3 ~ dunif(30,90)
 tau_emerge3= 1/sd_emerge3^2
 sd_emerge3~dnorm(30,.001)T(0,)


 }
 ",fill=TRUE,file="Pois_ZIP_Core_InfoPM_Seasonal.txt")
